# *PDGF* Family Expression in Glioblastoma Multiforme: Data Compilation from Ivy Glioblastoma Atlas Project Database

**DOI:** 10.1038/s41598-017-15045-w

**Published:** 2017-11-10

**Authors:** Isabella Gomes Cantanhede, João Ricardo Mendes de Oliveira

**Affiliations:** 1 Laboratório de Neuroimunogenética, Laboratório de Imunopatologia Keizo Asami, Universidade Federal de Pernambuco, Recife, Brazil; 2 Departamento de Neuropsiquiatria, Universidade Federal de Pernambuco, Recife, Brazil

## Abstract

Glioblastoma Multiforme (GBM) is the most frequent and lethal primary brain cancer. Due to its therapeutic resistance and aggressiveness, its clinical management is challenging. Platelet-derived Growth Factor (PDGF) genes have been enrolled as drivers of this tumour progression as well as potential therapeutic targets. As detailed understanding of the expression pattern of *PDGF* system in the context of GBM intra- and intertumoral heterogeneity is lacking in the literature, this study aims at characterising *PDGF* expression in different histologically-defined GBM regions as well as investigating correlation of these genes expression with parameters related to poor prognosis. Z-score normalised expression values of PDGF subunits from multiple slices of 36 GBMs, alongside with clinical and genomic data on those GBMs patients, were compiled from Ivy Glioblastoma Atlas Project – Allen Institute for Brain Science data sets. PDGF subunits show differential expression over distinct regions of GBM and *PDGF* family is heterogeneously expressed among different brain lobes affected by GBM. Further, *PDGF* family expression correlates with bad prognosis factors: age at GBM diagnosis, Phosphatase and Tensin Homolog deletion and Isocitrate Dehydrogenase 1 mutation. These findings may aid on clinical management of GBM and development of targeted curative therapies against this devastating tumour.

## Introduction

Glioblastoma multiforme (GBM) is the most frequent and the worst type of glioma, in terms of therapeutic resistance, aggressiveness and associated short life expectancy. At present, management of GBMs is based on surgical resection followed by chemotherapy and/or radiotherapy, which is invasive, comes with unwanted side effects, and does not prevent the tumour from being significantly recurrent^1,2^. Therefore, efforts have been directed to the characterisation of the molecular and genetic profile of this high grade glioma and the discovery of therapeutic markers that may be the targets of a more specific and effective clinical approach^3^.

In this context, Platelet-derived Growth Factors (PDGF) constitute a family of six subunits assembled into heterodimer and homodimer ligands and tyrosine kinase receptors, which are enrolled in physiological embryogenesis, haematopoiesis, neuroprotection and glial cell development besides of being identified as part of the GBM molecular panel. These genes have been described as drivers of GBM growth and metastasis, for their role in the dedifferentiation of glial cells into stem cells, the epithelial to mesenchymal transition, the activation of cancer-associated fibroblasts as well as the intratumoral stimulation of angiogenesis, lymphangiogenesis and immunosuppression^4,5^. Of clinical importance, *PDGF* system expression and genetic profiles have been reported as prognostic factors and valid therapeutic response biomarkers for a number of cancers, such as sarcomas and breast cancer, and these properties are also been investigated on brain tumors^6^.

For their prominent influence in GBM development, and in keeping with the perspective of molecular-based curative treatments of this tumour, PDGF genes have been suggested as GBM therapeutic targets and clinical trials have been performed with inhibitors of tyrosine kinase receptors such as Imatinib, which showed only limited beneficial effects. Treatment failure may be attributed to the lack of specificity of these inhibitors as to PDGF receptors^5,7^. Further, heterogeneity is a hallmark of GBM^8^ and is thought not to be appreciated by current diagnostic and assessment method of single biopsy and whole tissue analysis of a GBM^9^. This is likely to give a poor perspective of how is the tumour molecular panel configured and how its already well-described biomarkers are distributed in each single tumour block, which has a direct negative impact on the development of successful targeted therapies.

To motivate further research on GBM, the Ivy Glioblastoma Atlas Project [© 2015 Allen Institute for Brain Science. Ivy Glioblastoma Atlas Project. Available from: http://glioblastoma.alleninstitute.org/] is a scientific initiative that investigates the expression of selected genes on different histological regions of GBM blocks, gathers genomic profile and clinical data of the correspondent patients/donors and publishes this valuable resource in the free-access online platforms of Allen Brain Atlas and Ivy GAP Clinical and Genomic Database. In the present study, *PDGF* system expression data is compiled from those databases with the aim to characterise these genes distribution in different histological regions of a GBM, so that more specific curative strategies against this tumour may be devised. Moreover, *PDGF* expression is studied regarding clinical parameters that influence life expectancy of GBM patients, to evaluate the potential role of those genes as prognostic factors in GBM.

## Results

### Clinical and genomic data on GBM patients herein studied

Information gathered from the online Ivy GAP Clinical and Genomic Database and summarised in Table 1 reveal that the patients who donated the GBM blocks analysed by Allen Institute organisation constituted a young population at the time of diagnosis, with no substantially differential distribution between sexes. Most subjects presented high Karnofsky Performance Status score, which indicates mildly compromised functionality/quality of life and favourable prognosis^10–12^. Nevertheless, average overall survival period illustrates the dramatically shortened life expectancy associated with GBM diagnosis. All patients were treated by the standard combination of surgical tumour resection plus radiotherapy and/or chemotherapy. Rates of Phosphatase and Tensin Homolog (*PTEN)* loss and Isocitrate Dehydrogenase 1 (*IDH1)* mutation at R132 were consistent with frequencies reported in the literature: *PTEN* deletion is considered to be a driver alteration very commonly associated with GBM^13^, whereas *IDH1* mutation is described as being much more prevalent in secondary GBM, rather than in primary tumours^14^, which are being studied here. More extensive details on each donor’s clinical profile and disease progression have been tabulated and are shown in the Supplementary Table 1.

**Table 1:** Clinical and Genomic data on subjects gathered for this study. *PTEN*: Phosphatase and Tensin Homolog; *IDH1*: Isocitrate Dehydrogenase 1. Mutations in IDH1 were R132H and R132G substitutions.

Clinical Data	N	%
**Gender**		
*Female*	17	47,2
*Male*	19	52,8
**Age at diagnosis**		
>*65 years-old*	9	25,0
≤*65 years-old*	27	75,0
**Karnofsky Performance Status**–**KPS**		
>70	30	83,3
≤70	6	16,7
**1st Tumour location**		
*Right Frontal Lobe*	8	22,2
*Left Frontal Lobe*	1	2,8
*Right Parietal Lobe*	5	13,9
*Left Parietal Lobe*	4	11,1
*Right Temporal Lobe*	7	19,4
*Left Temporal Lobe*	5	13,9
*Right Frontal-Temporal Lobes*	2	5,6
*Right Occipital-Parietal Lobes*	1	2,8
*Right Occipital-Temporal Lobes*	1	2,8
*Right Parietal-Temporal Lobes*	1	2,8
*Left Occipital Lobe*	1	2,8
**Overall survival after diagnosis**	**Mean**	**Moda**
	471 days	300 days
**Genomic data**	**N**	**%**
***PTEN***		
*Deletion/Loss*	21	58,3
*Gain*	3	8,3
*Normal*	3	8,3
***IDH1***		
*Wild-type*	32	88,9
*Mutated*	3	8,3

### *PDGF* family shows varied and heterogenic expression patterns among the GBM regions

**Figure 1:**
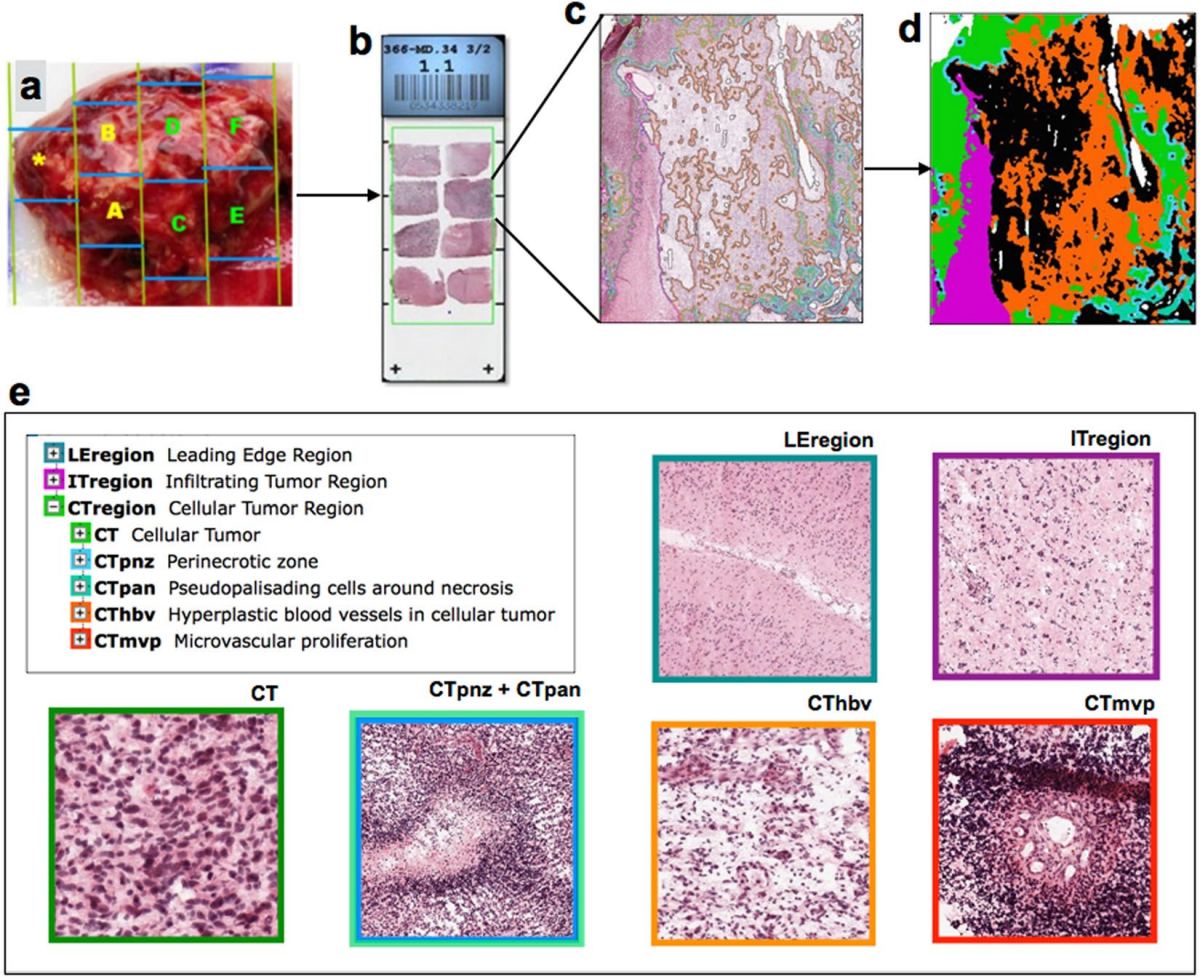
Workflow of Ivy Glioblastoma Atlas Project data production. Each surgically resected tumour is divided into blocks (**a**), which are sectioned into histological slices (**b**). Each section is submitted to histopathological evaluation (**c**), with annotation (**d**) of the distinct GBM histological regions (**e**), which are then processed by laser-microdissection and analysed separately by RNA-sequencing technique. As defined in the Technical White Paper: Overview – 2015 [accessible in glioblastoma.alleninstitute.org], Leading Edge Region: border of the tumour, tumour/normal cells ratio is approximately 1–3/100; Infiltrating Tumour: region in between Leading Edge and Cellular Tumour bulk, tumour/normal cells ratio is approximately 10–20/100; Cellular Tumour: tumour core, tumour/normal cells ratio is approximately 100–500/1; Perinecrotic Zone: boundary of tumour cells closely around necrotic areas in tumour core; Pseudopalisading Cells around Necrosis: characteristic rows of lined-up, aggregated cells surrounding necrotic areas in tumour core; Hyperplastic Blood Vessels in Cellular Tumour: aggregated blood vessels with thickened walls, in tumour core; Microvascular Proliferation: glomerulus-like conformation of a couple of blood vessels that share vessel wall, inside the tumour core. All images are credited to Allen Institute. (**a**), (**b**) and (**e**) are available on the Technical White Paper: Overview – 2015, accessible in glioblastoma.alleninstitute.org; (**c**) and (**d**) are available at http://glioblastoma.alleninstitute.org/ish/specimen/show/706783?gene=5127. Image credit: Allen Institute.

**Figure 2:**
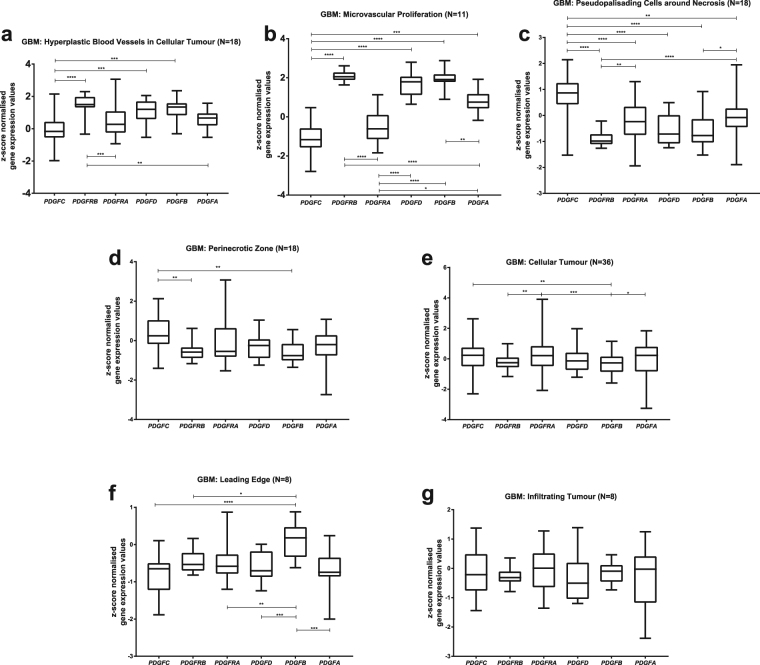
PDGF family expression patterns on each GBM histological region reveal intratumoral heterogeneity. *PDGF* system expression was evaluated in GBM regions of hyperplastic blood vessels in cellular tumour (**a**); microvascular proliferation (**b**); pseudopalisading cells around necrosis (**c**); perinecrotic zone (**d**); cellular tumour (**e**); leading edge (**f**), and infiltrating tumour (**g**).*p < 0.05; **p < 0.01; ***p < 0.001; ****p < 0.0001.

We analysed gene expression of the *PDGF* system (*PDGFA, PDGFB, PDGFC, PDGFD, PDGFRA, PDGFRB*) in 36 GBMs studied on Ivy Glioblastoma Atlas Project. Histological slices from tumour blocks (Fig. 1a–c) were annotated with molecular markers of seven laser-microdissected GBM regions (Fig. 1d): leading edge, infiltrating tumour, cellular tumour, perinecrotic zone, pseudopalisading cells around necrosis, hyperplastic blood vessels and microvascular proliferation (Fig. 1e). In hyperplastic blood vessels (Fig. 2a), *PDGFRB* shows high expression levels and *PDGFC* is less expressed than most of the other subunits, whereas the remaining subunits bears similar expression levels. Interestingly, in microvascular proliferation region (Fig. 2b), *PDGFRB* and *PDGFC* are also respectively more and less expressed in comparison to the other members of the family. In addition, *PDGFRA* bears lower expression values than all other subunits but *PDGFC*. The pseudopalisading cells (Fig. 2c), on the other hand, presents an inverse pattern: *PDGFC* is the most expressed *PDGF* subunit, *PDGFRB* has low expression values, and the remaining subunits are similarly expressed. *PDGFB* and *PDGFRB* are less expressed relatively to *PDGFC* in perinecrotic zone (Fig. 2d), and also in comparison to *PDGFRA* in cellular tumour block (Fig. 2e). As to the leading edge (Fig. 2f), *PDGFB* stands out as the most expressed *PDGF* subunit in the region. Contrastingly, in the area of infiltrating tumour (Fig. 2g), all subunits are expressed uniformly. Hence, GBM tumour is composed by heterogeneous regions, each one bearing a different *PDGF* expression pattern.

### PDGF subunits show differential expression along the GBM tumour

**Figure 3:**
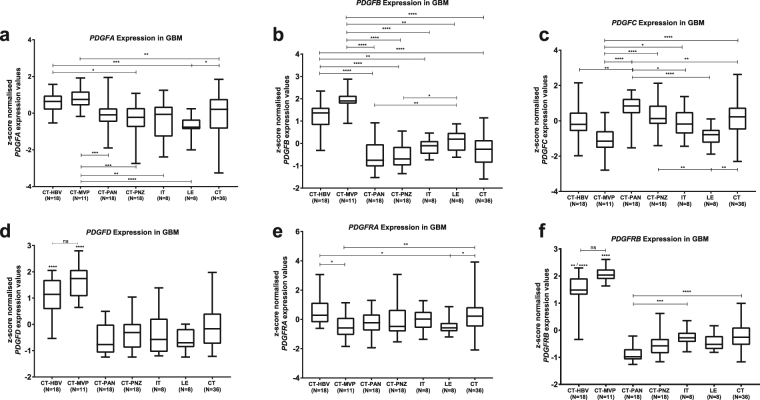
PDGF genes are differentially expressed over different histological regions of a GBM. Expression pattern along distinct areas of a GBM was assessed for each *PDGF* system component: *PDGFA* (**a**); *PDGFB* (**b**); *PDGFC* (**c**); *PDGFD* (**d**); *PDGFRA* (**e**); *PDGFRB* (**f**). CT-HBV: Hyperplastic Blood Vessels in Cellular Tumour; CT-MVP: Microvascular Proliferation; CT-PAN: Pseudopalisading Cells around Necrosis; IT: Infiltrating Tumour; LE: Leading Edge; CT: Cellular Tumour. *p < 0.05; **p < 0.01; ***p < 0.001; ****p < 0.0001.

Next, we analysed the distribution of each *PDGF* subunit along the GBM areas, by means of expression values. Both *PDGFA* (Fig. 3a) and *PDGFB* (Fig. 3b) subunits appear to be most expressed on microvascular proliferation, and expression levels in hyperplastic blood vessels are higher than on most of the remaining GBM regions. Differently, *PDGFC* (Fig. 3c) is less expressed in microvascular proliferation in comparison to all other GBM regions aside from hyperplastic blood vessel and leading edge, and similarly to *PDGFA* (Fig. 3a) and *PDGFRA* (Fig. 3e), it bears a preferential expression in cellular tumour bulk over leading edge. As to *PDGFD* (Fig. 3d) and *PDGFRB* (Fig. 3f), expression is the highest in areas of angiogenic alterations. Contrastingly, *PDGFRA* (Fig. 3e) is not uniformly distributed among vascular regions, being more expressed in hyperplastic blood vessels than on microvascular proliferation. Additionally, Allen Brain Institute has made available a set of *in situ* hybridization images of PDGF subunits, for eight GBMs. Although, at visual analysis, each subunit presents a particular expression pattern, comparison to the correspondents annotated histological sections does not reveal a clear correlation between gene expression and GBM region (see Supplementary Fig. S1). Altogether, these results characterise the heterogenic distribution of the *PDGF* family over GBM blocks, with the preferential expression of most of the subunits on areas of hyperplastic blood vessels and microvascular proliferation. However, such differential expression may not be appreciated in limited samples, with subjective methods of analysis.

### *PDGF* family is also heterogeneously expressed over the different locations of GBM in the brain

**Figure 4:**
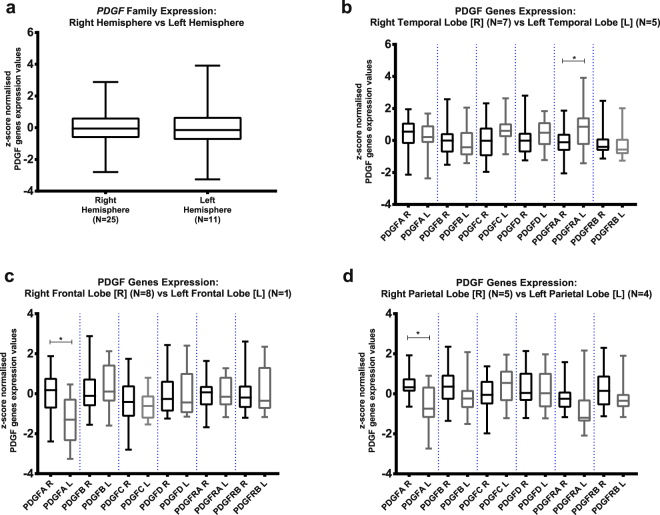
Analysis of PDGF genes expression regarding GBM location in the brain: Comparison was made on *PDGF* family expression between tumours located at left and at right hemispheres (**a**); PDGF subunits expression was studied on GBMs at temporal lobe (**b**), frontal lobe (**c**) and parietal lobe (**d**). *p < 0.05; **p < 0.01; ***p < 0.001; ****p < 0.0001.

Following the analysis of *PDGF* expression in histological GBM regions, we studied *PDGF* system expression with regard to the GBM-affected part of the brain. Although *PDGF* family seems to have overall similar expression levels between GBMs located in the right hemisphere and in the left one (Fig. 4a), analysis of *PDGF* expression per lobes of the brain reveals a more heterogeneous panel. *PDGFRA* is more expressed in GBMs of the left temporal lobe than in those of the right one (Fig. 4b), whereas *PDGFA* expression is higher when the tumour is set on right frontal (Fig. 4c) and parietal (Fig. 4d) lobes as opposed to the left correspondents. Therefore, GBM appears to present intertumoral heterogeneity as to *PDGF* family, of which the expression pattern changes depending on the location of the tumour in the brain.

### *PDGF* expression is a potential bad prognosis marker

**Figure 5:**
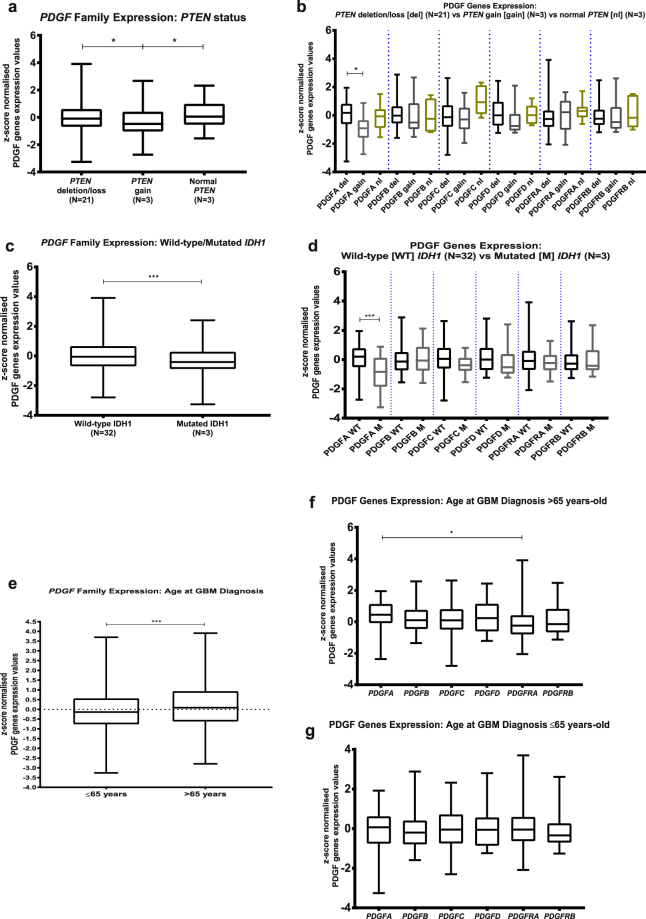
PDGF expression correlates with prognostic factors of GBM. *PDGF* system expression was analysed according to prognostic factors of Phosphatase and Tensin Homolog (*PTEN*) deletion (**a**,**b**); Isocitrate Dehydrogenase 1 (*IDH1)* mutation (**c**,**d**), and age at diagnosis of GBM (**e**,**f**,**g**). *p < 0.05; **p < 0.01; ***p < 0.001; ****p < 0.0001.

Thanks to the extensive clinical and genomic data on the donors of the GBM blocks herein studied, available on Ivy GAP Clinical and Genomic Database, we were able to analyse PDGF genes expression on the GBM as to the presence of molecular or clinical markers that determine a bad prognosis. No significant correlation was found between *PDGF* expression and Epidermal Growth Factor Receptor genetic and mutational status, Karnofsky Performance Status score and methylation status of O6-methylguanine-DNA-methyltransferase promoter (see Supplementary Fig. S2). On the other hand, GBMs that present *PTEN* deletion, considered to be a bad prognosis marker^13,15–17^, have higher *PDGF* family expression levels than the tumours with *PTEN* gain, which correlates with a better prognosis (Fig. 5a); analysis of each PDGF subunit separately reveals that *PDGFA* is differentially expressed between these two types of GBM (Fig. 5b). Likewise, GBMs with wild-type *IDH1*, a bad prognosis determinant^10,11,14^, show significantly (p value 0.0004) higher overall *PDGF* family expression over the ones bearing the mutated version of this gene (Fig. 5c), and subunit-by-subunit analysis shows that *PDGFA*, in particular, follows this pattern (Fig. 5d). As to the age at the time of GBM diagnosis, those with more than 65 years-old are reported to have poorer prognosis^12^, and among the subjects herein investigated, tumours from patients in this age group are characterised by greater *PDGF* family expression levels (Fig. 5e) and increased *PDGFA* expression over *PDGFRA* (Fig. 5f). Contrastingly, GBMs of patients diagnosed before or at 65 years-old present lower *PDGF* family expression (Fig. 5e) and uniform expression of PDGF subunits (Fig. 5g). Thus, the expression levels of *PDGF* family, especially the subunit *PDGFA*, correlate with the presence of poor prognostic factors, which suggests these genes may be viewed as prognostic markers themselves.

## Discussion

This study presents a broad perspective on the inter- and intratumoral heterogeneity of the *PDGF* family expression in GBMs, along with the potential prognostic significance of these genes expression, from analysis of the comprehensive database of the Ivy Glioblastoma Atlas Project – Allen Institute for Brain Science.

The “multiforme” designation of this high-grade tumour is much accurate on making explicit its wide heterogeneous milieu, which has been studied by different groups at cytogenic, transcriptional, mutational, epigenetic and proteomic levels, being regarded as key point to understanding differential responses to treatment as well as therapeutic resistance so common to GBM patients^18–25^. The level of heterogeneity on a GBM is linked to poor survival^26^ and seems to represent a spatially and temporally dynamic multistep-process that involves genetic instability and clonal proliferation, equipping the tumour cells with aggressiveness and resourcefulness to survival and growth^27,28^.

GBM has been reported to bear copy number aberrations and overexpression of receptor tyrosine kinases, especially PDGFRA, EGFR (Epidermal Growth Factor Receptor) and MET proto-oncogene, in a significantly non-homogeneous presentation, with subpopulations of the same tumour presenting each genetic alteration in a mutually exclusive, mosaic-like way^26,29–32^. Interestingly, PDGF genes have been shown to present differential expression between glial cells of tumour mass and endothelial cells of angiogenic alterations in the tumour, with description of autocrine and paracrine loops involving specific PDGF receptors and ligands, which are thought to perpetuate cell proliferation and tumour growth in the different areas^33–35^. Moreover, PDGFRβ has been described to be the type of PDGF receptor preferentially expressed in GBM stem-cells and to promote their self-renewal and invasion, which is likely correlated with tumour recurrence and resistance to therapeutics^36^.

However, at the moment of this publication, the authors had not found any report regarding analysis of the entire *PDGF* family expression among different histologically-defined GBM regions, as performed here. Likewise, intertumoral heterogeneity as to PDGF genes expression had not been studied by means of comparison of tumours located in different brain lobes. Thus, the present work reaffirms the emblematic feature of heterogeneity on GBM, characterising the distribution and pattern of expression of *PDGF*, which is associated not only to normal neurogenesis but also to glial tumour initiation and progression^37–39^.

Overall and progression-free survival periods are usually very short after GBM clinical presentation, as illustrated by the subjects studied here, the majority of which died less than one year after GBM diagnosis. Patients with post-diagnosis survival period of more than 2.5 years are classified as long-term survivors^40^. Because of this tragic prognosis, multiple markers have been investigated with the aim of stratifying patients accordingly to disease severity and therapeutic options. In this context, age at GBM diagnosis, *IDH1* mutations and *PTEN* deletions have been described as independent prognostic factors^10–15,17,41^. The herein shown correlation between PDGF genes expression and the clinical and genomic prognostic factors aforementioned suggests that those genes should be further considered as additional prognosis markers that may aid clinical management of GBM patients.

Growing evidence as to the significance of understanding inter- and intratumoral heterogeneity, as well as prognosis biomarkers, for the stratification of GBM patients and decisions over therapeutic strategies for them has been taken into consideration in the clinical setting. The characterisation of tumour subtypes with specific transcriptional profiles described by groups such as Verhaak *et al*., 2010^42^ has been applied in the interpretation of GBM heterogeneity and prognosis in recent studies and has motivated the newest revision of World Health Organisation classification of central nervous system tumours towards a molecular-based analysis of each tumour together with the traditional histopathological appraisal^43,44^. A more precise classification of GBM, taking into account its heterogeneity, will certainly have a positive effect on implementation of targeted therapy tailored for each patient, which greatly increases the chances of changing the current poor prognosis paradigm and reaching curative treatments in the near future.

In brief, the present study contributes to the characterisation of GBM heterogeneity as it reveals that PDGF genes show specific expression patterns through different regions of a GBM as well as differential expression accordingly to the location of the tumour in the brain. Of note, the *PDGF* family can also be linked to prognostic factors of the GBM. Taken together, these results should contribute to the realization of personalised medicine towards the development of successful therapeutics against this so common and so devastating tumour.

## Methods

### Compilation from Allen Institute resources

Gene expression, clinical and genomic data on 36 primary GBMs and their donors were compiled from two different platforms of Ivy Glioblastoma Atlas Project [© 2015 Allen Institute for Brain Science. Ivy Glioblastoma Atlas Project. Available from: glioblastoma.alleninstitute.org]. Z-score normalised expression values of PDGF subunits were downloaded from the Anatomic Structures RNA-Sequencing data set of the online Allen Brain Atlas [Available in: glioblastoma.alleninstitute.org/rnaseq/search/index.html, last access in May 2016]. Gene expression data available in this open access atlas was obtained by RNA sequencing technique, applied to the seven GBM histological structures that were isolated by laser micro-dissection in each histological section of tumour blocks: Leading Edge, Infiltrating Tumour, Cellular Tumour, Perinecrotic Zone, Pseudopalisading Cells around Necrosis, Hyperplastic Blood Vessels in Cellular Tumour and Microvascular Proliferation. All values are described to be processed through post-hoc data normalisation followed by TbT normalisation. More detailed description on the methodology of gene expression data production is available on the Technical White Paper: Overview – 2015, accessible in glioblastoma.alleninstitute.org. Additionally, extensive clinical and genomic data of the patients recruited for the study are available in the online Ivy GAP Clinical and Genomic Database [Available in: ivygap.swedish.org/home, last access in May 2016] and were gathered and tabulated.

### Analysis of compiled data

Gene expression values of PDGF subunits (*PDGFA, PDGFB, PDGFC, PDGFD, PDGFRA, PDGFRB*) were analysed with regard to variables such as GBM histological structure, clinical parameters and genomic data. GraphPad Prism version 7.00 for Windows, GraphPad Software, La Jolla California USA (www.graphpad.com) was used for graphical representation and statistical analysis. Differences between two sample groups were assessed by Mann-Whitney test, whereas multiple comparisons were evaluated by Kruskal-Wallis test followed by Dunn’s Multiple Comparisons test. Values were assessed as medians and correlations were considered statistically significant if p value <0.05.

### Data availability statement

The datasets analysed during the current study are from the © 2015 Allen Institute for Brain Science - Ivy Glioblastoma Atlas Project, available in: glioblastoma.alleninstitute.org and ivygap.swedish.org/home.

## Electronic supplementary material

Supplementary Information
