# Supplementary material for: PDGF Family Expression in Glioblastoma Multiforme: Data Compilation from Ivy Glioblastoma Atlas Project Database
Source: Sci Rep. 2017 Nov 10;7:15271. doi: 10.1038/s41598-017-15045-w (PMC5681588; doi:10.1038/s41598-017-15045-w)
Supplement: Supplementary file 1 — Supplementary Information [file 41598_2017_15045_MOESM1_ESM.pdf]

PDGF Family Expression in Glioblastoma Multiforme: Data Compilation from Ivy Glioblastoma Atlas Project Database

Isabella Gomes Cantanhede, João Ricardo Mendes de Oliveira

Supplementary Table S1: Clinical and genomic details of all donors of the tumors analyzed in this study. Data compiled from the Ivy GAP Clinical and Genomic Database [ivygap.swedish.org/home].

Patient ID	W1-1-1	W2-1-1	W3-1-1
**Patient Information**			
Gender	Male	Female	Female
Age at Initial Diagnosis	67	65	66
Weight	106.5 kg	62.9 kg	50.5 kg
Height	180 cm	160 cm	158.8 cm
KPS	100	90	100
Mini Mental Status Exam Score			
History of Seizures Since Last Visit	Yes	Yes	No
Neurologic Deficit	No	No	Yes. Left
Presentation Symptoms	Six-week history of mental status changes	Seizure	Slurred speech, slight left facial droop, decreased grip strength to left hand
Time to Progression	95 days	908 days	328 days
Overall Survival	106 days	1096 days	982 days
Time to Last Follow-up	NA	NA	NA
Cause Of Death	Tumor progression	Tumor progression	Tumor progression
**Tumor Information**			
**Tumor 1**			
Date (months from initial diagnosis)	0	0	0
Diagnosis	Astrocytic tumor	Astrocytic tumor	Astrocytic tumor
Grade	Glioblastoma WHO grade IV	Glioblastoma WHO grade IV	Glioblastoma WHO grade IV
Location	Right Temporal	Right Frontal	Right Frontal
**Tumor 2**			
Date (months from initial diagnosis)	0		18
Diagnosis	Astrocytic tumor		Astrocytic tumor

Grade	Glioblastoma WHO grade IV		Glioblastoma WHO grade IV
Location	Right Frontal		Right Temporal
**Medications**			
**Medication 1**			
Category	Anti-epileptic	Anti-epileptic	Steroids
Drug Name	Fosphenytoin	Levetiracetam	Dexamethasone
Start (months from initial diagnosis)	-1	-1	-1
Total Daily Dosage	300-400 mg	1000 mg	1-12mg
End (months from initial diagnosis)	0	36	32
**Medication 2**			
Category	Anti-epileptic	Steroids	Anti-epileptic
Drug Name	Levetiracetam	Dexamethasone	Levetiracetam
Start (months from initial diagnosis)	0	-1	-1
Total Daily Dosage	3000 mg	1-2mg	500-2000mg
End (months from initial diagnosis)	3	3	32
**Medication 3**			
Category	Steroids	Cholesterol lowering	Antihypertensive
Drug Name	Dexamethasone	Gemfibrozil	Digoxin
Start (months from initial diagnosis)	0	-1	0
Total Daily Dosage	2-16 mg	1200mg	125mg
End (months from initial diagnosis)	3	5	14
**Medication 4**			
Category	Anti-epileptic		Antihypertensive
Drug Name	Fosphenytoin		Diltiazem
Start (months from initial diagnosis)	0		0
Total Daily Dosage	1500 mg ONE TIME		60mg
End (months from initial diagnosis)	0		3
**Medication 5**			
Category			Cardiotonic
Drug Name			Enalapril
Start (months from initial diagnosis)			0
Total Daily Dosage			10mg
End (months from initial diagnosis)			26
**Medication 6**			
Category			Anti-depressant

Drug Name			Quetiapine
Start (months from initial diagnosis)			2
Total Daily Dosage			25mg
End (months from initial diagnosis)			4
**Medication 7**			
Category			Antihypertensive
Drug Name			Amlodipine
Start (months from initial diagnosis)			13
Total Daily Dosage			10mg
End (months from initial diagnosis)			24
**Medication 8**			
Category			Antihypertensive
Drug Name			Amlodipine
Start (months from initial diagnosis)			18
Total Daily Dosage			10mg
End (months from initial diagnosis)			18
**Medication 9**			
Category			Antihypertensive
Drug Name			Verapamil
Start (months from initial diagnosis)			22
Total Daily Dosage			180-240mg
End (months from initial diagnosis)			32
**Medication 10**			
Category			Antihypertensive
Drug Name			Verapamil
Start (months from initial diagnosis)			26
Total Daily Dosage			80mg (one time)
End (months from initial diagnosis)			26
**Surgery**			
**Surgery 1**			
Date of Surgery (months from initial diagnosis)	0	0	0
Gliadel	No	No	No
Awake mapping	No	No	Yes
Stealth scan	Yes	Yes	Yes

**Surgery 2**			
Date of Surgery (months from initial diagnosis)			18
Gliadel			Yes
Awake mapping			Yes
Stealth scan			Yes
**Tissue Diagnosis**			
**Tissue 1**			
Serial Number	W1-1-1, SN154-1	W2-1-1, SN156	W3-1-1, SN159
Diagnosis	Glioblastoma WHO grade IV	Glioblastoma WHO grade IV	Glioblastoma WHO grade IV
Location	Right Temporal	Right Frontal	Right Frontal
Extent	Complete resection	Complete resection	Complete resection
MRI confirmation	Yes	Yes	Yes
**Tissue 2**			
Serial Number	W1-1-2, SN154-5		SN236, W3-2-1
Diagnosis	Glioblastoma WHO grade IV		Glioblastoma WHO grade IV
Location	Right Frontal		Right Temporal
Extent	Complete resection		Complete resection
MRI confirmation	Yes		Yes
**Chemotherapy**			
**Medication 1**			
Drug Name	Temozolomide	Temozolomide	Temozolomide
Start (months from initial diagnosis)	0	1	0
Dosage	140mg	330mg	105mg
End (months from initial diagnosis)	2	2	2
Description	Concurrent	Concurrent	Concurrent
**Medication 2**			
Drug Name		Temozolomide	Temozolomide
Start (months from initial diagnosis)		3	4
Dosage		250mg	215mg
End (months from initial diagnosis)		7	8

Description		maintenance 5x/mo.	Maintenance 5d/monthx8cycles
**Medication 3**			
Drug Name		Temozolomide	Temozolomide
Start (months from initial diagnosis)		9	8
Dosage		250mg	105mg
End (months from initial diagnosis)			11
Description		maintenance 5x/mo.	21 day cycles
**Medication 4**			
Drug Name			XL-184
Start (months from initial diagnosis)			11
Dosage			
End (months from initial diagnosis)			12
Description			
**Medication 5**			
Drug Name			Temozolomide
Start (months from initial diagnosis)			22
Dosage			100mg QD
End (months from initial diagnosis)			25
Description			21 day cycles
**Medication 6**			
Drug Name			Avastin
Start (months from initial diagnosis)			25
Dosage			475mg
End (months from initial diagnosis)			31
Description			
**Radiation Therapy**			
**Medication 1**			
Type	WBRT	IMRT	IMRT
Start (months from initial diagnosis)	0	1	0
Dosage	5000 cGy	4600 cGy	4500 cGy
End (months from initial diagnosis)	1	2	2
Description		Concurrent	

Fraction		23	25
**Medication 2**			
Type	WBRT	IMRT	Stereotactic
Start (months from initial diagnosis)	1	2	10
Dosage	800 cGy	1400 cGy	2647 cGy
End (months from initial diagnosis)	2	2	10
Description	Boost	Concurrent Boost	
Fraction		7	1
**Medication 3**			
Type			Stereotactic
Start (months from initial diagnosis)			20
Dosage			2000 cGy
End (months from initial diagnosis)			20
Description			
Fraction			5
**Pathology**			
**Surgery 1**			
Date Of Report (months from initial diagnosis)	0	0	0
**Report**			
Location	Tissue: W1-1-1, SN154-1	Tissue: W2-1-1, SN156	Tissue: W3-1-1, SN159
Histopathology	Glioblastoma, WHO grade IV View Slides: A B C	Glioblastoma, WHO grade IV View Slides: A B C	Glioblastoma, WHO grade IV View Slides: A B C
1p19q_deletion	negative	positive	Negative
EGFR	amplification	amplification	amplification
PTEN	deletion	loss	loss
MGMT PCR	unmethylated	methylated	unmethylated
MGMT IHC	40% View Slides: A	negative	30% View Slides: A
MGMT MS-MLPA:R1	unmethylated (0.15)	methylated (0.312906321)	unmethylated (0.253645289)
MGMT MS-MLPA:R2	unmethylated (0.1)	methylated (0.710814512)	unmethylated (0.047337683)
MGMT MS-MLPA:R3	unmethylated (0.07)	methylated (0.723082006)	unmethylated (0.039502756)
IDH1 mutation	wildtype	wildtype	wildtype

EGFR vIII	not detected	not detected	detected
**Surgery 2**			
Date Of Report (months from initial diagnosis)			18
**Report**			
Location			Tissue: SN236, W3-2-1
Histopathology			recurrent/residual glioblastoma, WHO grade IV
1p19q_deletion			
EGFR			
PTEN			
MGMT PCR			unmethylated
MGMT IHC			20%
MGMT MS-MLPA:R1			methylated (0.39)
MGMT MS-MLPA:R2			unmethylated (0.06)
MGMT MS-MLPA:R3			unmethylated (0.03)
IDH1 mutation			
EGFR vIII			detected
**Follow-up Clinic Visit**			
**Visit 1**			
Date of Visit (months from initial diagnosis)	*Patient has no follow-up clinic visits*	3	0
KPS		70 (?)	
Mini Mental Status Exam Score			27
History of seizures since last visit		No	No
Neurologic Deficit		No	No
Symptoms Notes		Poor concentration, slowed cognition, hallucinations, falls x3	No new problems
**Visit 2**			
Date of Visit (months from initial diagnosis)		5	2
KPS			90
Mini Mental Status Exam Score		16	
History of seizures since last visit		No	No

Neurologic Deficit		No	No
Symptoms Notes		cognitive slowing, likely due to bifrontal lobe dysfunction and side effects from temozolomide	Some persistent numbness in her left hand starting about two weeks after surgery, but this has been stable.
**Visit 3**			
Date of Visit (months from initial diagnosis)		7	4
KPS			90
Mini Mental Status Exam Score		21	30
History of seizures since last visit		No	No
Neurologic Deficit		No	No
Symptoms Notes		Decreased Motivation	The first cycle of adjuvant temozolomide chemotherapy was tolerated poorly with abdominal pain, fairly significant nausea, and general ill feeling.
**Visit 4**			
Date of Visit (months from initial diagnosis)		9	4
KPS			90
Mini Mental Status Exam Score		19	
History of seizures since last visit		No	Yes
Neurologic Deficit		No	No
Symptoms Notes		Occasional abnormal thoughts including hallucinations	Prominent nausea during cycle 1 of postradiation Temodar. Other than that, she is clinically stable
**Visit 5**			
Date of Visit (months from initial diagnosis)		11	6
KPS			100

Mini Mental Status Exam Score		21	29
History of seizures since last visit		No	No
Neurologic Deficit		No	No
Symptoms Notes		She has had some mild decrease in her endurance.	There have been no significant new problems.
**Visit 6**			
Date of Visit (months from initial diagnosis)		13	7
KPS			100
Mini Mental Status Exam Score		20	30
History of seizures since last visit		No	No
Neurologic Deficit		Yes. Left.	No
Symptoms Notes		Gradual deterioration in gait function and some very mild weakness in her left lower extremity. **OBS**: patient unable to complete MMSE	No significant new problems since last visit.
**Visit 7**			
Date of Visit (months from initial diagnosis)		15	7
KPS			100
Mini Mental Status Exam Score		19	30
History of seizures since last visit		No	No
Neurologic Deficit		No	No
Symptoms Notes		Gait has continued to be somewhat of a problem, but is fluctuating substantially where she has some very good days and some rather poor days.	No new problems to report.
**Visit 8**			
Date of Visit (months from initial diagnosis)		18	8
KPS			100

Mini Mental Status Exam Score		22	30
History of seizures since last visit		No	No
Neurologic Deficit		No	No
Symptoms Notes		Slight further deterioration in her walking.	No new problems to report.
**Visit 9**			
Date of Visit (months from initial diagnosis)		21	10
KPS			
Mini Mental Status Exam Score		17	30
History of seizures since last visit		No	No
Neurologic Deficit		No	No
Symptoms Notes		Comes in today with a new MRI. Neurologically she continues to have a slow gradual decline. She continues to be pleasantly confused. She is having increase lower extremity weakness and some days is unable to walk. She denies any headaches or seizures. She denies any pain. She does have some fatigue and this is when her walking deteriorates. All other systems are negative except as noted above.	She has no new problems to report.
**Visit 10**			
Date of Visit (months from initial diagnosis)		29	11
KPS			90
Mini Mental Status Exam Score		15	
History of seizures since last visit		No	No

Neurologic Deficit		No	No
Symptoms Notes		Gradual deterioration in neurologic function, most notably in terms of motivation and gait; gradual progression of bilateral frontal lobe dysfunction.	Her energy is improving since decline following radiation 4 months ago. Continues to be quite active.
**Visit 11**			
Date of Visit (months from initial diagnosis)			11
KPS			90
Mini Mental Status Exam Score			
History of seizures since last visit			No
Neurologic Deficit			No
Symptoms Notes			She is having some nausea and anorexia as the side effect of the XL184.
**Visit 12**			
Date of Visit (months from initial diagnosis)			12
KPS			90
Mini Mental Status Exam Score			30
History of seizures since last visit			No
Neurologic Deficit			No
Symptoms Notes			Over the past week off medication she has had significant improvement in nausea, vomiting, and diarrhea. Her headache has improved.
**Visit 13**			
Date of Visit (months from initial diagnosis)			15
KPS			90

Mini Mental Status Exam Score			
History of seizures since last visit			No
Neurologic Deficit			No
Symptoms Notes			She had come off the investigational XL 184 agent because of side effects. She is now been on observation and is feeling well.
**Visit 14**			
Date of Visit (months from initial diagnosis)			17
KPS			
Mini Mental Status Exam Score			30
History of seizures since last visit			Yes
Neurologic Deficit			No
Symptoms Notes			She is now in the observation phase and is doing well with no headaches or seizures.
**Visit 15**			
Date of Visit (months from initial diagnosis)			17
KPS			
Mini Mental Status Exam Score			30
History of seizures since last visit			No
Neurologic Deficit			No
Symptoms Notes			She has no new problems to report.
**Visit 16**			
Date of Visit (months from initial diagnosis)			18
KPS			90
Mini Mental Status Exam Score			
History of seizures since last visit			No

Neurologic Deficit			No
Symptoms Notes			She has been doing well since I last saw her with no significant headaches or seizures.
**Visit 17**			
Date of Visit (months from initial diagnosis)			18
KPS			90
Mini Mental Status Exam Score			
History of seizures since last visit			No
Neurologic Deficit			No
Symptoms Notes			She has had no new problems.
**Visit 18**			
Date of Visit (months from initial diagnosis)			20
KPS			90
Mini Mental Status Exam Score			
History of seizures since last visit			No
Neurologic Deficit			No
Symptoms Notes			She has been doing well with no significant headaches or seizures.
**Visit 19**			
Date of Visit (months from initial diagnosis)			22
KPS			90
Mini Mental Status Exam Score			
History of seizures since last visit			No
Neurologic Deficit			No

Symptoms Notes			Stable disease after CyberKnife treatment.
**Visit 20**			
Date of Visit (months from initial diagnosis)			23
KPS			90
Mini Mental Status Exam Score			
History of seizures since last visit			No
Neurologic Deficit			No
Symptoms Notes			Slight increase in thickness of enhancement along the anterior aspect of the resection cavity; however, overall there is no significant change in the study.
**Visit 21**			
Date of Visit (months from initial diagnosis)			24
KPS			90
Mini Mental Status Exam Score			
History of seizures since last visit			No
Neurologic Deficit			No
Symptoms Notes			Slight interval increase in the thickness of contrast enhancement around the margin of the resection cavity; minor, not conclusive of progressive disease.
**Visit 22**			
Date of Visit (months from initial diagnosis)			25
KPS			80
Mini Mental Status Exam Score			
History of seizures since last visit			No

Neurologic Deficit			No
Symptoms Notes			Tumor is clearly showing progressive disease, halting TMZ and starting Avastin.
**Visit 23**			
Date of Visit (months from initial diagnosis)			26
KPS			70
Mini Mental Status Exam Score			
History of seizures since last visit			No
Neurologic Deficit			Yes. Left side weakness.
Symptoms Notes			Sensory problems in the left side making it difficult for her to use her left arm or to walk without assistance.
**Visit 24**			
Date of Visit (months from initial diagnosis)			27
KPS			70
Mini Mental Status Exam Score			
History of seizures since last visit			No
Neurologic Deficit			No
Symptoms Notes			Recently admitted to the hospital because of hypertension and her blood pressures, now under control. No further episodes of sudden onset left-sided weakness, although still unable to detect sensation on the left side to light touch.
**Visit 25**			
Date of Visit (months from initial diagnosis)			27
KPS			70

Mini Mental Status Exam Score			
History of seizures since last visit			No
Neurologic Deficit			No
Symptoms Notes			Early followup; potential toxicity after starting Trileptal. Dizziness, confusion, aphasic. CT scan revealed no acute findings, Trileptal discontinued. **OBS**: ER visit.
**Visit 26**			
Date of Visit (months from initial diagnosis)			29
KPS			70
Mini Mental Status Exam Score			
History of seizures since last visit			No
Neurologic Deficit			No
Symptoms Notes			She has no new problems to report. She continues to tolerate the every 2-week Avastin treatments well.
**Visit 27**			
Date of Visit (months from initial diagnosis)			30
KPS			70
Mini Mental Status Exam Score			
History of seizures since last visit			No
Neurologic Deficit			No
Symptoms Notes			Some fluctuations in neurologic status, but overall has been doing well.
**Clinical Trials**			

1	//	//	Exelixis, Relapsed Astrocytic Tumors with XL184 Regimen, 10041
2			NovoCure
3			

Patient ID	W4-1-1	W5-1-1	W6-1-1
**Patient Information**			
Gender	Female	Female	Female
Age at Initial Diagnosis	51	65	73
Weight	86.183 kg	85.3 kg	55.067 kg
Height	163.56 cm	160 cm	162.6 cm
KPS	90 (?)	90 (?)	90 (?)
Mini Mental Status Exam Score	30		
History of Seizures Since Last Visit	No	No	No
Neurologic Deficit	Yes. Left.	Yes. Left facial droop and hand weakness.	Yes. Right
Presentation Symptoms	Left-sided weakness 4/5, headache.	Weakness of left upper extremity, left facial droop.	Difficulty with reading comprehension and writing. Notes: visual field cut.
Time to Progression	102 days	115 days	107 days
Overall Survival	540 days	NA	647 days
Time to Last Follow-up	NA	752 days	NA
Cause Of Death	Tumor progression	//	Tumor progression
**Tumor Information**			
**Tumor 1**			
Date (months from initial diagnosis)	0	0	0
Diagnosis	Astrocytic tumor	Astrocytic tumor	Astrocytic tumor
Grade	Glioblastoma WHO grade IV	Glioblastoma WHO grade IV	Glioblastoma WHO grade IV
Location	Right Frontal	Right Frontal	Left Occipital
**Tumor 2**			
Date (months from initial diagnosis)	3		
Diagnosis	Astrocytic tumor		
Grade	Glioblastoma WHO grade IV		

Location	Right Frontal		
**Medications**			
**Medication 1**			
Category	Steroids	Anti-epileptic	Anti-epileptic
Drug Name	Dexamethasone	Levetiracetam	Phenytoin
Start (months from initial diagnosis)	-1	0	0
Total Daily Dosage	2-16mg	1500mg	200mg
End (months from initial diagnosis)	16	4	3
**Medication 2**			
Category	Anti-epileptic	Steroids	Steroids
Drug Name	Fosphenytoin	Dexamethasone	Dexamethasone
Start (months from initial diagnosis)	-1	0	0
Total Daily Dosage	100-300mg	2-16mg	12-16mg
End (months from initial diagnosis)	0	1	3
**Medication 3**			
Category	Anti-epileptic	Anti-depressant	Anti-epileptic
Drug Name	Phenytoin	Citalopram	Fosphenytoin
Start (months from initial diagnosis)	-1	0	0
Total Daily Dosage	100mg	10mg	300mg
End (months from initial diagnosis)	2	0	0
**Medication 4**			
Category	Steroids	Anti-epileptic	Anti-epileptic
Drug Name	Levetiracetam	Divalproex	Levetiracetam
Start (months from initial diagnosis)	-1	4	9
Total Daily Dosage	1000-2000mg	500mg	1000mg
End (months from initial diagnosis)	16	7	18
**Medication 5**			
Category	Anti-depressant		Antihypertensive
Drug Name	Citalopram		Amlodipine
Start (months from initial diagnosis)	-1		12
Total Daily Dosage	10-20mg		5mg
End (months from initial diagnosis)	17		16
**Medication 6**			
Category	Steroids		Anti-depressant
Drug Name	Prednisone		Xanax

Start (months from initial diagnosis)	8		14
Total Daily Dosage	5mg		
End (months from initial diagnosis)	11		18
**Medication 7**			
Category	Antihypertensive		
Drug Name	Losartan		
Start (months from initial diagnosis)	9		
Total Daily Dosage	25mg		
End (months from initial diagnosis)	17		
**Medication 8**			
Category	Steroids		
Drug Name	Prednisone		
Start (months from initial diagnosis)	11		
Total Daily Dosage	12.5-20mg		
End (months from initial diagnosis)	17		
**Surgery**			
**Surgery 1**			
Date of Surgery (months from initial diagnosis)	0	0	0
Gliadel	No	No	No
Awake mapping	Yes	Yes	No
Stealth scan	Yes	Yes	Yes
**Surgery 2**			
Date of Surgery (months from initial diagnosis)	3		
Gliadel	Yes		
Awake mapping	No		
Stealth scan	Yes		
**Tissue Diagnosis**			
**Tissue 1**			
Serial Number	W4-1-1, SN160	W5-1-1, SN161	W6-1-1, SN162
Diagnosis	Glioblastoma WHO grade IV	Glioblastoma WHO grade IV	Glioblastoma WHO grade IV
Location	Right Frontal	Right Frontal	Left Occipital
Extent	Complete resection	Complete resection	Complete resection

MRI confirmation	Yes	Yes	Yes
**Tissue 2**			
Serial Number	W4-2-1, SN174		
Diagnosis	Glioblastoma WHO grade IV		
Location	Right Frontal		
Extent	Complete resection		
MRI confirmation	Yes		
Notes	may not be truly recurrent tumor		
**Chemotherapy**			
**Medication 1**			
Drug Name	Temozolomide	Temozolomide	Temozolomide
Start (months from initial diagnosis)	0	1	1
Dosage	140mg/day	140mg	120mg/day
End (months from initial diagnosis)	2	2	2
Description	concurrent	Concurrent	Concurrent
**Medication 2**			
Drug Name	Temozolomide	Temozolomide	Temozolomide
Start (months from initial diagnosis)	2	3	3
Dosage	140mg/day	180mg	200mg/day
End (months from initial diagnosis)	3	4	5
Description	maintenance	Maintenance 5days/month	Maintenance 5x/mo
**Medication 3**			
Drug Name	Temozolomide	Temozolomide	Avastin
Start (months from initial diagnosis)	4	4	5
Dosage	100mg/day	380mg	
End (months from initial diagnosis)		7	
Description	long-term	Maintenance 5days/month	1x/2wks
**Medication 4**			
Drug Name	Avastin	XL-184	
Start (months from initial diagnosis)	7	7	

Dosage	875mg	125mg	
End (months from initial diagnosis)	13	8	
Description		Clinical Trial	
**Medication 5**			
Drug Name		XL-184	
Start (months from initial diagnosis)		8	
Dosage		75mg	
End (months from initial diagnosis)		15	
Description		Clinical Trial	
**Medication 6**			
Drug Name		XL-184	
Start (months from initial diagnosis)		15	
Dosage		50mg	
End (months from initial diagnosis)		33	
Description		Clinical Trial	
**Medication 7**			
Drug Name		Avastin	
Start (months from initial diagnosis)		27	
Dosage			
End (months from initial diagnosis)			
Description		1x/2wks	
**Radiation Therapy**			
**Medication 1**			
Type	IMRT	IMRT	IMRT
Start (months from initial diagnosis)	0	1	1
Dosage	4500 cGy	4500 cGy	5940 cGy
End (months from initial diagnosis)	2	2	2
Description		6 MV technique	Concurrent
Fraction	25	25	33
**Medication 2**			
Type	IMRT	IMRT	
Start (months from initial diagnosis)	0	2	
Dosage	1080 cGy	1440 cGy	
End (months from initial diagnosis)	2	2	

Description	Reduced Field Boost	Multiple Field Boost	
Fraction	6	8	
**Pathology**			
**Surgery 1**			
Date Of Report (months from initial diagnosis)	0	0	0
**Report**			
Location	Tissue: W4-1-1, SN160	Tissue: W5-1-1, SN161	Tissue: W6-1-1, SN162
Histopathology	Glioblastoma WHO grade IV	Glioblastoma, WHO grade IV	Glioblastoma WHO grade IV
1p19q_deletion	Normal	Normal	Normal
EGFR	gain	amplification	gain
PTEN	deletion	loss	Normal
MGMT PCR	unmethylated	unmethylated	unmethylated
MGMT IHC	80%	20% View Slides: A	90%
MGMT MS-MLPA:R1	unmethylated (0.252065773)	methylated (0.751090128)	unmethylated (0.193418734)
MGMT MS-MLPA:R2	unmethylated (0.037667297)	unmethylated (0)	unmethylated (0.047114023)
MGMT MS-MLPA:R3	unmethylated (0.010635661)	methylated (0.782615912)	unmethylated (0.019431304)
IDH1 mutation	wildtype	wildtype	wildtype
EGFR vIII	not detected	not detected	not detected
**Surgery 2**			
Date Of Report (months from initial diagnosis)	3		
**Report**			
Location	Tissue: W4-2-1, SN174		
Histopathology	Glioblastoma WHO grade IV		
1p19q_deletion			
EGFR			
PTEN			
MGMT PCR	unmethylated		
MGMT IHC	60%		

MGMT MS-MLPA:R1	methylated (0.42)		
MGMT MS-MLPA:R2	unmethylated (0.05)		
MGMT MS-MLPA:R3	unmethylated (0.03)		
IDH1 mutation			
EGFR vIII			
**Follow-up Clinic Visit**			
**Visit 1**			
Date of Visit (months from initial diagnosis)	0	2	0
KPS	90	90	90
Mini Mental Status Exam Score			
History of seizures since last visit	No	No	No
Neurologic Deficit	Yes. Left pronator drift.	Yes. Left hemiparesis.	Yes. Right homonymous hemianopia.
Symptoms Notes	Fatigued, high fever; patient is depressed.	Unscheduled visit because of vesicular rash.	Difficulty with right-sided vision.
**Visit 2**			
Date of Visit (months from initial diagnosis)	1	3	3
KPS	90	60	
Mini Mental Status Exam Score		28	27
History of seizures since last visit	No	Yes	No
Neurologic Deficit	No	Yes. Left hemiparesis.	Yes. Right homonymous hemianopia.
Symptoms Notes	Right sided facial swelling, presthesias, headache.	Gradual, steady improvement in left lower extremity strength. Left arm is still very weak and functionally useless.	Fatigue.
**Visit 3**			
Date of Visit (months from initial diagnosis)	2	4	5
KPS	90	70	70
Mini Mental Status Exam Score			17
History of seizures since last visit	No	No	No
Neurologic Deficit	No	Yes. Left hemiparesis.	Yes. Right homonynous hemianopia.

Symptoms Notes	Temodar radiation tolerated well. Decreased appetite, fatigue, night sweats.	Keppra discontinued due to rash, risk of seizure possibly increased.	n/a
**Visit 4**			
Date of Visit (months from initial diagnosis)	3	5	7
KPS	90	70	
Mini Mental Status Exam Score		29	25
History of seizures since last visit	No	Yes	No
Neurologic Deficit	No. Left weakness.	Yes. Left hemiparesis.	Yes. Right homonymous hemianopia.
Symptoms Notes	Temodar tolerated well. Energy is better, appetite is better. Sleeping better. Still weak on left side, right is better.	Functions improving gradually. Elevated LFTs, discontinue dapsone.	Deteriorating language, speech is returning to baseline.
**Visit 5**			
Date of Visit (months from initial diagnosis)	4	7	8
KPS	90		80
Mini Mental Status Exam Score		28	
History of seizures since last visit	No	No	No
Neurologic Deficit	No	Yes. Left hemiparesis.	Yes. Right homonymous hemianopia.
Symptoms Notes	The patient had a repeat biopsy.	Slightly better control of left leg.	Reading is still arduous, but spelling and writing are becoming better. Denies any other new neurological deficits.
**Visit 6**			
Date of Visit (months from initial diagnosis)	5	7	9
KPS	80	70	80
Mini Mental Status Exam Score			
History of seizures since last visit	No	No	Yes
Neurologic Deficit	Yes. Left sided weakness.	Yes. Left hemiparesis.	Yes. Right homonymous hemianopia.

Symptoms Notes	Weak left arm, grip poor, swollen. Very dry skin probably related to the Temodar. Decreased appetite, fatigue.	n/a	One month ago ER visit; patient reported to have numbness of the left arm and leg. Probable seizure.
**Visit 7**			
Date of Visit (months from initial diagnosis)	6	7	10
KPS	70	70	70
Mini Mental Status Exam Score			
History of seizures since last visit	No	No	No
Neurologic Deficit	Yes. Right hemiparesis.	Yes. Left hemiparesis.	Yes. Right homonymous hemianopia.
Symptoms Notes	Her functional status has remained the same. She has weakness of the left arm but this is possibly attibutable, in part, to a frozen shoulder. Decreased appetite, fatigue. Otherwise, she feels the same.		Keppra is giving patient some difficulty with fatigue and loss of appetite.
**Visit 8**			
Date of Visit (months from initial diagnosis)	6	8	11
KPS	80	70	70
Mini Mental Status Exam Score			
History of seizures since last visit	No	No	Yes
Neurologic Deficit	Yes. Left hemiparesis.	Yes. Left hemiparesis.	Yes. Right homonymous hemianopia.
Symptoms Notes	Weakness on upper extremeties.	Tolerating therapy without difficulties.	Difficulty with concentration. Probable previous seizure, which was demonstrated by some difficulty the right eye as well as also memory and speech. Resolved.
**Visit 9**			

Date of Visit (months from initial diagnosis)	8	12	11
KPS	80	70	70
Mini Mental Status Exam Score			
History of seizures since last visit	No	No	No
Neurologic Deficit	Yes. Left hemiparesis.	Yes. Left hemiparesis.	Yes. Right homonymous hemianopia.
Symptoms Notes	Left sided weakness; not fully functional; incontinence.	Diminution in taste, decreased appetite, and has had corresponding weight loss.	Some difficulty with thinking; relatively stable.
**Visit 10**			
Date of Visit (months from initial diagnosis)	10	15	12
KPS	70	70	70
Mini Mental Status Exam Score			
History of seizures since last visit	No	No.	Yes
Neurologic Deficit	Yes. Left hemiparesis.	Yes. Left hemiparesis.	Yes. Right homonymous hemianopia.
Symptoms Notes	Decreased appetite, fatigue.	n/a	Moderate seizure yesterday.
**Visit 11**			
Date of Visit (months from initial diagnosis)	11	15	13
KPS	80	70	60
Mini Mental Status Exam Score			
History of seizures since last visit	No	No	Yes
Neurologic Deficit	Yes. Left hemiparesis.	Yes. Left hemiparesis.	Yes. Right homonymous hemianopia.
Symptoms Notes	n/a	Recurrent episodes of diarrhea. Continues to have decreased appetite. Weight has dropped to a greater than 20% reduction of baseline weight at the initiation of the study.	Recent seizure problem 3 days ago. Patient is still having difficutly with vision and comprehension. Hand-eye coordination is somewhat worse than before.
**Visit 12**			
Date of Visit (months from initial diagnosis)	11	16	14

KPS	70	70	60
Mini Mental Status Exam Score			
History of seizures since last visit	No	No	Yes
Neurologic Deficit	Yes. Left hemiparesis.	Yes. Left hemiparesis.	Yes. Right homonymous hemianopia.
Symptoms Notes	Balance is weak; beginning to fall more frequently.	Weight stabilized.	Another small seizure of the right extremity; they are generally accompanied by difficulty with speech.
**Visit 13**			
Date of Visit (months from initial diagnosis)	12	17	16
KPS	60	70	50
Mini Mental Status Exam Score			
History of seizures since last visit	No	No	Yes
Neurologic Deficit	Yes. Left hemiparesis.	Yes. Left hemiparesis.	Yes. Right homonymous hemianopia.
Symptoms Notes	Occasional headaches in the supraorbital region. Falling more.	Denies any problems with rash, diarrhea is easily managed, her energy level is adequate and her appetite is improved. She does admit to challenges, however, maintaining weight.	Recurrent albeit minor seizures. One month ago patient had either a seizure or a fall with pain. Then 3 days later patient was admitted with mental status changes, probable dehydration. Patient may have had petit mal seizure yesterday. Weight loss, mild.
**Visit 14**			
Date of Visit (months from initial diagnosis)	12	18	16
KPS	60	70	60
Mini Mental Status Exam Score			
History of seizures since last visit	No	No	No
Neurologic Deficit	Yes. Left hemiparesis.	Yes. Left hemiparesis.	Yes. Right homonymous hemianopia.

Symptoms Notes	Remains weak.	She had two days of diarrhea last week. She admits to having a decreased appetite with weight loss, but in general, she has a good appetite. Her energy level is adequate and she has been able to maintain weight until this recent episode of diarrhea.	Difficulty with concentration. No more seizures or hospitalizations for UTI.
**Visit 15**			
Date of Visit (months from initial diagnosis)	13	21	17
KPS	50	70	60
Mini Mental Status Exam Score			
History of seizures since last visit	Yes	No	No
Neurologic Deficit	Yes. Left hemiparesis.	Yes. Left hemiparesis.	Yes. Right homonymous hemianopia.
Symptoms Notes	Decrease in balance ability to control the left leg. can no longer stand for longer than 15 minutes. drags left foot when walking. MRI reflected a probable previous stroke.	n/a	No changes.
**Visit 16**			
Date of Visit (months from initial diagnosis)	14	24	18
KPS	50	70	50
Mini Mental Status Exam Score			
History of seizures since last visit	No	Yes	No
Neurologic Deficit	Yes. Left hemiparesis.	Yes. Left hemiparesis.	Yes. Right homonymous hemianopia.
Symptoms Notes	Worsening ataxia; physical state remains the same.	Had partial seizure with postictal confusion a couple days ago, uneventful recovery.	No new neurological complaints.

**Visit 17**			
Date of Visit (months from initial diagnosis)	15	27	
KPS	50	60	
Mini Mental Status Exam Score			
History of seizures since last visit	Yes	Yes	
Neurologic Deficit	Yes. Left hemiplegia.	Yes. Left hemiparesis.	
Symptoms Notes	Had stroke on Avastin. Patient was also noted to have some hydrocephalus. Decreased appetite, fatigue.	Had another partial seizure about a month ago with postictal confusion, but reluctant to take anti convulsant medication.	
**Visit 18**			
Date of Visit (months from initial diagnosis)	15	30	
KPS	50	60	
Mini Mental Status Exam Score			
History of seizures since last visit	Yes	No	
Neurologic Deficit	Yes. Left hemiparesis.	Yes. Left hemiparesis.	
Symptoms Notes	Right legt somewhat compromised. Overall, patient feels about the same.	Weight loss, discontinue XL 184 for a week.	
**Visit 19**			
Date of Visit (months from initial diagnosis)		33	
KPS		50	
Mini Mental Status Exam Score			
History of seizures since last visit		No	
Neurologic Deficit		Yes. Left hemiparesis.	
Symptoms Notes		Failed first investigational therapy and now treated on second relapse with single agent Avastin.	

Clinical Trials			
1	//	Exelixis, Relapsed Astrocytic Tumors with XL184 Regimen, 10041	//
2		NovoCure	

Patient ID	W7-1-1	W8-1-1	W9-1-1
**Patient Information**			
Gender	Male	Female	Male
Age at Initial Diagnosis	61	50	51
Weight	79.833 kg	53.1 kg	100.245 kg
Height	182.61 cm	165.0 cm	173.72 cm
KPS	100 (?)	70 (?)	90 (?)
Mini Mental Status Exam Score	26	9	23
History of Seizures Since Last Visit	No	No	No
Neurologic Deficit	No	Yes. Right. Visual field cut, aphasia.	No
Presentation Symptoms	Progressive right-sided/pancranial headaches, nausea, emesis, sense of imbalance, dizziness.	Progressive speech disturbance, clumsiness with right hand.	Headaches, memory and balance problems
Time to Progression	366 days	29 days	109 days
Overall Survival	437 days	442 days	145 days
Time to Last Follow-up	NA	NA	NA
Cause Of Death	Tumor progression	Tumor progression	Tumor progression
**Tumor Information**			
**Tumor 1**			
Date (months from initial diagnosis)	0	0	0
Diagnosis	Astrocytic tumor	Astrocytic tumor	Astrocytic tumor
Grade	Glioblastoma WHO Grade IV	Glioblastoma WHO Grade IV	Giant Cell Glioblastoma WHO grade IV
Location	Right Temporal	Left Parietal	Right Frontal
**Tumor 2**			

Date (months from initial diagnosis)		0	0
Diagnosis		Astrocytic tumor	Astrocytic tumor
Grade		Glioblastoma WHO grade IV	Glioblastoma WHO grade IV
Location		Left Temporal	Left occipital, left parietal, left temporal
**Medications**			
**Medication 1**			
Category	Anti-epileptic	Anti-epileptic	Steroids
Drug Name	Levetiracetam	Phenytoin	Dexamethasone
Start (months from initial diagnosis)	-1	-1	-1
Total Daily Dosage	1000-2000mg	100-400mg	4-16mg
End (months from initial diagnosis)	12	14	4
**Medication 2**			
Category	Steroids	Steroids	Anti-epileptic
Drug Name	Dexamethasone	Dexamethasone	Fosphenytoin
Start (months from initial diagnosis)	-1	-1	0
Total Daily Dosage	8-24mg	4-40mg	300mg
End (months from initial diagnosis)	12	14	0
**Medication 3**			
Category	Cholesterol lowering		Anti-epileptic
Drug Name	Simvastatin		Phenytoin
Start (months from initial diagnosis)	-1		0
Total Daily Dosage	20mg		300mg
End (months from initial diagnosis)	0		0
**Medication 4**			
Category	Antihypertensive		Anti-epileptic
Drug Name	Olmesartan		Levetiracetam
Start (months from initial diagnosis)	0		0
Total Daily Dosage	12.5mg		1000mg
End (months from initial diagnosis)	12		4
**Medication 5**			
Category	Antihypertensive		Steroids
Drug Name	Lisinopril		Prednisone
Start (months from initial diagnosis)	12		3
Total Daily Dosage	20mg		50mg

End (months from initial diagnosis)	12		3
**Surgery**			
**Surgery 1**			
Date of Surgery (months from initial diagnosis)	0	0	0
Gliadel	No	No	No
Awake mapping	No	Yes	No
Stealth scan	Yes	Yes	Yes
**Tissue Diagnosis**			
**Tissue 1**			
Serial Number	W7-1-1, SN163	W8-1-1, SN175	W9-1-1, SN179-1
Diagnosis	Glioblastoma WHO grade IV	Glioblastoma WHO grade IV	Glioblastoma WHO grade IV
Location	Right Temporal	Left Parietal	Right Frontal
Extent	Subtotal resection 90%	Complete resection	Complete resection
MRI confirmation	Yes	Yes	Yes
Notes		Temporal not resected	Left hemisphere tumor not resected
**Tissue 2**			
Serial Number			SN179-2
Diagnosis			Normal
Location			Right Frontal
Extent			Complete resection
MRI confirmation			Yes
**Chemotherapy**			
**Medication 1**			
Drug Name	Temozolomide	Temozolomide	Temozolomide
Start (months from initial diagnosis)	0	0	1
Dosage	155mg QD	120mg	140mg TD
End (months from initial diagnosis)	2	2	2
Description	21 day cycles	Concurrent	Concurrent
**Medication 2**			
Drug Name	Avastin	Avastin	

Start (months from initial diagnosis)	12	0	
Dosage	10mg/kg	520mg	
End (months from initial diagnosis)		13	
Description	Q14D	twice monthly	
**Medication 3**			
Drug Name	Irinotecan	Temozolomide	
Start (months from initial diagnosis)	12	2	
Dosage	125mg/m2	225mg	
End (months from initial diagnosis)	13	2	
Description	4 out of 6 week cycles	daily oral	
**Medication 4**			
Drug Name		Temozolomide	
Start (months from initial diagnosis)		3	
Dosage		300mg	
End (months from initial diagnosis)		7	
Description		2-3 weeks oral	
**Medication 5**			
Drug Name		Etoposide	
Start (months from initial diagnosis)		7	
Dosage		50mg BID	
End (months from initial diagnosis)		11	
Description		21 day cycles	
**Medication 6**			
Drug Name		Lomustine	
Start (months from initial diagnosis)		10	
Dosage		180mg	
End (months from initial diagnosis)		10	
Description		One time administration	
**Medication 7**			
Drug Name		Lomustine	
Start (months from initial diagnosis)		11	

Dosage			
End (months from initial diagnosis)		12	
Description		daily oral	
**Medication 8**			
Drug Name		Carboplatin	
Start (months from initial diagnosis)		12	
Dosage		465mg	
End (months from initial diagnosis)		12	
Description		One time administration	
**Radiation Therapy**			
**Medication 1**			
Type	IMRT	IMRT	WBRT
Start (months from initial diagnosis)	0	0	1
Dosage	5940 cGy	4500 cGy	4500 cGy
End (months from initial diagnosis)	2	1	2
Description		Concurrent	
Fraction	33	25	25
**Medication 2**			
Type	Stereotactic	IMRT	WBRT
Start (months from initial diagnosis)	12	1	2
Dosage	38 Gy	1440 cGy	1400 cGy
End (months from initial diagnosis)	12	2	2
Description	Gamma Knife	Cone Down Boost	Boost
Fraction		8	8
**Pathology**			
**Surgery 1**			
Date Of Report (months from initial diagnosis)	0	0	0
**Report**			
Location	Tissue: W7-1-1, SN163	Tissue: W8-1-1, SN175	Tissue: W9-1-1, SN179-1
Histopathology	Glioblastoma WHO grade IV	Glioblastoma, WHO grade IV	Glioblastoma
1p19q_deletion	Normal	Normal	Normal
EGFR	gain	gain	gain

PTEN	loss	loss	loss
MGMT PCR	methylated	unmethylated	unmethylated
MGMT IHC	negative	30% View Slides: A	30%
MGMT MS-MLPA:R1	methylated (0.473459822)	methylated (1.024080679)	unmethylated (0.210228374)
MGMT MS-MLPA:R2	methylated (0.38712508)	unmethylated (0.047513901)	unmethylated (0.063881572)
MGMT MS-MLPA:R3	methylated (0.395032755)	unmethylated (0.048225987)	unmethylated (0)
IDH1 mutation	wildtype	wildtype	wildtype
EGFR vIII	not detected	not detected	not detected
**Surgery 2**			
Date Of Report (months from initial diagnosis)			
**Report**			(Still surgery 1)
Location			Tissue: SN179-2
Histopathology			
1p19q_deletion			
EGFR			
PTEN			
MGMT PCR			
MGMT IHC			
MGMT MS-MLPA:R1			unmethylated ()
MGMT MS-MLPA:R2			unmethylated ()
MGMT MS-MLPA:R3			unmethylated ()
IDH1 mutation			
EGFR vIII			
**Follow-up Clinic Visit**			
**Visit 1**			
Date of Visit (months from initial diagnosis)	0	-1	0
KPS	100	70	90
Mini Mental Status Exam Score		9	23
History of seizures since last visit	No	No	No
Neurologic Deficit	No	Yes. Right	No

Symptoms Notes	In the intervening time, he has done exceedingly well and notes no symptoms at the present time.	Progressive speech disturbance, clumsiness with right hand.	He has been doing well. No longer evidence of left hemiparesis.
**Visit 2**			
Date of Visit (months from initial diagnosis)	3	0	0
KPS	100		90
Mini Mental Status Exam Score		9	
History of seizures since last visit	No	No	No
Neurologic Deficit	No	No	No
Symptoms Notes	Occasional headaches. Speech has been good. No memory changes. Overall, doing great.	Hesitant spontaneous speech, mild difficulty following one-step motor commands, and moderate difficulty with more complex commands, mild anomia.	Appetite is good, he has gained weight. Denies any neurological symptoms, able to walk without balance issues.
**Visit 3**			
Date of Visit (months from initial diagnosis)	11	0	2
KPS	90		80
Mini Mental Status Exam Score		10	
History of seizures since last visit	No	No	No
Neurologic Deficit	No	No. Aphasia.	No
Symptoms Notes	The only new complaints of the vision changes, with diplopia and dizziness.	Some increase in headache, language difficulty, and right-sided weakness. Some clinical deterioration.	Occasional headache when waking up in morning. Appetite has been excellent. Denies any neurological changes.
**Visit 4**			
Date of Visit (months from initial diagnosis)	12	0	2
KPS			80
Mini Mental Status Exam Score	26	9	
History of seizures since last visit	No	No	No

Neurologic Deficit	No	Yes. Right-sided weakness, aphasia.	No
Symptoms Notes	Approximately one month ago, he began to complain of dizziness and some diplopia and mild mental confusion. A few days after that he underwent gamma knife treatment for six lesions.	Right-sided weakness affecting predominantly the face and right arm over the last two weeks. Deteriorating motor function.	Woke up with headache in the morning that persisted throughout day. No fever or chills, reports no new neurological symptoms. Did report feeling sleepy.
**Visit 5**			
Date of Visit (months from initial diagnosis)	13	2	3
KPS	70		70
Mini Mental Status Exam Score		14	
History of seizures since last visit	Yes	No	No
Neurologic Deficit	No	Yes. Right-sided weakness, aphasia.	No
Symptoms Notes	He has been unsteady of foot. There has been no worsening confusion. Appetite improved today. There is no nausea, vomiting, or worsening headache. He is globally weak, but has no focal neurological deficit. Patient is fatigued. The patient is neurologically deteriorating.	Improvement in clinical symptoms.	Significant problems with memory. Failed an attempted dexamethasone taper. Trouble with fatigue, but otherwise tolerated the treatment well.
**Visit 6**			
Date of Visit (months from initial diagnosis)		4	3
KPS			70
Mini Mental Status Exam Score		12	
History of seizures since last visit		No	No
Neurologic Deficit		Yes. Right-sided weakness, aphasia.	No

Symptoms Notes		Some increase in sensory abnormality in the ulnar aspect of the right hand.	Began having headaches one week ago at which point he was taking 1 mg of Decadron daily.
**Visit 7**			
Date of Visit (months from initial diagnosis)		5	
KPS			
Mini Mental Status Exam Score		13	
History of seizures since last visit		No	
Neurologic Deficit		Yes. Right-sided weakness, aphasia.	
Symptoms Notes		Continuing to have deteriorating sensory function of the right hand and also now has begun to note some difficulties in the right leg.	
**Visit 8**			
Date of Visit (months from initial diagnosis)		6	
KPS			
Mini Mental Status Exam Score		16	
History of seizures since last visit		Yes	
Neurologic Deficit		Yes. Right-sided weakness, aphasia.	
Symptoms Notes		Increased right hand numbness; sensation loss in her right ankle/foot.	
**Visit 9**			
Date of Visit (months from initial diagnosis)		7	
KPS			
Mini Mental Status Exam Score		16	
History of seizures since last visit		No	

Neurologic Deficit		Yes. Right-sided weakness, aphasia.	
Symptoms Notes		Sensory disturbance on the right side.	
**Visit 10**			
Date of Visit (months from initial diagnosis)		8	
KPS			
Mini Mental Status Exam Score		21	
History of seizures since last visit		No	
Neurologic Deficit		No. Right-sided weakness, aphasia.	
Symptoms Notes		n/a	
**Visit 11**			
Date of Visit (months from initial diagnosis)		12	
KPS		60	
Mini Mental Status Exam Score			
History of seizures since last visit		No	
Neurologic Deficit		Yes. Right-sided weakness, aphasia.	
Symptoms Notes		Further decrease in motor function on the right side. Walking into the hospital today, pt slipped and fell on right shoulder and has acute right shoulder pain.	
**Visit 12**			
Date of Visit (months from initial diagnosis)		12	
KPS		60	
Mini Mental Status Exam Score			
History of seizures since last visit		No	
Neurologic Deficit		No. Right-sided weakness, aphasia.	

Symptoms Notes		n/a	
**Visit 13**			
Date of Visit (months from initial diagnosis)		13	
KPS		50	
Mini Mental Status Exam Score			
History of seizures since last visit		No	
Neurologic Deficit		Yes. Right-sided weakness, aphasia.	
Symptoms Notes		Gradual decline in neurologic function. This has become more marked over the last two to three days; no longer able to comprehend conversations to any degree, and right arm has become functionless.	
**Clinical Trials**			
1	//	//	//

Patient ID	W10-1-1	W11-1-1	W12-1-1
**Patient Information**			
Gender	Female	Male	Female
Age at Initial Diagnosis	45	58	61
Weight	100.4 kg	82.3 kg	64.0 kg
Height	177.8 cm	177.8 cm	160.0 cm
KPS	100 (?)	100 (?)	70 (?)
Mini Mental Status Exam Score			
History of Seizures Since Last Visit	No	Yes	No
Neurologic Deficit	No	No	Yes. Left. Weakness arm and leg.

Presentation Symptoms	Progressive headache, nausea, blurry vision.	Dizziness, imbalance, and nausea.	Worsening headaches, clumsiness of left leg.
Time to Progression	NA	159 days	NA
Overall Survival	NA	1076 days	80 days
Time to Last Follow-up	1623 days	NA	NA
Cause Of Death	//	Tumor progression	Tumor progression
**Tumor Information**			
**Tumor 1**			
Date (months from initial diagnosis)	0	0	0
Diagnosis	Astrocytic tumor	Astrocytic tumor	Astrocytic tumor
Grade	Glioblastoma WHO grade IV	Glioblastoma WHO grade IV	Glioblastoma WHO grade IV
Location	Left Parietal	Right Parietal	Right Frontal. Right Temporal.
**Medications**			
**Medication 1**			
Category	Anti-epileptic	Steroids	Cholesterol Lowering
Drug Name	KEPPRA	Dexamethasone	-1
Start (months from initial diagnosis)	-4	-2	Pravastatin
Total Daily Dosage	1000-2000mg	2-16mg	20mg
End (months from initial diagnosis)	-3	35	0
**Medication 2**			
Category	Multi-vitamin	Cholesterol Lowering	Anti-depressant
Drug Name		Simvastatin	Citalopram
Start (months from initial diagnosis)	-4	-1	-1
Total Daily Dosage	1 cap daily oral	20mg	40-60mg
End (months from initial diagnosis)	-3	24	0
**Medication 3**			
Category	Anti-epileptic	Anti-epileptic	Antihypertensive
Drug Name	Lamotrigine	Levetiracetam	Metoprolol
Start (months from initial diagnosis)	-3	-1	-1
Total Daily Dosage	25-50mg	100-2000mg	50mg

End (months from initial diagnosis)	16	35	0
**Medication 4**			
Category	Steroids	Anti-depressant	Steroids
Drug Name	Dexamethasone	Sertralite	Dexamethasone
Start (months from initial diagnosis)	-1	3	0
Total Daily Dosage	4-16mg	25mg	4mg/1ml injection 4mg 4x/day
End (months from initial diagnosis)	16	17	0
**Medication 5**			
Category			Anti-epileptic
Drug Name			KEPPRA
Start (months from initial diagnosis)			0
Total Daily Dosage			1000mg in NS (0.9% NaCl) IV solution 100mg 2x/day
End (months from initial diagnosis)			0
**Medication 6**			
Category			Anti-epileptic
Drug Name			Valproic Acid
Start (months from initial diagnosis)			0
Total Daily Dosage			1250mg
End (months from initial diagnosis)			0
**Medication 7**			
Category			Cholesterol Lowering
Drug Name			Simvastatin
Start (months from initial diagnosis)			0
Total Daily Dosage			10mg
End (months from initial diagnosis)			0
**Medication 8**			
Category			Steroids
Drug Name			Dexamethasone
Start (months from initial diagnosis)			0
Total Daily Dosage			2mg 2x/day oral
End (months from initial diagnosis)			1
**Surgery**			
**Surgery 1**			

Date of Surgery (months from initial diagnosis)	0	0	0
Gliadel	No	No	No
Awake mapping	Yes	No	No
Stealth scan	Yes	Yes	Yes
**Tissue Diagnosis**			
**Tissue 1**			
Serial Number	W10-1-1, SN183	W11-1-1, SN187	W12-1-1, SN191
Diagnosis	Glioblastoma WHO grade IV	Glioblastoma WHO grade IV	Glioblastoma WHO grade IV
Location	Left Parietal	Right Parietal	Right Temporal
Extent	Complete resection	Complete resection	Complete resection
MRI confirmation	Yes	Yes	Yes
Notes		motor mapping	frontal not resected
**Tissue 2**			Still Surgery 1
Serial Number			SN191-2
Diagnosis			Normal
Location			Right Temporal
Extent			Complete resection
MRI confirmation			/
Notes			/
**Chemotherapy**			
**Medication 1**			
Drug Name	Temozolomide	Temozolomide	/
Start (months from initial diagnosis)	1	1	/
Dosage	140mg	150mg TD	/
End (months from initial diagnosis)	3	2	/
Description	Concurrent	Concurrent	/
**Medication 2**			
Drug Name	Temozolomide	Temozolomide	

Start (months from initial diagnosis)	3	3	
Dosage	300mg	150mg TD	
End (months from initial diagnosis)	18	5	
Description	maintenance 5x/mo.	Maintenance 5x/mo	
**Medication 3**			
Drug Name		Avastin	
Start (months from initial diagnosis)		7	
Dosage		800mg	
End (months from initial diagnosis)		28	
Description		1x/2wks	
**Medication 4**			
Drug Name		Lomustine	
Start (months from initial diagnosis)		17	
Dosage		200mg QD	
End (months from initial diagnosis)		24	
Description		1dose/month	
**Medication 5**			
Drug Name		Avastin	
Start (months from initial diagnosis)		28	
Dosage		800mg	
End (months from initial diagnosis)			
Description		1x/3wks	
**Radiation Therapy**			
**Medication 1**			
Type	IMRT	IMRT	/
Start (months from initial diagnosis)	1	1	/
Dosage	6300 cGy	4600 cGy	/
End (months from initial diagnosis)	2	2	/
Description		Parietal Lobe	/
Fraction	35	23	/
**Medication 2**			

Type		IMRT	
Start (months from initial diagnosis)		2	
Dosage		1400 cGy	
End (months from initial diagnosis)		2	
Description		Boost	
Fraction		7	
**Medication 3**			
Type		Stereotactic	
Start (months from initial diagnosis)		33	
Dosage		2750	
End (months from initial diagnosis)		33	
Description		Cyberknife	
Fraction		5	
**Pathology**			
**Surgery 1**			
Date Of Report (months from initial diagnosis)	0	0	0
**Report**			
Location	Tissue: W10-1-1, SN183	Tissue: W11-1-1, SN187	Tissue: W12-1-1, SN191
Histopathology	Glioblastoma, WHO grade IV	Glioblastoma, WHO grade IV	Glioblastoma, with small cell features, WHO grade IV
1p19q_deletion	positive	Negative	Negative
EGFR	gain	gain	amplification
PTEN	gain	loss	loss
MGMT PCR	methylated	methylated	unmethylated
MGMT IHC	negative View Slides: A	50%	20%
MGMT MS-MLPA:R1	methylated (0.868646503)	methylated (0.345438822)	methylated (0.449555554)
MGMT MS-MLPA:R2	unmethylated (0)	unmethylated (0)	unmethylated (0.148271849)
MGMT MS-MLPA:R3	methylated (0.869387354)	methylated (0.528812725)	unmethylated (0.076863661)
IDH1 mutation	R132H	wildtype	wildtype
EGFR vIII	not detected	not detected	not detected
**Surgery 2**			

Date Of Report (months from initial diagnosis)			Still surgery 1
**Report**			
Location			Tissue: SN191-2
Histopathology			/
1p19q_deletion			/
EGFR			/
PTEN			/
MGMT PCR			/
MGMT IHC			/
MGMT MS-MLPA:R1			unmethylated ()
MGMT MS-MLPA:R2			unmethylated ()
MGMT MS-MLPA:R3			unmethylated ()
IDH1 mutation			/
EGFR vIII			/
**Follow-up Clinic Visit**			
**Visit 1**			
Date of Visit (months from initial diagnosis)	0	0	*Patient has no follow-up clinic visits*
KPS	100	100	
Mini Mental Status Exam Score			
History of seizures since last visit	No	No	
Neurologic Deficit	Yes. Right homonymous hemianopia.	No	
Symptoms Notes	n/a		
**Visit 2**			
Date of Visit (months from initial diagnosis)	0	0	
KPS	90	100	
Mini Mental Status Exam Score			
History of seizures since last visit	Yes	Yes	
Neurologic Deficit	Yes. Righ homonymous hemianopia.	Yes. Left homonymous hemianopia.	

Symptoms Notes	Mild edema. Mild problems with fatigue.	Stable disease.	
**Visit 3**			
Date of Visit (months from initial diagnosis)	1	0	
KPS	90	100	
Mini Mental Status Exam Score			
History of seizures since last visit	No	No	
Neurologic Deficit	Yes. Right homonymous hemianopia.	No. Left homonymous hemianopia.	
Symptoms Notes	n/a	No new problems to report.	
**Visit 4**			
Date of Visit (months from initial diagnosis)	1	0	
KPS	90	90	
Mini Mental Status Exam Score		30	
History of seizures since last visit	No	No	
Neurologic Deficit	Yes. Right homonymous hemianopia.	Yes. Left visual field extinction.	
Symptoms Notes	/	n/a	
**Visit 5**			
Date of Visit (months from initial diagnosis)	2	1	
KPS	90	90	
Mini Mental Status Exam Score			
History of seizures since last visit	No	Yes	
Neurologic Deficit	Yes. Right homonymous hemianopia.	Yes. Left visual field extinction.	
Symptoms Notes	Persistent headaches throughout treatments.	Head pressure, minor nausea.	
**Visit 6**			
Date of Visit (months from initial diagnosis)	2	1	
KPS	90	80	
Mini Mental Status Exam Score			

History of seizures since last visit	No	No	
Neurologic Deficit	Yes. Right homonymous hemianopia.	Yes. Left visual field extinction.	
Symptoms Notes	n/a	Blurry vision, decreased balance and issues with ability to mentate. Reports feeling woozy. Fatigued from treatment. Not sleeping well at night.	
**Visit 7**			
Date of Visit (months from initial diagnosis)	8	2	
KPS	80	90	
Mini Mental Status Exam Score			
History of seizures since last visit	No	No	
Neurologic Deficit	Yes. Right homonymous hemianopia.	Yes. Left visual field extinction.	
Symptoms Notes	Lightheadedness, some nausea. The headaches are sometimes worse in the morning. Depression, muscle aches.	Vague sense of malaise, no specific complaints referable to treatment.	
**Visit 8**			
Date of Visit (months from initial diagnosis)	9	3	
KPS	80		
Mini Mental Status Exam Score		30	
History of seizures since last visit	No	No	
Neurologic Deficit	Yes. Right homonymous hemianopia.	Yes. Left visual field extinction.	
Symptoms Notes	n/a	Cognitive difficulties. Failed an attempted dexamethasone taper and is now back on 1 mg twice a day.	
**Visit 9**			

Date of Visit (months from initial diagnosis)	11	4	
KPS	80	80	
Mini Mental Status Exam Score			
History of seizures since last visit	No	No	
Neurologic Deficit	Yes. Right homonymous hemianopia.	Yes. Left visual field extinction.	
Symptoms Notes	Some numbness/tingling, which she periodically has in the mornings and less in the afternoons. Depression.	Fatigue, malaise, and low-grade constant nausea have steadily worsened. Wants to get off of Temodar to see if quality of life will improve by any degree.	
**Visit 10**			
Date of Visit (months from initial diagnosis)	12	5	
KPS	80	80	
Mini Mental Status Exam Score		29	
History of seizures since last visit	No	No	
Neurologic Deficit	Yes. Right homonymous hemianopia.	Yes. Left visual field deficit.	
Symptoms Notes	Experienced a couple of severe headaches over the past week. These headaches were diffuse and time-limited to a couple of hours.	Had new transient increase in headache, difficulties with TMZ, which has been discontinued.	
**Visit 11**			
Date of Visit (months from initial diagnosis)	12	5	
KPS	80	90	
Mini Mental Status Exam Score			
History of seizures since last visit	No	No	
Neurologic Deficit	Yes. Right homonymous hemianopia.	Yes. Left visual field deficit.	

Symptoms Notes	Gets headaches from Temodar. No depression. Lightheadedness, numbness, tingling.	Quality of life has substantially improved over the last month, especially after discontinuing TMZ.	
**Visit 12**			
Date of Visit (months from initial diagnosis)	13	7	
KPS	80		
Mini Mental Status Exam Score		29	
History of seizures since last visit	No	No	
Neurologic Deficit	Yes. Right homonymous hemianopia.	Yes. Left homonymous hemianopia.	
Symptoms Notes	n/a	Clinically stable, although there is disease progression.	
**Visit 13**			
Date of Visit (months from initial diagnosis)	13	8	
KPS	80	90	
Mini Mental Status Exam Score			
History of seizures since last visit	No	No	
Neurologic Deficit	Yes. Right homonymous hemianopia.	No. Left homonymous hemianopia.	
Symptoms Notes	Nausea is controlled well with p.r.n. antiemetics.	Denies any difficulty with the Avastin with nausea, vomiting, or diarrhea. No new complaints.	
**Visit 14**			
Date of Visit (months from initial diagnosis)	14	9	
KPS	80	90	
Mini Mental Status Exam Score			
History of seizures since last visit	No	No	
Neurologic Deficit	Yes. Right homonymous hemianopia.	Yes. Left homonymous hemianopia.	

Symptoms Notes	n/a	No new neurological complaints.	
**Visit 15**			
Date of Visit (months from initial diagnosis)	16	9	
KPS	70	90	
Mini Mental Status Exam Score			
History of seizures since last visit	No	No	
Neurologic Deficit	Yes. Right homonymous hemianopia.	Yes. Left homonymous hemianopia.	
Symptoms Notes	Intermittent headaches, some worsening of coordination.	n/a	
**Visit 16**			
Date of Visit (months from initial diagnosis)	19	10	
KPS	70	90	
Mini Mental Status Exam Score			
History of seizures since last visit	No	No	
Neurologic Deficit	Yes. Right homonymous hemianopia.	Yes. Left homonymous hemianopia.	
Symptoms Notes	Some bruising, daily headaches.	n/a	
**Visit 17**			
Date of Visit (months from initial diagnosis)	22	11	
KPS	70	80	
Mini Mental Status Exam Score			
History of seizures since last visit	No	No	
Neurologic Deficit	Yes. Right homonymous hemianopia.	No	

Symptoms Notes	Headaches, balance problems.	Continues to battle some mild nausea with the infusions, and occasional headaches, but these are unchanged over months, and readily tolerable.	
**Visit 18**			
Date of Visit (months from initial diagnosis)	25	11	
KPS	70	80	
Mini Mental Status Exam Score			
History of seizures since last visit	No	No	
Neurologic Deficit	Yes. Right homonymous hemianopia.	No. Left homonymous hemianopia.	
Symptoms Notes	Mild dysmetria on the left, daily headaches.	/	
**Visit 19**			
Date of Visit (months from initial diagnosis)	28	12	
KPS	70	80	
Mini Mental Status Exam Score			
History of seizures since last visit	No	Yes	
Neurologic Deficit	Yes. Right homonymous hemianopia.	Yes. Left homonymous hemianopia.	
Symptoms Notes	Headache, neck pain.	Fatigue	
**Visit 20**			
Date of Visit (months from initial diagnosis)	33	17	
KPS	60	70	
Mini Mental Status Exam Score			
History of seizures since last visit	No	No	
Neurologic Deficit	Yes. Right homonymous hemianopia.	Yes. Left homonymous hemianopia.	

Symptoms Notes	Difficulty with balance, mild dysmetria with right hand.	Disease is progressing, starting CCNU.	
**Visit 21**			
Date of Visit (months from initial diagnosis)	41	21	
KPS	80	80	
Mini Mental Status Exam Score			
History of seizures since last visit	No	No	
Neurologic Deficit	No	Yes. Left homonymous hemianopia.	
Symptoms Notes	No residual enhancement and there is no change or any sign of tumor recurrence.	Stabilized from clinical standpoint and the new MRI scan shows no evidence of further progression.	
**Visit 22**			
Date of Visit (months from initial diagnosis)	45	23	
KPS	70	70	
Mini Mental Status Exam Score			
History of seizures since last visit	No	No	
Neurologic Deficit	No	Yes. Left homonymous hemianopia.	
Symptoms Notes	Patient has no significant new symptoms or problems. She has chronic headache related to her glioblastoma.	Mild clinical deterioration.	
**Visit 23**			
Date of Visit (months from initial diagnosis)	53	24	
KPS	70	80	
Mini Mental Status Exam Score			
History of seizures since last visit	No	No	
Neurologic Deficit	No	No	

Symptoms Notes	She reports that she is doing well and without new complaints.	n/a	
**Visit 24**			
Date of Visit (months from initial diagnosis)	59	26	
KPS		80	
Mini Mental Status Exam Score			
History of seizures since last visit	No	No	
Neurologic Deficit	No	Yes. Left homonymous hemianopia.	
Symptoms Notes	Follow-up visit.	No new problems.	
**Visit 25**			
Date of Visit (months from initial diagnosis)		30	
KPS		80	
Mini Mental Status Exam Score			
History of seizures since last visit		No	
Neurologic Deficit		Yes. Left homonymous hemianopia.	
Symptoms Notes		New MRI data is consistent with stable disease.	
**Visit 26**			
Date of Visit (months from initial diagnosis)		31	
KPS		70	
Mini Mental Status Exam Score			
History of seizures since last visit		No	
Neurologic Deficit		Yes. Left homonymous hemianopia.	
Symptoms Notes		Deteriorating left leg function; dragging his leg more and has had some falls.	

**Visit 27**			
Date of Visit (months from initial diagnosis)		32	
KPS		60	
Mini Mental Status Exam Score			
History of seizures since last visit		No	
Neurologic Deficit		Yes. Left homonymous hemianopsia, left hemiparesis.	
Symptoms Notes		Worsening left hemiparesis; disease may be progressing on Avastin.	
**Clinical Trials**			
1	//	//	//

Patient ID	W13-1-1	W16-1-1	W19-1-1
**Patient Information**			
Gender	Female	Male	Female
Age at Initial Diagnosis	60	77	68
Weight	95 kg	83 kg	65.722 kg
Height	160 cm	183,88	168.64 cm
KPS	90 (?)	100 (?)	80 (?)
Mini Mental Status Exam Score			
History of Seizures Since Last Visit	No	No	No
Neurologic Deficit	No	No	No
Presentation Symptoms	Personality changes.	Left hand weakness, cognitive changes.	Speech disturbances.
Time to Progression	235 days	97 days	NA
Overall Survival	250 days	353 days	615 days
Time to Last Follow-up	NA	NA	NA
Cause Of Death	Tumor progression	Tumor progression	Tumor progression
**Tumor Information**			
**Tumor 1**			
Date (months from initial diagnosis)	0	0	0
Diagnosis	Astrocytic tumor	Astrocytic tumor	Astrocytic tumor

Grade	Glioblastoma WHO grade IV	Glioblastoma WHO grade IV	Glioblastoma WHO grade IV
Location	Right Parietal	Right Frontal	Left Temporal
**Medications**			
**Medication 1**			
Category	Anti-epileptic	Steroids	Anti-depressant
Drug Name	Levetiracetam	Dexamethasone	Fluoxetine
Start (months from initial diagnosis)	0	-1	0
Total Daily Dosage	1000mg	1mg daily oral	20mg QD
End (months from initial diagnosis)	0	1	0
**Medication 2**			
Category	Steroids	Anti-epileptic	Steroids
Drug Name	Dexamethasone	Phenytoin	Dexamethasone
Start (months from initial diagnosis)	0	0	0
Total Daily Dosage	4-16mg	300mg	2-16mg
End (months from initial diagnosis)	8	0	2
**Medication 3**			
Category	Anti-depressant	Multi-vitamin	Anti-epileptic
Drug Name	Quetiapine	ONE-A-DAY	Phenytoin
Start (months from initial diagnosis)	0	0	0
Total Daily Dosage	12.5-25mg	1cap daily oral	300mg
End (months from initial diagnosis)	1	0	0
**Medication 4**			
Category	Anti-epileptic	Anti-depressant	Cholesterol Lowering
Drug Name	Divalproex	Mirtazapine	Simvastatin
Start (months from initial diagnosis)	0	0	0
Total Daily Dosage	125-1000mg	15mg	80mg
End (months from initial diagnosis)	1	3	0
**Medication 5**			
Category	Anti-depressant	Anti-epileptic	Antihypertensive
Drug Name	Risperidone	KEPPRA	Diltiazem
Start (months from initial diagnosis)	0	0	0
Total Daily Dosage	0.5-1.5mg	1000mg	120mg
End (months from initial diagnosis)	7	3	0
**Medication 6**			
Category	Anti-epileptic	Cholesterol Lowering	Antihypertensive

Drug Name	Fosphenytoin	Warfarin	Hydrochlorothiazide
Start (months from initial diagnosis)	7	0	0
Total Daily Dosage	300-2000mg	2.8-7.5mg	12.5mg
End (months from initial diagnosis)	8	9	0
**Medication 7**			
Category	Anti-epileptic		Anti-epileptic
Drug Name	Lacosamide		KEPPRA
Start (months from initial diagnosis)	8		0
Total Daily Dosage	100-200mg		1000mg
End (months from initial diagnosis)	8		2
**Medication 8**			
Category			Cholesterol Lowering
Drug Name			Lovastatin
Start (months from initial diagnosis)			1
Total Daily Dosage			80mg
End (months from initial diagnosis)			1
**Surgery**			
**Surgery 1**			
Date of Surgery (months from initial diagnosis)	0	0	0
Gliadel	No	No	No
Awake mapping	No	Yes	Yes
Stealth scan	Yes	Yes	Yes
**Tissue Diagnosis**			
**Tissue 1**			
Serial Number	W13-1-1, SN195	W16-1-1, SN206	W19-1-1, SN214
Diagnosis	Glioblastoma WHO grade IV	Glioblastoma WHO grade IV	Glioblastoma WHO grade IV
Location	Right Parietal	Right Frontal	Left Temporal
Extent	Complete resection	Complete resection	Complete resection
MRI confirmation	Yes	Yes	Yes
**Tissue 2**	Still surgery 1		
Serial Number	SN195-2		
Diagnosis	Normal		

Location	Right Parietal		
Extent	Complete resection		
MRI confirmation	Yes		
**Chemotherapy**			
**Medication 1**			
Drug Name	Temozolomide	Temozolomide	Temozolomide
Start (months from initial diagnosis)	1	0	1
Dosage	140mg	150mg	130mg
End (months from initial diagnosis)	2	3	2
Description	Concurrent	Daily oral during radiation	Concurrent
**Medication 2**			
Drug Name	Temozolomide		
Start (months from initial diagnosis)	2		
Dosage	140mg		
End (months from initial diagnosis)	8		
Description	Maintenance 21x/mo		
**Radiation Therapy**			
**Medication 1**			
Type	IMRT	IMRT	IMRT
Start (months from initial diagnosis)	2	0	1
Dosage	5940 cGy	4500 cGy	4600 cGy
End (months from initial diagnosis)	2	1	2
Description	Concurrent w/temodar	Concurrent	Concurrent
Fraction	33	25	23
**Medication 2**			
Type		IMRT	IMRT
Start (months from initial diagnosis)		1	2
Dosage		1440 cGy	1200 cGy
End (months from initial diagnosis)		2	2
Description		Boost	Concurrent
Fraction		8	6/7 franctions completed
**Pathology**			
**Surgery 1**			

Date Of Report (months from initial diagnosis)	0	0	0
**Report**			
Location	Tissue: W13-1-1, SN195	Tissue: W16-1-1, SN206	Tissue: W19-1-1, SN214
Histopathology	Glioblastoma	Glioblastoma, with small cell features, WHO grade IV	Glioblastoma, WHO grade IV
1p19q_deletion	Negative	Negative	Negative
EGFR	amplification	amplification	gain
PTEN	loss	loss	negative
MGMT PCR	unmethylated	unmethylated	unmethylated
MGMT IHC	50%	25%	30%
MGMT MS-MLPA:R1	unmethylated (0.16)	unmethylated (0.15)	methylated (0.82)
MGMT MS-MLPA:R2	unmethylated (0.03)	unmethylated (0.03)	unmethylated (0.14)
MGMT MS-MLPA:R3	unmethylated (0)	unmethylated (0.03)	unmethylated (0.07)
IDH1 mutation	wildtype	wildtype	wildtype
EGFR vIII	detected	detected	not detected
**Surgery 2**			
Date Of Report (months from initial diagnosis)	Still Surgery 1		
**Report**			
Location	Tissue: SN195-2		
Histopathology			
1p19q_deletion			
EGFR			
PTEN			
MGMT PCR			
MGMT IHC			
MGMT MS-MLPA:R1	methylated (0.34)		
MGMT MS-MLPA:R2	unmethylated (0.06)		
MGMT MS-MLPA:R3	unmethylated (0.02)		
IDH1 mutation			
EGFR vIII			
**Follow-up Clinic Visit**			

**Visit 1**			
Date of Visit (months from initial diagnosis)	1	3	0
KPS	90	90	80
Mini Mental Status Exam Score			
History of seizures since last visit	No	No	No
Neurologic Deficit	Yes. Right mild visual field cut.	No	Yes. Left sided hemiparesis.
Symptoms Notes	n/a	He has done reasonably well with only moderate fatigue associated with his radiation and Temodar treatment.	Occasional problems with word finding. Slight imbalance.
**Visit 2**			
Date of Visit (months from initial diagnosis)	2	3	3
KPS	90	80	70
Mini Mental Status Exam Score			
History of seizures since last visit	No	No	No
Neurologic Deficit	Yes. Left field cut versus left neglect.	No	Yes. Left sided hemiparesis.
Symptoms Notes	Profound left neglect, although otherwise stable.	He has had some mild left-sided weakness but otherwise had not had any other serious symptoms.	n/a
**Visit 3**			
Date of Visit (months from initial diagnosis)	2	5	
KPS	90	80	
Mini Mental Status Exam Score			
History of seizures since last visit	No	No	
Neurologic Deficit	Yes. Left field cut versus left neglect.	No	
Symptoms Notes	Clinically stable.	Clinically doing fairly well, post-RT MRI shows enhancement; increase TMZ dosage.	

**Visit 4**			
Date of Visit (months from initial diagnosis)	4	7	
KPS	90	70	
Mini Mental Status Exam Score			
History of seizures since last visit	No	No	
Neurologic Deficit	Yes. Left field cut versus left neglect.	No	
Symptoms Notes	Clinically stable.	Hospitalized for 3 days about a month ago, because of left hand/arm weakness and numbness.	
**Visit 5**			
Date of Visit (months from initial diagnosis)	4	9	
KPS	80	60	
Mini Mental Status Exam Score			
History of seizures since last visit	No	No	
Neurologic Deficit	Yes. Left field cut versus left neglect.	No	
Symptoms Notes	Notes more fatigue, otherwise stable.	Numbness, tingling sensation, and unsteadiness.	
**Visit 6**			
Date of Visit (months from initial diagnosis)	5		
KPS	80		
Mini Mental Status Exam Score			
History of seizures since last visit	No		
Neurologic Deficit	Yes. Left field cut versus left neglect.		
Symptoms Notes	More fatigue, otherwise unchanged.		
**Visit 7**			
Date of Visit (months from initial diagnosis)	6		
KPS	80		

Mini Mental Status Exam Score			
History of seizures since last visit	No		
Neurologic Deficit	Yes. Left field cut versus left neglect.		
Symptoms Notes	Notes more fatigue. She continues to have profound left neglect.		
**Visit 8**			
Date of Visit (months from initial diagnosis)	7		
KPS	70		
Mini Mental Status Exam Score			
History of seizures since last visit	No		
Neurologic Deficit	Yes. Left field cut versus left neglect.		
Symptoms Notes	Has had more difficulty with incontinence, often requiring more assistance for tranfers.		
**Visit 9**			
Date of Visit (months from initial diagnosis)	7		
KPS	60		
Mini Mental Status Exam Score			
History of seizures since last visit	No		
Neurologic Deficit	No		
Symptoms Notes	Emergency room: patient had clonic activity of the left arm and to a lesser degree of the left leg. Patient status deteriorating.		
**Clinical Trials**			
1	//	//	//

**Patient ID**	**W20-2-1**	**W21-1-1**	**W22-1-1/W22-2-1**

Patient Information			
Gender	Male	Female	Female
Age at Initial Diagnosis	44	55	53
Weight	67.8 kg	62.143 kg	73.03 kg
Height	186.42 cm	166.1 cm	155.94 cm
KPS	90 (?)	80 (?)	90 (?)
Mini Mental Status Exam Score			
History of Seizures Since Last Visit	No	No	No
Neurologic Deficit	No	Yes. Left.	Yes. Left Right. Word finding difficulties, slurred speech.
Presentation Symptoms	Headaches, some mental confusion, and dizziness.	Constant headache, some weakness and clumsiness on the left side.	word finding difficulties, slurred speech and altered mental status
Time to Progression	103 days	93 days	753 days
Overall Survival	363 days	446 days	NA
Time to Last Follow-up	NA	NA	1634 days
Cause Of Death	Tumor progression	Tumor progression	//
**Tumor Information**			
**Tumor 1**			
Date (months from initial diagnosis)	0	0	0
Diagnosis	Astrocytic tumor	Astrocytic tumor	Astrocytic tumor
Grade	Glioblastoma WHO grade IV	Glioblastoma WHO grade IV	Glioblastoma WHO grade IV
Location	Right Temporal	Right Temporal	Left Temporal
**Tumor 2**			
Date (months from initial diagnosis)	6		
Diagnosis	Astrocytic tumor		
Grade	Glioblastoma WHO grade IV		
Location	Right Parietal Right Temporal		
**Medications**			
**Medication 1**			
Category	Anti-epileptic	Steroids	Anti-epileptic

Drug Name	KEPPRA	Dexamethasone	KEPPRA
Start (months from initial diagnosis)	3	-1	-1
Total Daily Dosage	1000-750mg 2x/d oral	2-16mg	500mg BID
End (months from initial diagnosis)	8	0	0
**Medication 2**			
Category	Steroids	Anti-epileptic	Steroids
Drug Name	Dexamethasone	Fosphenytoin (CEREBYX)	Dexamethasone
Start (months from initial diagnosis)	6	-1	0
Total Daily Dosage	2-16mg	1000mg in NS(0.9%NaCl) IV solution 100mg once	4mg QID taper to 3mg QD
End (months from initial diagnosis)	8	//	1
**Medication 3**			
Category		Anti-epileptic	Anti-epileptic
Drug Name		KEPPRA	KEPPRA
Start (months from initial diagnosis)		-1	0
Total Daily Dosage		750-1000-2000mg oral 2x/d	1000mg 2x/d oral
End (months from initial diagnosis)		0	3
**Medication 4**			
Category		Antihypertensive	Anti-epileptic
Drug Name		Hydrochlorothiazide	KEPPRA
Start (months from initial diagnosis)		-1	3
Total Daily Dosage		12.5mg	500mg 2x/d oral
End (months from initial diagnosis)		0	25
**Medication 5**			
Category		Anti-depressant	Anti-depressant
Drug Name		Quetiapine	Amitriptyline
Start (months from initial diagnosis)		-1	12
Total Daily Dosage		50-150mg	25mg QD
End (months from initial diagnosis)		0	13
**Medication 6**			
Category		Antihypertensive	Steroids
Drug Name		Olmesartan	Dexamethasone
Start (months from initial diagnosis)		0	25
Total Daily Dosage		10mg daily oral	4mg 4x/d, 4mh 3x/d
End (months from initial diagnosis)		0	26

**Medication 7**			
Category		Anti-epileptic	Anti-epileptic
Drug Name		Phenytoin	KEPPRA
Start (months from initial diagnosis)		0	25
Total Daily Dosage		400-500mg	1000mg 2x/d oral
End (months from initial diagnosis)		4	38
**Medication 8**			
Category		Steroids	Steroids
Drug Name		Dexamethasone	Dexamethasone
Start (months from initial diagnosis)		3	30
Total Daily Dosage		2mg daily oral	1mg 2x/d oral
End (months from initial diagnosis)		4	33
**Medication 9**			
Category		Anti-epileptic	Anti-epileptic
Drug Name		KEPPRA	KEPPRA
Start (months from initial diagnosis)		4	38
Total Daily Dosage		500mg 2x/d oral	500mg 2x/d oral
End (months from initial diagnosis)		5	55
**Medication 10**			
Category		Cholesterol Lowering	
Drug Name		Rosuvastatin	
Start (months from initial diagnosis)		4	
Total Daily Dosage		10mg	
End (months from initial diagnosis)		5	
**Medication 11**			
Category		Steroids	
Drug Name		Dexamethasone	
Start (months from initial diagnosis)		11	
Total Daily Dosage		4mg 2x/d oral	
End (months from initial diagnosis)		12	
**Surgery**			
**Surgery 1**			
Date of Surgery (months from initial diagnosis)	0	0	0
Gliadel	No	No	No

Awake mapping	No	No	Yes
Stealth scan	Yes	Yes	Yes
**Surgery 2**			
Date of Surgery (months from initial diagnosis)	6		25
Gliadel	No		No
Awake mapping	No		Yes
Stealth scan	Yes		Yes
**Tissue Diagnosis**			
**Tissue 1**			
Serial Number		W21-1-1, SN217	W22-1-1, SN218
Diagnosis		Glioblastoma WHO grade IV	Glioblastoma WHO grade IV
Location		Right Frontal-Parietal-Temporal	Left Temporal
Extent		Complete resection	Complete resection
MRI confirmation		Yes	Yes
**Tissue 2**	Surgery 2		
Serial Number	W20-2-1, SN215		W22-2-1, SN319
Diagnosis	Glioblastoma WHO grade IV		Anaplastic astrocytoma WHO grade III
Location	Right Parietal Right Temporal		Left Temporal
Extent	Complete resection		Complete resection
MRI confirmation	Yes		
**Chemotherapy**			
**Medication 1**			
Drug Name	Temozolomide	Temozolomide	Temozolomide
Start (months from initial diagnosis)	1	0	0
Dosage	75mg/m2	150mg TD	140mg
End (months from initial diagnosis)	2	2	3
Description	Maintenance	Concurrent	daily oral
**Medication 2**			
Drug Name	Temozolomide	Temozolomide	Temozolomide
Start (months from initial diagnosis)	4	3	3

Dosage	140mg/day	120mg TD	340mg
End (months from initial diagnosis)	6	9	13
Description	Concurrent	maintenance 12x/mo	daily oral
**Medication 3**			
Drug Name	Avastin	Avastin	Temozolomide
Start (months from initial diagnosis)	8	3	25
Dosage		525-620mg	125mg
End (months from initial diagnosis)	11	10	daily oral
Description		in NS(0.9% NaCl) IV solution every 2 weeks	34
**Medication 4**			
Drug Name			Temozolomide
Start (months from initial diagnosis)			34
Dosage			125mg
End (months from initial diagnosis)			35
Description			everyday for 21d
**Medication 5**			
Drug Name			Temozolomide
Start (months from initial diagnosis)			36
Dosage			125mg
End (months from initial diagnosis)			36
Description			everyday for 21d
**Medication 6**			
Drug Name			Temozolomide
Start (months from initial diagnosis)			37
Dosage			125mg
End (months from initial diagnosis)			38
Description			everyday for 21d
**Medication 7**			
Drug Name			Etoposide
Start (months from initial diagnosis)			40
Dosage			100mg daily oral

End (months from initial diagnosis)			44
Description			at bedtime
**Medication 8**			
Drug Name			Etoposide
Start (months from initial diagnosis)			44
Dosage			50mg daily oral
End (months from initial diagnosis)			45
Description			at bedtime
**Medication 9**			
Drug Name			Avastin
Start (months from initial diagnosis)			46
Dosage			665-700mg IV
End (months from initial diagnosis)			54
Description			every 2 weeks
**Radiation Therapy**			
**Medication 1**			
Type	IMRT	IMRT	IMRT
Start (months from initial diagnosis)	1	0	0
Dosage	4600 cGy	4500cGy	4600cGy
End (months from initial diagnosis)	2	1	1
Description	Concurrent	To treat the tumor bed in the right temporal fossa	Concurrent
Fraction	23	25	23
**Medication 2**			
Type	IMRT	IMRT	IMRT
Start (months from initial diagnosis)	2	1	1
Dosage	1400 cGy	1260cGy	1400cGy
End (months from initial diagnosis)	2	2	2
Description	Boost	Boost	Boost
Fraction	7	7	7
**Medication 3**			
Type		Stereotactic	?
Start (months from initial diagnosis)		3	27
Dosage		1000cGy	1500cGy
End (months from initial diagnosis)			27

Description		Cyberknife	//
Fraction		1	5
**Medication 4**			
Type		Stereotactic	
Start (months from initial diagnosis)		9	
Dosage		1000cGy	
End (months from initial diagnosis)			
Description		Cyberknife	
Fraction		1	
**Pathology**			
**Surgery 1**			
Date Of Report (months from initial diagnosis)		12	0
**Report**			
Location		Tissue: W21-1-1, SN217	Tissue: W22-1-1, SN218
Histopathology		Glioblastoma	Edematous glioma
1p19q_deletion		Negative	Negative
EGFR		amplification in small population	amplification
PTEN		loss	loss
MGMT PCR		unmethylated	methylated
MGMT IHC		50%	20%
MGMT MS-MLPA:R1		unmethylated (0.23)	unmethylated (0.22)
MGMT MS-MLPA:R2		unmethylated (0.03)	unmethylated (0.00)
MGMT MS-MLPA:R3		unmethylated (0.02)	methylated (0.67)
IDH1 mutation		wildtype	wildtype
EGFR vIII		not detected	detected
**Surgery 2**			
Date Of Report (months from initial diagnosis)	6		25
**Report**			
Location	Tissue: W20-2-1, SN215		Tissue: W22-2-1, SN319
Histopathology	recurrent glioblastoma		Anaplastic astrocytoma, WHO grade III / IV

1p19q_deletion	negative		Negative
EGFR	gain		amplification
PTEN	gain		loss
MGMT PCR	unmethylated		
MGMT IHC	80%		
MGMT MS-MLPA:R1	methylated (0.5)		unmethylated ()
MGMT MS-MLPA:R2	unmethylated (0.05)		unmethylated ()
MGMT MS-MLPA:R3	unmethylated (0.02)		unmethylated ()
IDH1 mutation	wildtype		
EGFR vIII	not detected		
**Follow-up Clinic Visit**			
**Visit 1**			
Date of Visit (months from initial diagnosis)	1	0	0
KPS	100	90	100
Mini Mental Status Exam Score			
History of seizures since last visit	No	No	No
Neurologic Deficit	No	No	No
Symptoms Notes	n/a	Blurred vision after diagnosis.	n/a. Chemo planning.
**Visit 2**			
Date of Visit (months from initial diagnosis)	2	1	3
KPS	100	90	100
Mini Mental Status Exam Score			
History of seizures since last visit	No	No	No
Neurologic Deficit	No	No	No
Symptoms Notes	//	n/a	n/a
**Visit 3**			
Date of Visit (months from initial diagnosis)	3	1	6
KPS		90	100
Mini Mental Status Exam Score	29		
History of seizures since last visit	No	No	No

Neurologic Deficit	No	No	No
Symptoms Notes	//	n/a	n/a
**Visit 4**			
Date of Visit (months from initial diagnosis)	3	1	12
KPS		90	90
Mini Mental Status Exam Score	30		
History of seizures since last visit	No	No	Yes
Neurologic Deficit	No	No	No
Symptoms Notes	Slight imbalance when walking.	n/a	n/a. MRI followup.
**Visit 5**			
Date of Visit (months from initial diagnosis)	4	2	13
KPS	90	90	90
Mini Mental Status Exam Score			
History of seizures since last visit	Yes	No	No
Neurologic Deficit	No	No	No
Symptoms Notes	The Novacure device has caused some slight burns on scalp.	n/a	n/a. Post maint. Chemo. Followup.
**Visit 6**			
Date of Visit (months from initial diagnosis)	5	3	16
KPS		80	90
Mini Mental Status Exam Score	30		
History of seizures since last visit	No	No	No
Neurologic Deficit	No	No	No
Symptoms Notes	n/a	n/a	n/a. Post chemo MRI followup.
**Visit 7**			
Date of Visit (months from initial diagnosis)	5	3	18

KPS	90	80	90
Mini Mental Status Exam Score			
History of seizures since last visit	No	No	No
Neurologic Deficit	No	No	No
Symptoms Notes		n/a	n/a. Post chemo MRI followup.
**Visit 8**			
Date of Visit (months from initial diagnosis)	6	4	21
KPS		80	80
Mini Mental Status Exam Score	30		
History of seizures since last visit	Yes	No	No
Neurologic Deficit	No	No	No
Symptoms Notes	Increased headaches.	n/a	Some occasions where memory does not seem perfect. Post chemo MRI followup.
**Visit 9**			
Date of Visit (months from initial diagnosis)	7	4	24
KPS	80	80	80
Mini Mental Status Exam Score			
History of seizures since last visit	No	No	No
Neurologic Deficit	No	No	No
Symptoms Notes	Pleasant, awake, alert, cooperative, but markedly worse than when seen last. He presented 3 weeks ago with headaches and increasing psudeomeningocele over his craniotomy flap. Reports memory changes postop. He has a significant left vision field cut but otherwise feels well.	n/a	Deterioration in spontaneous language, word finding problems; mri shows signs of progressive disease.<br><br>planned: repeat craniotomy, Avastin, CCNU. MRI followup.

**Visit 10**			
Date of Visit (months from initial diagnosis)	8	4	26
KPS	70	80	70
Mini Mental Status Exam Score			
History of seizures since last visit	No	No	No
Neurologic Deficit	Yes. Left sided weakness.	No	No
Symptoms Notes	Increased confusion, increased left sided weakness.	n/a	Difficulty in occasional word finding problems. vision deterioation. start TMZ 21 day cycle. MRI followup.
**Visit 11**			
Date of Visit (months from initial diagnosis)	9	5	32
KPS	60	80	80
Mini Mental Status Exam Score			
History of seizures since last visit	Yes	No	No
Neurologic Deficit	No	No	No
Symptoms Notes	Entered ER for altered mental status and seizure. Continuation of avastin depends on next MRI.	n/a	Is doing well from both clinical and radiographic standpoints
**Visit 12**			
Date of Visit (months from initial diagnosis)	10	5	34
KPS	70	80	80
Mini Mental Status Exam Score			
History of seizures since last visit	No	No	No
Neurologic Deficit	No	No	No

Symptoms Notes	n/a	n/a	Is now able to report the vision problem she has with reading. She describes a line of blurred vision across both eyes when she tries to focus on words. She denies diplopia. Her last vision exam was about 6 months ago. She feels her vision has no changed since her initial surgery.
**Visit 13**			
Date of Visit (months from initial diagnosis)		6	36
KPS		80	80
Mini Mental Status Exam Score			
History of seizures since last visit		No	No
Neurologic Deficit		No	No
Symptoms Notes		//	Doing well, no new problems, no seizures
**Visit 14**			
Date of Visit (months from initial diagnosis)		6	36
KPS		80	70
Mini Mental Status Exam Score			
History of seizures since last visit		Yes	No
Neurologic Deficit		Yes. Left upper lip numbness and droop.	No
Symptoms Notes		One episode of left upper lip numbness and droop several days prior, resolved.	Replace with a new follow-up visit
**Visit 15**			
Date of Visit (months from initial diagnosis)		8	38
KPS		70	80
Mini Mental Status Exam Score			
History of seizures since last visit		No	No

Neurologic Deficit		Yes. Left facial droop.	No
Symptoms Notes		MRI shows disease progression; hold avastin and TMZ.	She has done very well and other than continued problems with speech which is unchanged, she has had no new or progressive symptoms
**Visit 16**			
Date of Visit (months from initial diagnosis)		9	40
KPS		70	80
Mini Mental Status Exam Score			
History of seizures since last visit		No	No
Neurologic Deficit		Yes. Left facial droop.	No
Symptoms Notes		Restart Avastin and TMZ.	She denies changes in weight or appetite. she denies fever, chills or signs of infection.HEENT:she denies changes in vision or hearing. NEURO: she denies headaches, seizures or confusion. Strength and balance are unchanged. RESP/CV: she denies cough, shortness of breath, chest pain or swelling of the arms/ legs. GI: she denies nausea, vomiting, diarrhea or constipation. HEM: she denies unusual bleed of bruising. INTEG: she denies rash or lesions.
**Visit 17**			
Date of Visit (months from initial diagnosis)		11	41
KPS		50	80

Mini Mental Status Exam Score			
History of seizures since last visit		No	No
Neurologic Deficit		Yes. Left facial droop.	No
Symptoms Notes		Difficulty dressing self, difficulty in vision increased, increased facial droop.	She reports significant fatigue and bone pain with the VP 16 which has improved since she stopped taking the medication. We had also started her on Ritalin which has helped her day time fatigue. She feels good now and has no concerns or complaints. She denies headaches and has had no seizures. She denies any new or progressive symptoms.
**Visit 18**			
Date of Visit (months from initial diagnosis)			43
KPS			80
Mini Mental Status Exam Score			
History of seizures since last visit			No
Neurologic Deficit			No
Symptoms Notes			On examination, alert and oriented with clear and fluent speech.
**Visit 19**			
Date of Visit (months from initial diagnosis)			44
KPS			80
Mini Mental Status Exam Score			
History of seizures since last visit			No
Neurologic Deficit			No

Symptoms Notes			She is doing well and has no new concerns or complaints. She is taking Ritalin once daily with improvement in speech and energy. She has occasional headaches. She reports intermittent bilateral wrist pain with certain activities. She has had no seizures and denies any new neurologic symptoms.
**Visit 20**			
Date of Visit (months from initial diagnosis)			46
KPS			80
Mini Mental Status Exam Score			
History of seizures since last visit			No
Neurologic Deficit			No
Symptoms Notes			Is here for a routine visit. She is currently receiving Avastin monotherapy for a progressing left temporal GBM. She is doing very well and tolerating Aatin without any problems. She denies headaches, seizures, CP, SOB, calf or leg pain and she has had no new neurologic symptoms.
**Visit 21**			
Date of Visit (months from initial diagnosis)			46
KPS			80
Mini Mental Status Exam Score			
History of seizures since last visit			No

Neurologic Deficit			No
Symptoms Notes			She is doing well and has no new concerns or complaints. She is taking Ritalin once daily with improvement in speech and energy. She denies headaches. She has had no seizures and denies any new neurologic symptoms.
**Visit 22**			
Date of Visit (months from initial diagnosis)			48
KPS			80
Mini Mental Status Exam Score			
History of seizures since last visit			No
Neurologic Deficit			No
Symptoms Notes			Doing well, no new problems, no sz. Meds: reviewed, no changes. Exam: Alert, fluent in Espaniol (accd to daughter), no drift, gait ok. No new imaging today.
**Visit 23**			
Date of Visit (months from initial diagnosis)			49
KPS			80
Mini Mental Status Exam Score			
History of seizures since last visit			No
Neurologic Deficit			No
Symptoms Notes			Doing well on single agent Avastin. Continue q 2 wk infusions. RTC 4 weeks and MRI/visit in 8 weeks.

**Visit 24**			
Date of Visit (months from initial diagnosis)			51
KPS			80
Mini Mental Status Exam Score			
History of seizures since last visit			No
Neurologic Deficit			No
Symptoms Notes			Status post resection of left temporal glioblastoma, as before. Faint enhancement anterior to the resection cavity and surrounding FLAIR hyperintensity are stable compared to the 2 months ago exam but slightly decreased in extent compared to the 5 months ago exam, consistent with response to therapy. New doctor.
**Visit 25**			
Date of Visit (months from initial diagnosis)			53
KPS			70
Mini Mental Status Exam Score			
History of seizures since last visit			No
Neurologic Deficit			No
Symptoms Notes			She is with a left temporal glioblastoma, currently on Avastin monotherapy since a half a year ago, who presents for interval follow-up MRI. She continues to have stable difficulty with word-finding. Denies new or progressive symptoms.
**Clinical Trials**			
1	//	//	//

Patient ID	W26-1-1	W27-2-1	W28-1-1
**Patient Information**			
Gender	Female	Male	Male
Age at Initial Diagnosis	58	65	69
Weight	48.6 kg	88.5 kg	71.5 kg
Height	148 cm	186 cm	172 cm
KPS	100 (?)	90 (?)	100 (?)
Mini Mental Status Exam Score	30	30	30
History of Seizures Since Last Visit	Yes	Yes	No
Neurologic Deficit	No	No	Yes. Left homonymous hemianopsia.
Presentation Symptoms	Seizure	Seizures.	Headache.
Time to Progression	1005 days	26 days	NA
Overall Survival	1293 days	72 days	300 days
Time to Last Follow-up	NA	NA	NA
Cause Of Death	Tumor progression	Tumor progression	Tumor progression
**Tumor Information**			
**Tumor 1**			
Date (months from initial diagnosis)	0	0	0
Diagnosis	Astrocytic tumor	Astrocytic tumor	Astrocytic tumor
Grade	Glioblastoma WHO grade IV	Glioblastoma WHO grade IV	Glioblastoma WHO grade IV
Location	Right parietal-temporal	Right Occipital-Temporal	Right Occipital-Parietal
**Medications**			
**Medication 1**			
Category	Anti-epileptic	Anti-epileptic	//
Drug Name	Phenytoin	Keppra	
Start (months from initial diagnosis)	0	-22	
Total Daily Dosage	200mg PO q12hr	500mg BID	
End (months from initial diagnosis)	0	//	
**Medication 2**			
Category	Steroids	Steroids	
Drug Name	Dexamethasone	Dexamethasone	
Start (months from initial diagnosis)	0	-1	
Total Daily Dosage	4mg PO q 6hr	10mg IV	

End (months from initial diagnosis)	0	-1	
**Medication 3**			
Category	Steroids	Steroids	
Drug Name	Dexamethasone	Dexamethasone	
Start (months from initial diagnosis)	0	-1	
Total Daily Dosage	10mg IV	6mg IV BID	
End (months from initial diagnosis)	0	-1	
**Medication 4**			
Category	Steroids	Steroids	
Drug Name	Dexamethasone	Dexamethasone	
Start (months from initial diagnosis)	0	-1	
Total Daily Dosage	4mg IV	2mg IV QD	
End (months from initial diagnosis)	0	-1	
**Medication 5**			
Category	Anti-epileptic	Steroids	
Drug Name	KEPPRA	Dexamethasone	
Start (months from initial diagnosis)	32	-1	
Total Daily Dosage	500mg 2x/d oral	6mg IV	
End (months from initial diagnosis)	34	-1	
**Medication 6**			
Category	Anti-hypertensive	Steroids	
Drug Name	Lisinopril	Dexamethasone	
Start (months from initial diagnosis)	33	-1	
Total Daily Dosage	20mg daily oral	2mg PO BID	
End (months from initial diagnosis)	33	//	
**Medication 7**			
Category	Anti-epileptic		
Drug Name	KEPPRA		
Start (months from initial diagnosis)	34		
Total Daily Dosage	250mg 3x/d oral		
End (months from initial diagnosis)	39		
**Medication 8**			
Category	Steroids		
Drug Name	Dexamethasone		
Start (months from initial diagnosis)	39		

Total Daily Dosage	4mg daily oral		
End (months from initial diagnosis)	40		
**Surgery**			
**Surgery 1**			
Date of Surgery (months from initial diagnosis)	0	0	0
Gliadel	No	No	No
Awake mapping	Yes	No	No
Stealth scan	Yes	Yes	Yes
**Tissue Diagnosis**			
**Tissue 1**			
Serial Number	W26-1-1, SN228	W27-2-1, SN233	W28-1-1, SN234
Diagnosis	Glioblastoma WHO grade IV	Glioblastoma WHO grade IV	Glioblastoma WHO grade IV
Location	Right Parietal-Temporal	Right Occipital-Temporal	Right Occipital-Parietal
Extent	Complete resection	Complete resection	Subtotal resection 90%
MRI confirmation	Yes	Yes	Yes
**Chemotherapy**			
**Medication 1**			
Drug Name	Temozolomide	Temozolomide	//
Start (months from initial diagnosis)	33	-21	
Dosage	65mg	75mg/m2	
End (months from initial diagnosis)	37	-20	
Description	daily oral	Concurrent	
**Medication 2**			
Drug Name	Avastin	Temozolomide	
Start (months from initial diagnosis)	38	-19	
Dosage	411-390mg in NS (0.9%NaCl) IV solution 100mg	100mg/m2	
End (months from initial diagnosis)	39	-19	
Description	once every 2 weeks	maintenance	
**Radiation Therapy**			
**Medication 1**			

Type	//	IMRT	//
Start (months from initial diagnosis)		-21	
Dosage		4600cGy	
End (months from initial diagnosis)		-20	
Description		Concurrent	
Fraction		6	
**Medication 2**			
Type		IMRT	
Start (months from initial diagnosis)		-20	
Dosage		1400cGy	
End (months from initial diagnosis)		-20	
Description		Boost	
Fraction		7	
**Pathology**			
**Surgery 1**			
Date Of Report (months from initial diagnosis)	0	//	//
**Report**			
Location	Tissue: W26-1-1, SN228	Tissue: W27-2-1, SN233	Tissue: W28-1-1, SN234
Histopathology	Glioblastoma	Glioblastoma WHO grade IV	Glioblastoma WHO grade IV
1p19q_deletion			
EGFR			
PTEN			
MGMT PCR			
MGMT IHC			
MGMT MS-MLPA:R1	methylated (0.45)	unmethylated (0.24)	methylated (0.53)
MGMT MS-MLPA:R2	methylated (0.40)	unmethylated (0.06)	unmethylated (0.09)
MGMT MS-MLPA:R3	methylated (0.30)	unmethylated (0.07)	unmethylated (0.08)
IDH1 mutation	wildtype	wildtype	wildtype
EGFR vIII		not detected	not detected
**Follow-up Clinic Visit**			
**Visit 1**			
Date of Visit (months from initial diagnosis)	32	1	0

KPS	60	90	80
Mini Mental Status Exam Score			
History of seizures since last visit	No	Yes	No
Neurologic Deficit	No	Yes. Left neglect.	No
Symptoms Notes	I discussed with her the possible options at this point. I told her that surgical resection is probably not going to give her any clear likelihood of improved survival. If we do surgery I would want functional MRI and I would like to do the surgery with brain mapping. She is going to be seen by Dr. Bankers today and these options will be further discussed and we can make recommendations as to whether or not she should proceed with the surgery.	Moderate to severe headaches despite increasing pain medication.	//
**Visit 2**			
Date of Visit (months from initial diagnosis)	32	1	
KPS	60	70	
Mini Mental Status Exam Score			
History of seizures since last visit	Yes	No	
Neurologic Deficit	No	Yes. Left neglect.	
Symptoms Notes	Denies any new or progressive symptoms.	Hospitalization for severe headaches, draining wound, and oconcern about infection at operative site.	
**Visit 3**			
Date of Visit (months from initial diagnosis)	33	1	
KPS	60	60	
Mini Mental Status Exam Score			

History of seizures since last visit	Yes	No	
Neurologic Deficit	No	Yes. Left neglect.	
Symptoms Notes	Denies any new or progressive symptoms.	Increase in episodic escalations of headaches.	
**Visit 4**			
Date of Visit (months from initial diagnosis)	34		
KPS	60		
Mini Mental Status Exam Score			
History of seizures since last visit	No		
Neurologic Deficit	No		
Symptoms Notes	Denies any new or progressive simptoms.		
**Visit 5**			
Date of Visit (months from initial diagnosis)	35		
KPS	60		
Mini Mental Status Exam Score			
History of seizures since last visit	Yes		
Neurologic Deficit	Yes. Left-sided weakness.		
Symptoms Notes	Denies any new or progressive symptoms.		
**Visit 6**			
Date of Visit (months from initial diagnosis)	37		
KPS	60		
Mini Mental Status Exam Score			
History of seizures since last visit	No		
Neurologic Deficit	No		

Symptoms Notes	She has continued cooking and working around the house, however over the past month, has noted increasing fatigue, and some cognitive decline as well as new falls with some increased difficultly in mobility.		
**Visit 7**			
Date of Visit (months from initial diagnosis)	38		
KPS	50		
Mini Mental Status Exam Score			
History of seizures since last visit	No		
Neurologic Deficit	No		
Symptoms Notes	Over the past month, she has continued to decline markedly at home and is no longer able to tend to daily activities. She is now more reliant on the wheelchair.		
**Visit 8**			
Date of Visit (months from initial diagnosis)	39		
KPS	50		
Mini Mental Status Exam Score			
History of seizures since last visit	No		
Neurologic Deficit	No		

Symptoms Notes	Over the past few weeks, she has become more fatigued with progressive left-sided weakness and neglect. She is now more dependent on the wheelchair. She denies any further seizures. She has decided to move forward with radiation with continuation of Avastin and consideration of Hospice should her symptoms progress.		
**Visit 9**			
Date of Visit (months from initial diagnosis)	40		
KPS			
Mini Mental Status Exam Score			
History of seizures since last visit	No		
Neurologic Deficit	No		
Symptoms Notes	//		
**Clinical Trials**			
1	//	//	//

Patient ID	W29-1-1	W31-1-1	W32-1-1
**Patient Information**			
Gender	Male	Male	Male
Age at Initial Diagnosis	74	18	57
Weight	89.54 kg	179 cm	91.3 kg
Height	179 cm	76.7 kg	183 cm
KPS	100 (?)	90 (?)	90 (?)
Mini Mental Status Exam Score	n/d	27	30
History of Seizures Since Last Visit	No	Yes	Yes
Neurologic Deficit	Yes. Left.	No	No

Presentation Symptoms	Decreased balance, occasional tingling in the tips of digits on left hand, dragging of left foot.	History of multiple seizures and migraine headaches which had been going on for about 2 weeks.	Headaches for 1 month and worsening confusion. Has DVT.
Time to Progression	246 days	312 days	NA
Overall Survival	260 days	871 days	NA
Time to Last Follow-up	NA	NA	1484 days
Cause Of Death	Tumor progression	Tumor progression	//
**Tumor Information**			
**Tumor 1**			
Date (months from initial diagnosis)	0	0	0
Diagnosis	Astrocytic tumor	Astrocytic tumor	Astrocytic tumor
Grade	Glioblastoma WHO grade IV	Glioblastoma WHO grade IV	Glioblastoma WHO grade IV
Location	Right Parietal	Left Frontal	Right Frontal
**Medications**			
**Medication 1**			
Category	Steroids	Anti-epileptic	Anti-epileptic
Drug Name	Dexamethasone	Phenytoin	KEPPRA
Start (months from initial diagnosis)	0	-1	0
Total Daily Dosage	4mg QID decrease to 1mg QD	1000mg in NS(0.9% NaCl) IV solution 50mg once a day	750 mg twice a day oral
End (months from initial diagnosis)	8	0	0
**Medication 2**			
Category	Anti-epileptic	Steroids	Anti-epileptic
Drug Name	KEPPRA	Dexamethasone	Fosphenytoin (CEREBYX)
Start (months from initial diagnosis)	0	0	0
Total Daily Dosage	1000mg in NS(0.9% NaCl) IV solution 100ml	4mg QID taper to 1mg QD	1000 mg in NS(0.9% NaCl) IV solution 100 mg once
End (months from initial diagnosis)	0	0	
**Medication 3**			
Category	Anti-epileptic	Anti-epileptic	Steroids
Drug Name	KEPPRA	KEPPRA	Dexamethasone
Start (months from initial diagnosis)	0	0	0

Total Daily Dosage	500mgx2caps 2x/d oral	1000mg BID decrease to 750mg BID	4mg/1mL injection 4mg 4 times a day, 3mg 4 times a day, 2mg 4 times a day, 2mg 3 times a day, 2mg twice a day
End (months from initial diagnosis)	2	1	0
**Medication 4**			
Category	Anti-epileptic	Anti-epileptic	Anti-epileptic
Drug Name	KEPPRA	Phenytoin	KEPPRA
Start (months from initial diagnosis)	2	1	0
Total Daily Dosage	500mg oral daily	300mg QD	1000 mg in NS(0.9% NaCl) IV solution 100 mg twice a day
End (months from initial diagnosis)	3	3	0
**Medication 5**			
Category	Multi-vitamin	Multi-vitamin	Anti-epileptic
Drug Name		//	KEPPRA
Start (months from initial diagnosis)	4	1	0
Total Daily Dosage	1 cap daily oral	1 cap daily oral	1000 mg twice a day oral
End (months from initial diagnosis)	5	8	0
**Medication 6**			
Category		Anti-epileptic	Steroids
Drug Name		Fosphenytoin (CEREBYX)	Dexamethasone
Start (months from initial diagnosis)		16	0
Total Daily Dosage		100mg PE injection 3x/d	1 mg twice a day, 1mg daily oral
End (months from initial diagnosis)		16	0
**Medication 7**			
Category		Steroids	Anti-epileptic
Drug Name		Dexamethasone	KEPPRA
Start (months from initial diagnosis)		16	0
Total Daily Dosage		4mg/1mL injection 4mg 4 times a day, 3 times a day	1500 mg twice a day oral
End (months from initial diagnosis)		16	11
**Medication 8**			
Category		Anti-epileptic	Anti-epileptic
Drug Name		Phenytoin	KEPPRA XR

Start (months from initial diagnosis)		16	11
Total Daily Dosage		300 mg at bedtime oral	1500 mg every morning and 1000 mg every evening oral
End (months from initial diagnosis)		16	14
**Medication 9**			
Category		Steroids	Anti-epileptic
Drug Name		Dexamethasone	KEPPRA XR
Start (months from initial diagnosis)		16	14
Total Daily Dosage		4 mg 4 times a day, 4mg 3times a day, 3mg 3 times a day, 2mg twice a day oral, 3mg 3 times a day, 2mg 3 times a day, 2mg twice a day	1000 mg twice a day oral
End (months from initial diagnosis)		16	27
**Medication 10**			
Category		Anti-epileptic	Anti-epileptic
Drug Name		KEPPRA	KEPPRA XR
Start (months from initial diagnosis)		21	39
Total Daily Dosage		500 mg twice a day oral	1000 mg twice a day oral
End (months from initial diagnosis)		21	42
**Medication 11**			
Category		Anti-epileptic	
Drug Name		KEPPRA	
Start (months from initial diagnosis)		21	
Total Daily Dosage		1000 mg twice a day oral	
End (months from initial diagnosis)		22	
**Medication 12**			
Category		Anti-epileptic	
Drug Name		KEPPRA XR	
Start (months from initial diagnosis)		23	
Total Daily Dosage		1000 mg twice a day oral	
End (months from initial diagnosis)		27	
**Medication 13**			
Category		Steroids	
Drug Name		Dexamethasone	

Start (months from initial diagnosis)		26	
Total Daily Dosage		4mg/1mL injection 4mg 4 once	
End (months from initial diagnosis)		//	
**Medication 14**			
Category		Steroids	
Drug Name		Dexamethasone	
Start (months from initial diagnosis)		27	
Total Daily Dosage		4mg/1mL injection 10mg	
End (months from initial diagnosis)		//	
**Surgery**			
**Surgery 1**			
Date of Surgery (months from initial diagnosis)	0	0	0
Gliadel	No	No	No
Awake mapping	No	No	No
Stealth scan	Yes	Yes	Yes
**Surgery 2**			
Date of Surgery (months from initial diagnosis)		16	
Gliadel		No	
Awake mapping		Yes	
Stealth scan		Yes	
**Tissue Diagnosis**			
**Tissue 1**			
Serial Number	W29-1-1, SN235	W31-1-1, SN242	W32-1-1, SN243
Diagnosis	Glioblastoma WHO grade IV	Glioblastoma WHO grade IV	Glioblastoma WHO grade IV
Location	Right Parietal	Left Frontal	Right Frontal
Extent	Complete resection	Complete resection	Complete resection
MRI confirmation	Yes	Yes	Yes
Notes		//	//
**Chemotherapy**			
**Medication 1**			
Drug Name	Temozolomide	Temozolomide	Temozolomide

Start (months from initial diagnosis)	0	0	0
Dosage	140mg	140mg	160mg
End (months from initial diagnosis)	2	3	2
Description	concurrent	daily oral	concurrent
**Medication 2**			
Drug Name	Temozolomide	Temozolomide	Temozolomide
Start (months from initial diagnosis)	3	4	2
Dosage	400mg	400mg	400mg
End (months from initial diagnosis)	4	11	11
Description	maintenance 5x/mo	every 4 weeks for 5d oral	maintenance 5x/mo.
**Medication 3**			
Drug Name	Temozolomide	Temozolomide	Temozolomide
Start (months from initial diagnosis)	5	11	11
Dosage	150mg	150mg	160mg
End (months from initial diagnosis)	8	16	13
Description	maintenance 21x/mo	daily oral	maintenance 21x/mo.
**Medication 4**			
Drug Name		Lomustine	Temozolomide
Start (months from initial diagnosis)		16	13
Dosage		220mg	400mg
End (months from initial diagnosis)		21	15
Description		maintenance 1x/6wks	maintenance 5x/mo.
**Medication 5**			
Drug Name		Avastin	
Start (months from initial diagnosis)		17	
Dosage		820 mg in NS(0.9% NaCl) IV solution 100 mg twice a day	
End (months from initial diagnosis)		27	
Description		maintenance 1x/2wks	
**Radiation Therapy**			
**Medication 1**			
Type	IMRT	IMRT	IMRT

Start (months from initial diagnosis)	0	0	0
Dosage	4500cGy	4500cGy	4140cGy
End (months from initial diagnosis)	2	1	1
Description	Concurrent	Concurrent	Concurrent
Fraction	25	25	23
**Medication 2**			
Type	IMRT	IMRT	IMRT
Start (months from initial diagnosis)	2	1	1
Dosage	1800cGy	1440cGy	1800cGy
End (months from initial diagnosis)	2	2	2
Description	Boost	Boost	Boost
Fraction	10	8	10
**Medication 3**			
Type	Stereotactic	Stereotactic	
Start (months from initial diagnosis)	5	10	
Dosage	20Gy	1800cGy	
End (months from initial diagnosis)	5		
Description	Gamma Knife	Cyberknife	
Fraction	1	1	
**Pathology**			
**Surgery 1**			
Date Of Report (months from initial diagnosis)	0	0	0
**Report**			
Location	Tissue: W29-1-1, SN235	Tissue: W31-1-1, SN242	Tissue: W32-1-1, SN243
Histopathology	Glioblastoma	Glioblastoma, WHO grade IV	Glioblastoma, WHO grade IV
1p19q_deletion	Negative	Positive	Negative
EGFR	amplification	gain	gain
PTEN	deletion	deletion	gain
MGMT PCR	unmethylated	unmethylated	methylated
MGMT IHC	10%-15%	75%	negative
MGMT MS-MLPA:R1	methylated (0.41)	unmethylated (0.22)	methylated (0.85)
MGMT MS-MLPA:R2	unmethylated (0.06)	unmethylated (0.05)	unmethylated (0.00)

MGMT MS-MLPA:R3	unmethylated (0.05)	unmethylated (0.03)	methylated (1.02)
IDH1 mutation	wildtype	R132H	wildtype
EGFR vIII	detected	not detected	not detected
**Follow-up Clinic Visit**			
**Visit 1**			
Date of Visit (months from initial diagnosis)	0	0	0
KPS	90		
Mini Mental Status Exam Score		27	30
History of seizures since last visit	No	No	No
Neurologic Deficit	Yes. Left leg weakness.	No	No
Symptoms Notes	Left leg weakness, balance problems. Suture removal.	Mild difficulty with short-term memory. Post-operative MRI.	Taking hot shower when two generalized seizures occured, taken to ER. ER visit.
**Visit 2**			
Date of Visit (months from initial diagnosis)	2	0	0
KPS	80	90	100
Mini Mental Status Exam Score			
History of seizures since last visit	No	No	No
Neurologic Deficit	Yes. Left leg weakness.	No	No
Symptoms Notes	Placement of a subdural to peritoneal shunt due to hydrocephalus. Surgery.	n/a. Suture removal.	Extreme right leg pain. ER visit.
**Visit 3**			
Date of Visit (months from initial diagnosis)	3	1	0
KPS		90	100
Mini Mental Status Exam Score	28		
History of seizures since last visit	No	No	No
Neurologic Deficit	Yes. Left leg weakness.	No	No

Symptoms Notes	Maintenance TMZ planning	n/a. Mid radiation followup.	n/a. Suture removal.
**Visit 4**			
Date of Visit (months from initial diagnosis)	4	2	1
KPS	70	90	90
Mini Mental Status Exam Score			
History of seizures since last visit	Yes	No	No
Neurologic Deficit	No	No	No
Symptoms Notes	Rapid deterioration in gait and memory. Hold TMZ temporarily. MRI followup.	n/a. Post radiation followup.	Patient feels twisting/ripping in his abdomen. ER visit.
**Visit 5**			
Date of Visit (months from initial diagnosis)	5	3	2
KPS	70	90	90
Mini Mental Status Exam Score			
History of seizures since last visit	No	No	No
Neurologic Deficit	Yes. Left-sided weakness.	No	No
Symptoms Notes	Substantial improvement in cognitive function and gait, worsening of left arm/shoulder pain. MRI followup.	Patient unintentionally started TMZ today instead of 4 wks ago, due to miscommunication/confusion. Maintenance TMZ followup.	Fairly significant fatigue. Maintenance TMZ planning.
**Visit 6**			
Date of Visit (months from initial diagnosis)		4	5
KPS		90	90
Mini Mental Status Exam Score			
History of seizures since last visit		No	No
Neurologic Deficit		No	No

Symptoms Notes		n/a. MRI scan followup.	ER visit for syncopal episodes, most consistent with bradyarrhythmia. ER visit.
**Visit 7**			
Date of Visit (months from initial diagnosis)		5	5
KPS		90	90
Mini Mental Status Exam Score			
History of seizures since last visit		Yes	No
Neurologic Deficit		No	No
Symptoms Notes		Took 300mg of TMZ instead of 400 mg TMZ. Neuro oncology followup.	n/a. MRI followup.
**Visit 8**			
Date of Visit (months from initial diagnosis)		6	7
KPS		90	100
Mini Mental Status Exam Score			
History of seizures since last visit		No	No
Neurologic Deficit		No	No
Symptoms Notes		n/a. Neuro oncology followup.	Mild fatigue, constipation, brief episode of near-syncope. MRI followup.
**Visit 9**			
Date of Visit (months from initial diagnosis)		7	9
KPS		90	90
Mini Mental Status Exam Score			
History of seizures since last visit		No	No
Neurologic Deficit		No	No

Symptoms Notes		n/a. Neuro oncology followup.	n/a. MRI followup.
**Visit 10**			
Date of Visit (months from initial diagnosis)		8	11
KPS		90	90
Mini Mental Status Exam Score			
History of seizures since last visit		No	No
Neurologic Deficit		No	No
Symptoms Notes		n/a. MRI followup.	Gradual increase in T2 signal is of unclear cause, changing treatment regimen from 5 to 21 day cycle. MRI followup.
**Visit 11**			
Date of Visit (months from initial diagnosis)		10	13
KPS		80	90
Mini Mental Status Exam Score			
History of seizures since last visit		No	No
Neurologic Deficit		No	No
Symptoms Notes		New MRI scan concerning for progressive disease, switching TMZ to 21 day regimen. MRI scan followup.	More difficulty in terms of prolonged fatigue, switching back to 5 day regimen. MRI followup.
**Visit 12**			
Date of Visit (months from initial diagnosis)		11	15
KPS		80	
Mini Mental Status Exam Score			30
History of seizures since last visit		No	No
Neurologic Deficit		No	No

Symptoms Notes		n/a. MRI followup.	n/a. MRI followup.
**Visit 13**			
Date of Visit (months from initial diagnosis)		12	17
KPS		80	90
Mini Mental Status Exam Score			
History of seizures since last visit		No	No
Neurologic Deficit		No	No
Symptoms Notes		n/a. MRI followup.	n/a. Post-radiation followup.
**Visit 14**			
Date of Visit (months from initial diagnosis)		14	19
KPS		80	80
Mini Mental Status Exam Score			
History of seizures since last visit		No	Yes
Neurologic Deficit		No	No
Symptoms Notes		Increasing enhancement worrisome for tumor progression. MRI followup.	Fatigue, some mild cognitive slowing. Drinks substantial amounts of alcohol daily. MRI followup.
**Visit 15**			
Date of Visit (months from initial diagnosis)		16	21
KPS		70	90
Mini Mental Status Exam Score			
History of seizures since last visit		No	No
Neurologic Deficit		No	No
Symptoms Notes		Progressive disease evident. MRI followup.	Continues to do reasonably well from clinical standpoint and the MRI scan is stable.

**Visit 16**			
Date of Visit (months from initial diagnosis)		16	23
KPS		80	80
Mini Mental Status Exam Score			
History of seizures since last visit		Yes	No
Neurologic Deficit		No	No
Symptoms Notes		n/a. Begin CCNU the following week, then Avastin the week after. post surg. mri followup.	New MRI scan of the brain shows no change compared to prior studies. This is consistent with stable disease.
**Visit 17**			
Date of Visit (months from initial diagnosis)		18	26
KPS		80	90
Mini Mental Status Exam Score			
History of seizures since last visit		No	No
Neurologic Deficit		No	No
Symptoms Notes		n/a. MRI followup.	New MRI scan of the brain shows no changes compared to prior studies. There is no evidence for recurrent disease.
**Visit 18**			
Date of Visit (months from initial diagnosis)		20	28
KPS		70	90
Mini Mental Status Exam Score			
History of seizures since last visit		Yes	No
Neurologic Deficit		No	No

Symptoms Notes		Hypotension; found diaphoretic and unresponsive. ER visit.	No new issues. No seizures. He is getting minimal exercise.
**Visit 19**			
Date of Visit (months from initial diagnosis)		24	30
KPS		80	80
Mini Mental Status Exam Score			
History of seizures since last visit		No	No
Neurologic Deficit		No	No
Symptoms Notes		New MRI scan of the brain shows marked improvement with complete resolution of abnormal contrast enhancement in bilateral frontal and colossal regions.	Doing well except for daytime fatigue and sleepiness. Continues to do well from clinical and radiographic standpoints.
**Visit 20**			
Date of Visit (months from initial diagnosis)		27	32
KPS		60	80
Mini Mental Status Exam Score			
History of seizures since last visit		No	No
Neurologic Deficit		No	No
Symptoms Notes		GBM with subsequent development of hydrocephalus s/p VP shunt placement. He has seen a progressive decline over the past two weeks which is concerning for tumor progression and/or shunt malfunction.	He is doing well and has no new concerns or complaints. He continues to struggle with daytime fatigue despite Nuvigil. He denies any new or progressive symptoms.
**Visit 21**			

Date of Visit (months from initial diagnosis)			34
KPS			80
Mini Mental Status Exam Score			
History of seizures since last visit			No
Neurologic Deficit			No
Symptoms Notes			No new problems. Still with fatigue. Doing well clinically and radiographically.
**Visit 22**			
Date of Visit (months from initial diagnosis)			36
KPS			80
Mini Mental Status Exam Score			
History of seizures since last visit			No
Neurologic Deficit			No
Symptoms Notes			Fatigue but no other new issues. Sleeping several hours during the day. No seizures or headaches. No evidence for progressive tumor.
**Visit 23**			
Date of Visit (months from initial diagnosis)			38
KPS			80
Mini Mental Status Exam Score			
History of seizures since last visit			No
Neurologic Deficit			No
Symptoms Notes			Follow up visit in 2 months with a new MRI scan prior.
**Visit 24**			
Date of Visit (months from initial diagnosis)			40
KPS			80
Mini Mental Status Exam Score			

History of seizures since last visit			No
Neurologic Deficit			No
Symptoms Notes			Excellent stable course from radiographic and clinical standpoints.
**Visit 25**			
Date of Visit (months from initial diagnosis)			42
KPS			80
Mini Mental Status Exam Score			
History of seizures since last visit			No
Neurologic Deficit			No
Symptoms Notes			No new problems. Previously noted fatigue. No seizures.
**Visit 26**			
Date of Visit (months from initial diagnosis)			44
KPS			80
Mini Mental Status Exam Score			
History of seizures since last visit			No
Neurologic Deficit			No
Symptoms Notes			Follow up visit in 2 months with a new MRI scan prior.
**Visit 27**			
Date of Visit (months from initial diagnosis)			46
KPS			80
Mini Mental Status Exam Score			
History of seizures since last visit			No
Neurologic Deficit			No

Symptoms Notes			Presents for interval follow-up with surveillance MRI on observation, as a new patient to me.
**Visit 28**			
Date of Visit (months from initial diagnosis)			48
KPS			90
Mini Mental Status Exam Score			
History of seizures since last visit			No
Neurologic Deficit			No
Symptoms Notes			Presents for interval follow-up with surveillance MRI on observation, now 4 years out from diagnosis, on observation since almost 3 years. Denies new or progressive symptoms. He remotely had one event consistent with possible seizure for which he as remained on Keppra for prophylaxis.
**Clinical Trials**			
1	//	//	NovoCure

Patient ID	W33-1-1	W34-1-1	W36-1-1
**Patient Information**			
Gender	Male	Male	Male
Age at Initial Diagnosis	61	74	62
Weight	83.5 kg	95.7 kg	88.5 kg
Height	183 cm	185 cm	178 cm
KPS	80 (?)	90 (?)	80 (?)
Mini Mental Status Exam Score	25		26
History of Seizures Since Last Visit	No	No	No

Neurologic Deficit	No	Yes. Left/Right. Expressive speech problem.	Yes. Right.
Presentation Symptoms	Fell from bicycle recently. After the fall he had mild headache, confusion, and difficulty concentrating.	One week history of expressive speech problems following aortic root angiogram.	Blurry vision in in his right eye
Time to Progression	NA	170 days	241 days
Overall Survival	NA	351 days	544 days
Time to Last Follow-up	1315 days	NA	NA
Cause Of Death	//	Tumor progression	Tumor progression
**Tumor Information**			
**Tumor 1**			
Date (months from initial diagnosis)	0	0	0
Diagnosis	Astrocytic tumor	Astrocytic tumor	Astrocytic tumor
Grade	Glioblastoma WHO grade IV	Glioblastoma WHO grade IV	Glioblastoma WHO grade IV
Location	Right Parietal	Left Temporal	Left Parietal
**Medications**			
**Medication 1**			
Category	Antihypertensive	Antihypertensive	Cholesterol Lowering
Drug Name	Hydrochlorothiazide	Losartan (COZAAR)	Atorvastatin
Start (months from initial diagnosis)	-1	-1	0
Total Daily Dosage	25mg daily oral	50 mg daily oral	10mg QD
End (months from initial diagnosis)	4	0	//
**Medication 2**			
Category	Steroids	Multi-vitamin	Steroids
Drug Name	Dexamethasone		Dexamethasone
Start (months from initial diagnosis)	0	-1	0
Total Daily Dosage	4mg QID taper to 1mg QD	1 cap daily	4mg QID taper to 1mg QD
End (months from initial diagnosis)	2	2	1
**Medication 3**			
Category	Anti-epileptic	Steroids	Antihypertensive
Drug Name	KEPPRA	Dexamethasone	Metoprolol XL (TOPROL XL)
Start (months from initial diagnosis)	0	-1	0

Total Daily Dosage	1000mg BID decrease to 500mg BID	4mg/mL intravenuos 4 times a day	50mg QD
End (months from initial diagnosis)	3	0	1
**Medication 4**			
Category	Cholesterol Lowering	Anti-epileptic	Anti-depressant
Drug Name	Simvastatin	KEPPRA	Sertraline
Start (months from initial diagnosis)	0	-1	0
Total Daily Dosage	40mg QD	500 mg x 2caps twice a day oral	50mg QD
End (months from initial diagnosis)	0	8	1
**Medication 5**			
Category	Multi-vitamin	Steroids	Anti-epileptic
Drug Name		Dexamethasone	Rosuvastatin (CRESTOR)
Start (months from initial diagnosis)	3	0	0
Total Daily Dosage	1 cap daily oral	4mg 3 times a day, 3mg 3 times a day, 3mg twice a day, 2mg twice a day, 1mg twice a day, 1 mg daily oral	10 mg daily oral
End (months from initial diagnosis)	4	0	0
**Medication 6**			
Category	Cholesterol Lowering	Antihypertensive	Anti-epileptic
Drug Name	Simvastatin	Metoprolol (LOPRESSOR)	KEPPRA
Start (months from initial diagnosis)	4	0	0
Total Daily Dosage	40 mg daily oral	25 mg twice a day for 7 days then 50 mg twice a day oral	1000mg twice a day oral
End (months from initial diagnosis)	4	2	15
**Medication 7**			
Category	Antihypertensive	Steroids	Multi-vitamin
Drug Name	Hydrochlorothiazide	Dexamethasone	ONE-A-DAY
Start (months from initial diagnosis)	9	2	1
Total Daily Dosage	25mg daily oral	2 mg oral twice a day	1 cap daily oral
End (months from initial diagnosis)	10	3	1
**Medication 8**			
Category	Anti-epileptic	Steroids	Antihypertensive
Drug Name	Gabapentin	Prednisone	Verapamil (ISOPTIN)
Start (months from initial diagnosis)	11	4	1

Total Daily Dosage	300 mg 3 times a day, 300 mg twice a day oral	10 mg twice a day oral	2.5 mg injection
End (months from initial diagnosis)	17	6	//
**Medication 9**			
Category	Anti-epileptic	Steroids	Cardiotonic
Drug Name	Gabapentin	Dexamethasone	Clopidogrel
Start (months from initial diagnosis)	17	6	1
Total Daily Dosage	300 mg in the morning and 400 mg in the evening oral	4 mg twice a day till 0.5 daily oral	75mg QD
End (months from initial diagnosis)	19	7	//
**Medication 10**			
Category	Anti-epileptic	Antihypertensive	
Drug Name	Gabapentin	Amlodipine (NORVASC)	
Start (months from initial diagnosis)	19	8	
Total Daily Dosage	100 mg in the morning and 400 mg in the evening oral	2.5 mg daily oral	
End (months from initial diagnosis)	27	9	
**Medication 11**			
Category	Anti-depressant		
Drug Name	Citalopram (CELEXA)		
Start (months from initial diagnosis)	21		
Total Daily Dosage	20 mg at bedtime oral		
End (months from initial diagnosis)	44		
**Medication 12**			
Category	Anti-epileptic		
Drug Name	KEPPRA		
Start (months from initial diagnosis)	27		
Total Daily Dosage	500 mg twice a day oral		
End (months from initial diagnosis)	28		
**Medication 13**			
Category	Anti-epileptic		
Drug Name	KEPPRA		
Start (months from initial diagnosis)	29		
Total Daily Dosage	1000 mg twice a day oral		
End (months from initial diagnosis)	30		
**Medication 14**			

Category	Anti-epileptic		
Drug Name	KEPPRA		
Start (months from initial diagnosis)	32		
Total Daily Dosage	1000 mg twice daily oral		
End (months from initial diagnosis)	34		
**Medication 15**			
Category	Anti-epileptic		
Drug Name	KEPPRA		
Start (months from initial diagnosis)	34		
Total Daily Dosage	1000 mg AM and 1500 mg PM oral		
End (months from initial diagnosis)	40		
**Medication 16**			
Category	Anti-epileptic		
Drug Name	KEPPRA		
Start (months from initial diagnosis)	40		
Total Daily Dosage	1500 mg twice a day oral		
End (months from initial diagnosis)	44		
**Surgery**			
**Surgery 1**			
Date of Surgery (months from initial diagnosis)	0	0	0
Gliadel	No	No	No
Awake mapping	No	Yes	Yes
Stealth scan	Yes	Yes	Yes
**Tissue Diagnosis**			
**Tissue 1**			
Serial Number	W33-1-1, SN245	W34-1-1, SN248	W36-1-1, SN250
Diagnosis	Glioblastoma WHO grade IV	Glioblastoma WHO grade IV	Glioblastoma WHO grade IV
Location	Right Parietal	Left Temporal	Left Parietal
Extent	Complete resection	Complete resection	Subtotal resection 90%
MRI confirmation	Yes	Yes	Yes
**Chemotherapy**			
**Medication 1**			

Drug Name	Temozolomide	Temozolomide	Temozolomide
Start (months from initial diagnosis)	0	0	0
Dosage	140mg QD	150mg	140mg QD
End (months from initial diagnosis)	2	2	2
Description	Concurrent	Concurrent	Concurrent
**Medication 2**			
Drug Name	Temozolomide	Temozolomide	Temozolomide
Start (months from initial diagnosis)	2	3	3
Dosage	300mg QD increase to 360mg QD	300mg	140-280mg
End (months from initial diagnosis)	15	3	15
Description	maintenance once a month 5d cycles	maintenance 5x/mo.	maintenance x4/28 days
**Medication 3**			
Drug Name		Temozolomide	Avastin
Start (months from initial diagnosis)		3	8
Dosage		360mg	10mg/kg (735mg)
End (months from initial diagnosis)		6	12
Description		maintenance 5x/mo.	x1 / 14 days
**Medication 4**			
Drug Name		Temozolomide	Avastin
Start (months from initial diagnosis)		6	13
Dosage		140mg	10 mg/kg
End (months from initial diagnosis)		7	17
Description		concurrent w/ cyberknife, QD	x1 / 21 days
**Medication 5**			
Drug Name		Avastin	Lomustine
Start (months from initial diagnosis)		6	13
Dosage		1000mg	210mg
End (months from initial diagnosis)			14
Description		maintenance 1x/14 days	x1 / 42 days
**Medication 6**			

Drug Name		Temozolomide	Etoposide
Start (months from initial diagnosis)		7	14
Dosage		280mg	1600mg
End (months from initial diagnosis)			15
Description		maintenance 5x/28 days	21x/ 28 days
**Radiation Therapy**			
**Medication 1**			
Type	IMRT	IMRT	IMRT
Start (months from initial diagnosis)	0	0	0
Dosage	4600cGy	6000cGy	46Gy
End (months from initial diagnosis)	1	2	1
Description		Concurrent	
Fraction	23		23
**Medication 2**			
Type	IMRT	Stereotactic	IMRT
Start (months from initial diagnosis)	1	6	1
Dosage	1400cGy	2400cGy	14Gy
End (months from initial diagnosis)	2	6	2
Description	Boost	Cyberknife	Boost
Fraction	7	3	7
**Pathology**			
**Surgery 1**			
Date Of Report (months from initial diagnosis)	0	0	0
**Report**			
Location	Tissue: W33-1-1, SN245	Tissue: W34-1-1, SN248	Tissue: W36-1-1, SN250
Histopathology	Glioblastoma, WHO grade IV	Glioblastoma, WHO grade IV	Glioblastoma, WHO grade IV
1p19q_deletion	Negative	Negative	Negative
EGFR	amplification	gain	gain
PTEN	loss	deletion	gain
MGMT PCR	methylated	unmethylated	unmethylated
MGMT IHC	25%	50%	70%
MGMT MS-MLPA:R1	methylated (0.51)	unmethylated (0.29)	methylated (0.33)

MGMT MS-MLPA:R2	unmethylated (0.13)	unmethylated (0.05)	unmethylated (0.04)
MGMT MS-MLPA:R3	methylated (0.32)	unmethylated (0.03)	unmethylated (0.03)
IDH1 mutation	wildtype	wildtype	wildtype
EGFR vIII	not detected	not detected	not detected
**Follow-up Clinic Visit**			
**Visit 1**			
Date of Visit (months from initial diagnosis)	0	0	0
KPS		90	90
Mini Mental Status Exam Score	25		
History of seizures since last visit	No	No	No
Neurologic Deficit	Yes. Left. Difficulty with left-sided vision.	No. Expressive aphasia.	No
Symptoms Notes	Post surgery followup.	Expressive aphasia improvng. Post surgery followup.	Chronic back pain, difficulty in cognitive retention and reading words. Radiation planning.
**Visit 2**			
Date of Visit (months from initial diagnosis)	2	0	2
KPS	90	90	90
Mini Mental Status Exam Score			
History of seizures since last visit	Yes	No	No
Neurologic Deficit	Yes. Left. Difficulty with left-sided vision.	No. Expressive aphasia.	No
Symptoms Notes	Other than fatigue, anorexia, and constipation, patient tolerated the treatments well. Post radiation followup.	n/a. Chemoradiation planning.	Poor cognitive function. Chemotherapy planning.
**Visit 3**			
Date of Visit (months from initial diagnosis)	4	1	3
KPS	80	90	80
Mini Mental Status Exam Score			
History of seizures since last visit	No	No	No
Neurologic Deficit	Yes. Left. Difficulty with left-sided vision.	No. Expressive aphasia.	Yes. Left.

Symptoms Notes	Gradually progressive neurologic symptoms. Continues to be plagued by fatigue, anorexia, occasional nausea with vomiting, some urinary urgency, and unsteadiness on his feet. Has hydrocephalus. MRI scan.	Speech has being gradually improving. Chemoradiation followup.	Difficulties with word finding skills, left eye fatigue.
**Visit 4**			
Date of Visit (months from initial diagnosis)	4	2	5
KPS	80	80	90
Mini Mental Status Exam Score			
History of seizures since last visit	No	No	No
Neurologic Deficit	Yes. Left. Difficulty with left-sided vision.	No. Expressive aphasia.	No. Expressive dysphasia.
Symptoms Notes	Complains of fatigue, gait disturbance, urinary incontinence, and occasional nausea. Proceeded with left ventriculoperitoneal shunt placement for hydrocephalus.	ER hospitalization for about a week due to imbalance, hypotension, development of pneumonia, and overall failure to thrive; intitiated dexamethasone taper. ER visit.	
**Visit 5**			
Date of Visit (months from initial diagnosis)	6	2	5
KPS	90	90	90
Mini Mental Status Exam Score			
History of seizures since last visit	No	No	No
Neurologic Deficit	Yes. Left. Difficulty with left-sided vision.	No. Expressive aphasia.	No. Expressive dysphasia.

Symptoms Notes	Marked decrease in size of the ventricular system after placement of the posterior left-sided shunt. MRI followup.	Speech gradually improving. Radiation followup.	Lower back pain.
**Visit 6**			
Date of Visit (months from initial diagnosis)	7	2	7
KPS	80	80	
Mini Mental Status Exam Score			26
History of seizures since last visit	No	No	No
Neurologic Deficit	Yes. Left eye difficulty.	No. Expressive aphasia.	No
Symptoms Notes	Slight itching, nausea, and chills.	Admitted to hospital with progressive weakness and dizziness, intermittent loss of hearing in left ear. Hospital visit.	Constipation after TMZ cycle causes near debilitating low back pain. MRI review.
**Visit 7**			
Date of Visit (months from initial diagnosis)	7	3	7
KPS	90	80	80
Mini Mental Status Exam Score			
History of seizures since last visit	No	No	No
Neurologic Deficit	Yes. Left eye difficulty.	No. Expressive aphasia.	No
Symptoms Notes	Once per day, patient has 1-2 min episode of moving visual phenomenon in both visual fields. MRI followup.	n/a. Radiation followup.	Progressive disease. MRI scan.
**Visit 8**			
Date of Visit (months from initial diagnosis)	9	3	8
KPS	90	90	80
Mini Mental Status Exam Score			

History of seizures since last visit	No	No	Yes
Neurologic Deficit	Yes. Left eye difficulty.	No. Expressive aphasia.	No
Symptoms Notes	Dark-colored rash over his right shoulder and right back, 1 brief hive. MRI followup.	Patient doing well, increasing TMZ dosage to 360 mg.	Surgeon decided not to subject the patient to repeat craniotomy, started on Avastin instead.
**Visit 9**			
Date of Visit (months from initial diagnosis)	11	4	9
KPS	80	90	80
Mini Mental Status Exam Score			
History of seizures since last visit	No	No	No
Neurologic Deficit	Yes. Left eye difficulty.	No. Expressive aphasia.	No
Symptoms Notes	Restless leg syndrome (left leg) more noticeable recently. MRI followup.	n/a. Maintenance TMZ followup.	Scan shows marked interval reduction in abnormal enhancement around his tumor.
**Visit 10**			
Date of Visit (months from initial diagnosis)	13	6	11
KPS	90	80	80
Mini Mental Status Exam Score			
History of seizures since last visit	No	No	No
Neurologic Deficit	Yes. Left eye difficulty.	No. Expressive aphasia.	No
Symptoms Notes	n/a. MRI followup.	Worsening of tumor, worsening weakness.	No change in the enhancing nature of the lesion.
**Visit 11**			
Date of Visit (months from initial diagnosis)	15	7	12
KPS	90	70	80
Mini Mental Status Exam Score			
History of seizures since last visit	No	No	No

Neurologic Deficit	Yes. Left eye difficulty.	No. Expressive aphasia.	No
Symptoms Notes	n/a. MRI followup.	Feels weak. Post Cyberknife followup.	Cognitive function has slowed slightly; progressive disease.
**Visit 12**			
Date of Visit (months from initial diagnosis)	17	8	14
KPS	80	70	80
Mini Mental Status Exam Score			
History of seizures since last visit	No	No	No
Neurologic Deficit	Yes. Left eye difficulty.	No. Expressive aphasia.	No
Symptoms Notes	Lower abdominal pain, perhaps chronic prostatitus. MRI followup.	Trouble with memory and communication. MRI scan followup.	Further clear increase in tumor size.
**Visit 13**			
Date of Visit (months from initial diagnosis)	19	9	15
KPS	80	60	70
Mini Mental Status Exam Score			
History of seizures since last visit	No	No	No
Neurologic Deficit	Yes. Left eye difficulty.	No. Expressive aphasia.	No
Symptoms Notes	Graying over of his vision especially under fluorescent lighting, perhaps ocular seizures. Neuro-oncology followup.	n/a. MRI followup.	Clinical deterioration, GI toxicity from the VP-16.
**Visit 14**			
Date of Visit (months from initial diagnosis)	25	10	
KPS	90	50	
Mini Mental Status Exam Score			
History of seizures since last visit	No	Yes	

Neurologic Deficit	No	Yes. Left. Expressive aphasia, left sided weakness.	
Symptoms Notes	Right parietal glioblastoma (MGMT methylated), status post gross total resection, placement of a VP shunt, and completion of chemoradiation and 12 cycles of adjuvant temozolomide, dose reduced for myelosuppression. Clinically he is doing well on observation since a year ago and radiographically there is no evidence for recurrent disease.	Rapidly worsening neurological symptoms with worsening weakness on the left side.	
**Visit 15**			
Date of Visit (months from initial diagnosis)	27		
KPS	90		
Mini Mental Status Exam Score			
History of seizures since last visit	No		
Neurologic Deficit	No		
Symptoms Notes	Clinically he is doing well on observation since a year ago, and radiographically there is no evidence for recurrent disease.		
**Visit 16**			
Date of Visit (months from initial diagnosis)	29		
KPS	90		
Mini Mental Status Exam Score			
History of seizures since last visit	Yes		

Neurologic Deficit	Yes. Left. History of intermittent visual field impairment.		
Symptoms Notes	He states that he is doing well without significant deficit nor new or progressive symptoms. Clinically he has a stable left visual field deficit. He remains active walking 4 miles a day. He continues on a regimen of herbal supplementation including mushrooms. Denies headache, numbness, weakness, gait impairment.		
**Visit 17**			
Date of Visit (months from initial diagnosis)	32		
KPS	90		
Mini Mental Status Exam Score			
History of seizures since last visit	No		
Neurologic Deficit	No		
Symptoms Notes	He is doing well without significant deficit nor new or progressive symptoms. He has not had any recurrent seizures since restarting Keppra 1000 mg BID. His previous history of intermittent visual field impairment have decreased significantly, his last visual aura according approximately 2 weeks ago. Clinically he has a stable left visual field deficit.		

**Visit 18**			
Date of Visit (months from initial diagnosis)	34		
KPS	90		
Mini Mental Status Exam Score			
History of seizures since last visit	No		
Neurologic Deficit	No		
Symptoms Notes	He is accompanied by his wife and states that he is doing well without significant deficit nor new symptoms. His wife has noted some slowly progressive confusion and memory difficulty. Also reports persistent, moderately bothersome fatigue for which he naps frequently throughout the day. Denies headache, numbness, weakness, gait impairment.		
**Visit 19**			
Date of Visit (months from initial diagnosis)	37		
KPS	90		
Mini Mental Status Exam Score			
History of seizures since last visit	No		
Neurologic Deficit	No		

Symptoms Notes	States that he is doing well without significant deficit nor new symptoms. Also reports persistent, moderately bothersome fatigue for which he naps frequently. Clinically, he has a stable left visual field deficit. He remains active walking daily. Reports some increasing left lower extremity weakness.		
**Visit 20**			
Date of Visit (months from initial diagnosis)	40		
KPS	90		
Mini Mental Status Exam Score			
History of seizures since last visit	No		
Neurologic Deficit	No		
Symptoms Notes	He is doing well without significant deficit nor new symptoms.		
**Visit 21**			
Date of Visit (months from initial diagnosis)	43		
KPS	90		
Mini Mental Status Exam Score			
History of seizures since last visit	No		
Neurologic Deficit	No		
Symptoms Notes	Clinically, he is doing well without new or progressive symptoms. He continues on Keppra 1500 mg BID with few ocular visual auras.		

**Visit 22**			
Date of Visit (months from initial diagnosis)	47		
KPS			
Mini Mental Status Exam Score			
History of seizures since last visit	No		
Neurologic Deficit	No		
Symptoms Notes	Follow-up visit.		
**Clinical Trials**			
1	//	//	//

Patient ID	W38-1-1	W39-1-1	W40-1-1
**Patient Information**			
Gender	Female	Female	Male
Age at Initial Diagnosis	65	68	65
Weight	63.5 kg	68.4 kg	110.7 kg
Height	170.2 cm	152.4 cm	172.7 cm
KPS	70 (?)	60 (?)	100 (?)
Mini Mental Status Exam Score	26	n/d	n/d
History of Seizures Since Last Visit	Yes	No	No
Neurologic Deficit	Yes. Left double simultaneous extinction.	Yes. Left.	No
Presentation Symptoms	Left visual field hallucinations, difficulty with mental processing and with completing tasks, headaches, trouble with gait.	Left-sided weakness.	History of increasing confusion, difficulty with numbers/letters, telling time, and difficulty reading.
Time to Progression	236 days	97 days	NA
Overall Survival	311 days	880 days	184 days
Time to Last Follow-up	NA	NA	NA
Cause Of Death	Tumor progression	Tumor progression	Tumor progression
**Tumor Information**			
**Tumor 1**			

Date (months from initial diagnosis)	0	0	0
Diagnosis	Astrocytic tumor	Astrocytic tumor	Astrocytic tumor
Grade	Glioblastoma WHO grade IV	Glioblastoma WHO grade IV	Glioblastoma WHO grade IV
Location	Righ temporal	Right Frontal	Left Parietal
**Medications**			
**Medication 1**			
Category	Anti-epileptic	Anti-epileptic	Steroids
Drug Name	Phenytoin	KEPPRA	Dexamethasone
Start (months from initial diagnosis)	-1	0	0
Total Daily Dosage		750 mg twice a day oral	4 mg 4 times a day oral
End (months from initial diagnosis)		6	0
**Medication 2**			
Category	Anti-epileptic	Cholesterol Lowering	Anti-epileptic
Drug Name	KEPPRA	Simvastatin	KEPPRA
Start (months from initial diagnosis)	-1	0	0
Total Daily Dosage	750 mg oral twice a day	80 mg at bedtime oral	1000 mg twice a day oral
End (months from initial diagnosis)	0	0	0
**Medication 3**			
Category	Cholesterol lowering	Steroids	Cardiotonic
Drug Name	Simvastatin	Dexamethasone	Warfarin
Start (months from initial diagnosis)	-1	0	0
Total Daily Dosage	10 mg oral daily	4mg/1mL injection 4mg 4 times a day	5 mg daily oral
End (months from initial diagnosis)	1	0	0
**Medication 4**			
Category	Anti-depressant	Antihypertensive	Antihypertensive
Drug Name	Citalopram (CELEXA)	Lisinopril	diltiazem
Start (months from initial diagnosis)	0	0	0
Total Daily Dosage	10 mg oral daily	20 mg daily oral	120 mg daily oral
End (months from initial diagnosis)	0	4	0
**Medication 5**			
Category	Steroids	Steroids	Steroids
Drug Name	Dexamethasone	Dexamethasone	Dexamethasone
Start (months from initial diagnosis)	0	0	0

Total Daily Dosage	4mg/1mL injection 4mg 4 times a day	4 mg 4 times a day, 2 mg 4 times a day till 1 mg daily oral	4mg/1mL injection 4mg 4 times a day, 3 times a day
End (months from initial diagnosis)	0	1	0
**Medication 6**			
Category	Anti-epileptic	Cholesterol Lowering	Anti-epileptic
Drug Name	KEPPRA	Simvastatin	KEPPRA
Start (months from initial diagnosis)	0	0	0
Total Daily Dosage	750 mg in NS (0.9% NaCl) IV solution 100 mL	40 mg at bedtime oral	1000 mg in NS(0.9% NaCl) IV solution 100 mg twice a day
End (months from initial diagnosis)	0	5	0
**Medication 7**			
Category	Steroids	Multi-vitamin	Antihypertensive
Drug Name	Dexamethasone	THERA MULTI-VITAMIN	Diltiazem
Start (months from initial diagnosis)	0	4	0
Total Daily Dosage	4mg/1mL injection 10mg 4 times	5 ml daily oral	30 mg 4 times a day oral
End (months from initial diagnosis)	0	4	0
**Medication 8**			
Category	Steroids	Anti-epileptic	Antihypertensive
Drug Name	Dexamethasone	KEPPRA	Diltiazem
Start (months from initial diagnosis)	0	6	0
Total Daily Dosage	4mg/1mL injection 6mg 4 times	100mg/mL 750 mg oral solution	120 mg QPM oral
End (months from initial diagnosis)	0	23	0
**Medication 9**			
Category	Steroids	Cholesterol Lowering	
Drug Name	Dexamethasone	Simvastatin	
Start (months from initial diagnosis)	0	9	
Total Daily Dosage	4mg/1mL injection 4mg 4 times a day	40 mg at bedtime oral	
End (months from initial diagnosis)	0	13	
**Medication 10**			
Category	Steroids	Anti-epileptic	
Drug Name	Dexamethasone	KEPPRA	
Start (months from initial diagnosis)	0	23	

Total Daily Dosage	4mg/1mL injection 2mg 4 times a day	750 mg twice a day oral	
End (months from initial diagnosis)	0	24	
**Medication 11**			
Category	Anti-epileptic	Anti-epileptic	
Drug Name	KEPPRA	KEPPRA	
Start (months from initial diagnosis)	0	24	
Total Daily Dosage	750 mg oral twice a day	1000-750 mg in NS(0.9% NaCl) IV solution 100 mg twice a day	
End (months from initial diagnosis)	0	24	
**Medication 12**			
Category	Anti-epileptic	Anti-epileptic	
Drug Name	Clonazepam (KLONOPIN)	KEPPRA	
Start (months from initial diagnosis)	0	24	
Total Daily Dosage	0.5 mg daily oral	1500 mg twice a day oral	
End (months from initial diagnosis)	2	27	
**Medication 13**			
Category	Anti-depressant	Anti-epileptic	
Drug Name	Citalopram (CELEXA)	KEPPRA	
Start (months from initial diagnosis)	0	27	
Total Daily Dosage	30 mg oral daily	1500 mg in AM and 2000 mg in PM oral	
End (months from initial diagnosis)	1	28	
**Medication 14**			
Category	Steroids		
Drug Name	Dexamethasone		
Start (months from initial diagnosis)	6		
Total Daily Dosage	4mg by mouth twice on the day of treatment. Then taper to 2mg two times a day for one day, then 1mg two times a day for one day then 1 mg		
End (months from initial diagnosis)	8		
**Surgery**			
**Surgery 1**			

Date of Surgery (months from initial diagnosis)	0	0	0
Gliadel	No	No	No
Awake mapping	No	No	No
Stealth scan	Yes	Yes	Yes
**Tissue Diagnosis**			
**Tissue 1**			
Serial Number	W38-1-1, SN254	SN257, W39-1-1	W40-1-1, SN263
Diagnosis	Glioblastoma WHO grade IV	Glioblastoma WHO grade IV	Glioblastoma WHO grade IV
Location	Right Temporal	Right Frontal	Left Parietal
Extent	Subtotal resection 90%	Subtotal resection 90%	Complete resection
MRI confirmation	Yes	Yes	Yes
Notes	//	95+% resection	//
**Chemotherapy**			
**Medication 1**			
Drug Name	Temozolomide	Temozolomide	Temozolomide
Start (months from initial diagnosis)	1	0	1
Dosage	120mg	120mg	
End (months from initial diagnosis)	2	2	2
Description	Concurrent	Concurrent	daily oral during radiation
**Medication 2**			
Drug Name	Temozolomide	Temozolomide	
Start (months from initial diagnosis)	4	3	
Dosage	270mg	240mg	
End (months from initial diagnosis)	5	4	
Description	maintenance 5 day cycle	maintenance 5 day cycle	
**Medication 3**			
Drug Name	Temozolomide	Temozolomide	
Start (months from initial diagnosis)	5	5	
Dosage	350mg	220mg	

End (months from initial diagnosis)	8	9	
Description	maintenance 5 day cycle	maintenance 5 day cycle	
**Medication 4**			
Drug Name	Avastin	Temozolomide	
Start (months from initial diagnosis)	8	9	
Dosage		210mg	
End (months from initial diagnosis)	8	11	
Description	maintenance	maintenance 5 day cycle	
**Medication 5**			
Drug Name	Temozolomide	Avastin	
Start (months from initial diagnosis)	8	23	
Dosage	75mg	525 mg in NS(0.9% NaCl) IV solution 100 mg	
End (months from initial diagnosis)	8	27	
Description	maintenance 7 day cycle	intravenuos every 2 weeks	
**Radiation Therapy**			
**Medication 1**			
Type	IMRT	IMRT	IMRT
Start (months from initial diagnosis)	1	0	1
Dosage	4600cGy	4600cGy	4600cGy
End (months from initial diagnosis)	2	1	2
Description	Concurrent	Adjuvant Radiation	Concurrent
Fraction	23	23	//
**Medication 2**			
Type	IMRT	IMRT	IMRT
Start (months from initial diagnosis)	2	1	2
Dosage	1400cGy	1400cGy	1400cGy
End (months from initial diagnosis)	2	2	2
Description	Boost	Boost	Boost
Fraction	7	7	//
**Medication 3**			
Type	Stereotactic		
Start (months from initial diagnosis)	6		
Dosage	7500cGy		

End (months from initial diagnosis)	6		
Description	Cyberknife		
Fraction	5		
**Pathology**			
**Surgery 1**			
Date Of Report (months from initial diagnosis)	0	0	0
**Report**			
Location	Tissue: W38-1-1, SN254	Tissue: SN257, W39-1-1	Tissue: W40-1-1, SN263
Histopathology	Glioblastoma, WHO grade IV/IV	Glioblastoma	Glioblastoma
1p19q_deletion	Negative	Negative	Negative
EGFR	gain	amplification	gain
PTEN	Normal	loss	loss
MGMT PCR	methylated	methylated	unmethylated
MGMT IHC	negative	25%	40%
MGMT MS-MLPA:R1	methylated (0.82)	unmethylated (0.24)	methylated (0.53)
MGMT MS-MLPA:R2	methylated (0.79)	unmethylated (0.00)	unmethylated (0.10)
MGMT MS-MLPA:R3	methylated (0.79)	methylated (0.57)	unmethylated (0.00)
IDH1 mutation	wildtype	wildtype	wildtype
EGFR vIII	not detected		not detected
**Follow-up Clinic Visit**			
**Visit 1**			
Date of Visit (months from initial diagnosis)	0	0	2
KPS	80	90	30
Mini Mental Status Exam Score			
History of seizures since last visit	No	No	No
Neurologic Deficit	Yes. Left/Right. Left hemiparesis.	Yes. Left sided weakness.	No
Symptoms Notes	Left hemiparesis and left neglect, ptosis on the right, facial movement decreased on the right. Comprehensive rehabilitation followup.	Left sided weakness continues, but somewhat improved. Radiation planning.	They have decided to hold all the medical treatments and go with hospice care. Hospice care.

**Visit 2**			
Date of Visit (months from initial diagnosis)	3	3	
KPS	70	90	
Mini Mental Status Exam Score	26		
History of seizures since last visit	No	No	
Neurologic Deficit	Yes. Left hemiparesis.	Yes. Left sided weakness.	
Symptoms Notes	Left hemiparesis. MRI followup.	Area of abnormal contrast enhancement in MRI scan. Radiation completion.	
**Visit 3**			
Date of Visit (months from initial diagnosis)	3	4	
KPS		80	
Mini Mental Status Exam Score	28		
History of seizures since last visit	No	No	
Neurologic Deficit	Yes. Left sided weakness.	Yes. Left sided weakness.	
Symptoms Notes	DC Vax protocol, 1st injection.	Pneumonia, fairly significant mucositis. Delay Temodar for one month. Stable focus of abnormal contrast consistent with pseudo-progression. MRI followup.	
**Visit 4**			
Date of Visit (months from initial diagnosis)	4	5	
KPS	80	80	
Mini Mental Status Exam Score			
History of seizures since last visit	No	No	
Neurologic Deficit	Yes. Left sided weakness.	Yes. Left sided weakness.	
Symptoms Notes	3rd DC Vax injection.	Cough worsened, developed bronchitis. Temodar cycle 2 delayed.	

**Visit 5**			
Date of Visit (months from initial diagnosis)	5	5	
KPS	70	80	
Mini Mental Status Exam Score			
History of seizures since last visit	No	No	
Neurologic Deficit	Yes. Left/Right. Left weakness, right ptosis.	Yes. Left sided weakness.	
Symptoms Notes	New imaging findings are concerning for progressive disease. MRI followup.	Significant improvement in coughing. Restart Temodar.	
**Visit 6**			
Date of Visit (months from initial diagnosis)	5	8	
KPS	70	70	
Mini Mental Status Exam Score			
History of seizures since last visit	No	Yes	
Neurologic Deficit	No. Left sided weakness.	Yes. Left sided weakness.	
Symptoms Notes	4th DC Vax injection.	Seizure 2 months ago, another episode of shingles, upper left extremity affected. MRI followup.	
**Visit 7**			
Date of Visit (months from initial diagnosis)	7	9	
KPS	60	70	
Mini Mental Status Exam Score			
History of seizures since last visit	No	Yes	
Neurologic Deficit	Yes. Left. Decline of left motor function.	Yes. Left sided weakness.	
Symptoms Notes	Early progression of disease while on the DCVax protocol, decline of left motor function.	Some left handed swelling, mild abulia. MRI followup.	

**Visit 8**			
Date of Visit (months from initial diagnosis)	8	11	
KPS	70	70	
Mini Mental Status Exam Score			
History of seizures since last visit	No	No	
Neurologic Deficit	Yes. Left weakness.	Yes. Left sided weakness.	
Symptoms Notes	5th DC Vax injection.	Marked restriction on left shoulder movement, pruritic rash develops after each dosage of temodar.	
**Visit 9**			
Date of Visit (months from initial diagnosis)	8	13	
KPS	60	80	
Mini Mental Status Exam Score			
History of seizures since last visit	No	Yes	
Neurologic Deficit	Yes. Left sided weakness.	Yes. Left sided weakness.	
Symptoms Notes	Suffering further deterioration of function on her left side.	Slight improvement of rash, although temodar will be discontinued. Routine followup.	
**Visit 10**			
Date of Visit (months from initial diagnosis)	8	14	
KPS	50	80	
Mini Mental Status Exam Score			
History of seizures since last visit	No	No	
Neurologic Deficit	Yes. Left sided weakness.	No	
Symptoms Notes	Tumors continues to worsen despite Avastin treatment.	Patient is doing reasonably well from a clinical standpoint and the MRI scan shows stable disease.	

**Visit 11**			
Date of Visit (months from initial diagnosis)		16	
KPS		70	
Mini Mental Status Exam Score			
History of seizures since last visit		No	
Neurologic Deficit		Yes. Left sided weakness.	
Symptoms Notes		Marked decline in motory status, more confusion.	
**Visit 12**			
Date of Visit (months from initial diagnosis)		18	
KPS		60	
Mini Mental Status Exam Score			
History of seizures since last visit		No	
Neurologic Deficit		No	
Symptoms Notes		Has returned to baseline since treating her UTI. Repeat UA pending today, but preliminarily appears okay.	
**Visit 13**			
Date of Visit (months from initial diagnosis)		21	
KPS		60	
Mini Mental Status Exam Score			
History of seizures since last visit		No	
Neurologic Deficit		No	
Symptoms Notes		Stable neurological status; and if anything she is getting better. While there is a suggestion of progression on the most recent scan, this is subtle.	

**Visit 14**			
Date of Visit (months from initial diagnosis)		23	
KPS		50	
Mini Mental Status Exam Score			
History of seizures since last visit		No	
Neurologic Deficit		No	
Symptoms Notes		Her functional status is impaired and her mobility over the past year seems to have been in a decline.	
**Visit 15**			
Date of Visit (months from initial diagnosis)		25	
KPS		50	
Mini Mental Status Exam Score			
History of seizures since last visit		No	
Neurologic Deficit		No	
Symptoms Notes		Clinically, she has remained stable, with baseline limited mobility. She has not had any further seizures since her Keppra was increased to 1500 mg BID. Denies any new or progressive neurologic symptoms.	
**Visit 16**			
Date of Visit (months from initial diagnosis)		27	
KPS		50	
Mini Mental Status Exam Score			
History of seizures since last visit		Yes	
Neurologic Deficit		No	

Symptoms Notes		Clinically, she has been declining with respect to her mobility, cognition and ability to care for herself.	
**Clinical Trials**			
1	DCVax	//	//

Patient ID	W42-1-1	W43-1-1	W48-1-1
**Patient Information**			
Gender	Female	Female	Male
Age at Initial Diagnosis	60	62	52
Weight	89.994 kg	76.749 kg	87.8 kg
Height	172.45 cm	163.56 cm	177.8 cm
KPS	70 (?)	90 (?)	80 (?)
Mini Mental Status Exam Score		28	26
History of Seizures Since Last Visit	No	No	No
Neurologic Deficit	No	Yes. Left/Right. Speech difficulty.	No
Presentation Symptoms	One-month history of fainting spells, visual distortions, and tremor in right upper extremity.	Two-week history of headaches, cognitive and speech difficulty, nausea, vomiting, falls associated with dizziness.	History of headaches, nausea.
Time to Progression	NA	133 days	92 days
Overall Survival	197 days	300 days	456 days
Time to Last Follow-up	NA	NA	NA
Cause Of Death	Tumor progression	Tumor progression	Tumor progression
**Tumor Information**			
**Tumor 1**			
Date (months from initial diagnosis)	0	0	0
Diagnosis	Astrocytic tumor	Astrocytic tumor	Astrocytic tumor
Grade	Glioblastoma WHO grade IV	Glioblastoma WHO grade IV	Glioblastoma WHO grade IV
Location	Right Frontal-Temporal	Right Temporal	Right Parietal
**Tumor 2**			

Date (months from initial diagnosis)			7
Diagnosis			Astrocytic tumor
Grade			Glioblastoma WHO grade IV
Location			Right Temporal
**Medications**			
**Medication 1**			
Category	Steroids	Multi-vitamin	Steroids
Drug Name	Dexamethasone	ONE-A-DAY	Dexamethasone
Start (months from initial diagnosis)	-1	0	-1
Total Daily Dosage	4mg/mL intravenuos 4 mg 4 tiimes a day	1 cap daily oral	4mg/1mL injection 4mg 4 times a day
End (months from initial diagnosis)	-1	0	-1
**Medication 2**			
Category	Anti-epileptic	Steroids	Anti-epileptic
Drug Name	KEPPRA	Dexamethasone	KEPPRA
Start (months from initial diagnosis)	-1	0	-1
Total Daily Dosage	1000 mg in NS(0.9% NaCl) IV solution 100 mL twice a day	6 mg 4 times a day, 4 mg 4 times a day, 3 mg 4 times a day till 1 mg daily oral	750 mg in NS(0.9% NaCl) IV solution 100 m
End (months from initial diagnosis)	-1	1	-1
**Medication 3**			
Category	Steroids	Anti-epileptic	Anti-epileptic
Drug Name	Dexamethasone	KEPPRA	KEPPRA
Start (months from initial diagnosis)	-1	0	-1
Total Daily Dosage	4 mg twice a day oral	1000 mg twice a day oral	1000 mg twice a day oral
End (months from initial diagnosis)	0	0	0
**Medication 4**			
Category	Anti-epileptic	Anti-epileptic	Steroids
Drug Name	KEPPRA	KEPPRA	Dexamethasone
Start (months from initial diagnosis)	-1	0	-1
Total Daily Dosage	1000 mg twice a day oral	1000 mg in NS(0.9% NaCl) IV solution 100 mg twice a day	4 mg oral 4-3 times a day oral
End (months from initial diagnosis)	0	0	0
**Medication 5**			
Category		Anti-epileptic	Steroids

Drug Name		KEPPRA	Dexamethasone
Start (months from initial diagnosis)		0	0
Total Daily Dosage		1000 mg twice a day oral	1 mg 4 times a day oral
End (months from initial diagnosis)		7	0
**Medication 6**			
Category		Steroids	Steroids
Drug Name		Dexamethasone	Dexamethasone
Start (months from initial diagnosis)		5	0
Total Daily Dosage		4 mg once oral	2-1 mg 3-2-1 times a day oral
End (months from initial diagnosis)		//	0
**Medication 7**			
Category		Steroids	Anti-epileptic
Drug Name		Dexamethasone	KEPPRA XR
Start (months from initial diagnosis)		7	0
Total Daily Dosage		1 mg twice a day oral	1000 mg twice a day oral
End (months from initial diagnosis)		8	3
**Medication 8**			
Category			Steroids
Drug Name			Dexamethasone
Start (months from initial diagnosis)			7
Total Daily Dosage			2 mg 3 times a day oral
End (months from initial diagnosis)			7
**Medication 9**			
Category			Anti-epileptic
Drug Name			KEPPRA
Start (months from initial diagnosis)			7
Total Daily Dosage			1000 mg twice a day oral
End (months from initial diagnosis)			8
**Medication 10**			
Category			Steroids
Drug Name			Dexamethasone
Start (months from initial diagnosis)			7
Total Daily Dosage			4mg/1mL injection 2-4mg 3-4 times a day
End (months from initial diagnosis)			7

**Medication 11**			
Category			Steroids
Drug Name			Dexamethasone
Start (months from initial diagnosis)			7
Total Daily Dosage			from 4 mg 3 times a day till 1 mg daily oral
End (months from initial diagnosis)			8
**Medication 12**			
Category			Steroids
Drug Name			Dexamethasone
Start (months from initial diagnosis)			14
Total Daily Dosage			8mg twice a day oral
End (months from initial diagnosis)			15
**Medication 13**			
Category			Antihypertensive
Drug Name			Lisinopril
Start (months from initial diagnosis)			14
Total Daily Dosage			20 mg daily oral
End (months from initial diagnosis)			15
**Medication 14**			
Category			Anti-epileptic
Drug Name			KEPPRA
Start (months from initial diagnosis)			14
Total Daily Dosage			500 mg twice a day oral
End (months from initial diagnosis)			15
**Surgery**			
**Surgery 1**			
Date of Surgery (months from initial diagnosis)	0	0	0
Gliadel	No	No	No
Awake mapping	No	No	No
Stealth scan	Yes	Yes	Yes
**Surgery 2**			
Date of Surgery (months from initial diagnosis)			7
Gliadel			No

Awake mapping			No
Stealth scan			Yes
**Tissue Diagnosis**			
**Tissue 1**			
Serial Number	W42-1-1, SN285	W43-1-1, SN286	SN309, W48-1-1
Diagnosis	Glioblastoma WHO grade IV	Glioblastoma WHO grade IV	Glioblastoma WHO grade IV
Location	Right Frontal-Temporal	Right Temporal	Right Parietal
Extent	Complete resection	Complete resection	Complete resection
MRI confirmation	No	Yes	Yes
Notes	Concerning for a small amount of residual tumor	No abnormal enhancement demonstrated to suggest residual tumor	Apparent gross total resection, no residual tumor
**Tissue 2**			
Serial Number			SN332, W48-2-1
Diagnosis			Glioblastoma WHO grade IV
Location			Right Temporal
Extent			Complete resection
MRI confirmation			Yes
Notes			//
**Chemotherapy**			
**Medication 1**			
Drug Name	Temozolomide	Temozolomide	Temozolomide
Start (months from initial diagnosis)	1	0	1
Dosage	150mg	140mg	145mg
End (months from initial diagnosis)	2	2	2
Description	Concurrent	concurrent	concurrent
**Medication 2**			
Drug Name	Temozolomide	Temozolomide	Temozolomide
Start (months from initial diagnosis)	3	2	4
Dosage	100mg	280mg	290mg
End (months from initial diagnosis)		5	4

Description	maintenance 5d cycles	maintenance 5 day cycle	daily oral
**Medication 3**			
Drug Name		Temozolomide	Temozolomide
Start (months from initial diagnosis)		5	5
Dosage		140mg	400mg
End (months from initial diagnosis)		7	7
Description		maintenance 21 day cycle	daily oral
**Medication 4**			
Drug Name		Avastin	Temozolomide
Start (months from initial diagnosis)		7	8
Dosage		//	195mg
End (months from initial diagnosis)		8	9
Description		intravenuos every 2 weeks	daily oral
**Medication 5**			
Drug Name			Avastin
Start (months from initial diagnosis)			9
Dosage			753 mg in NS(0.9% NaCl) IV solution 100 mL
End (months from initial diagnosis)			14
Description			every 2 weeks
**Medication 6**			
Drug Name			Etoposide
Start (months from initial diagnosis)			12
Dosage			50mg
End (months from initial diagnosis)			12
Description			daily oral
**Medication 7**			
Drug Name			Etoposide
Start (months from initial diagnosis)			13
Dosage			100mg
End (months from initial diagnosis)			14
Description			daily oral
**Radiation Therapy**			

**Medication 1**			
Type	//	//	IMRT
Start (months from initial diagnosis)	1	0	1
Dosage	//	6300cGy	6000cGy
End (months from initial diagnosis)	2	2	2
Description	//	//	The right parietal occipital tumor bed, was treated with a 5 field IMRT technique
Fraction	//	//	30
**Medication 2**			
Type		Stereotactic	
Start (months from initial diagnosis)		7	
Dosage		2400cGy	
End (months from initial diagnosis)		7	
Description		Cyberknife/Adjuvant	
Fraction		3	
**Pathology**			
**Surgery 1**			
Date Of Report (months from initial diagnosis)	0	0	0
**Report**			
Location	Tissue: W42-1-1, SN285	Tissue: W43-1-1, SN286	Tissue: SN309, W48-1-1
Histopathology	Glioblastoma	Glioblastoma	Glioblastoma
1p19q_deletion			
EGFR			
PTEN			
MGMT PCR			
MGMT IHC			
MGMT MS-MLPA:R1	methylated (0.36)	methylated (0.51)	unmethylated (0.26)
MGMT MS-MLPA:R2	unmethylated (0.24)	unmethylated (0.06)	unmethylated (0.09)
MGMT MS-MLPA:R3	methylated (0.47)	unmethylated (0.01)	unmethylated (0.05)
IDH1 mutation	wildtype	wildtype	R132G
EGFR vIII	detected		not detected

**Surgery 2**			
Date Of Report (months from initial diagnosis)			7
**Report**			
Location			Tissue: SN332, W48-2-1
Histopathology			Glioblastoma
1p19q_deletion			
EGFR			
PTEN			
MGMT PCR			
MGMT IHC			
MGMT MS-MLPA:R1			methylated (0.37)
MGMT MS-MLPA:R2			unmethylated (0.05)
MGMT MS-MLPA:R3			unmethylated (0.03)
IDH1 mutation			wildtype
EGFR vIII			not detected
**Follow-up Clinic Visit**			
**Visit 1**			
Date of Visit (months from initial diagnosis)	0	0	0
KPS	90		
Mini Mental Status Exam Score			
History of seizures since last visit	No	No	No
Neurologic Deficit	No	No	Yes. Left hemianopsia.
Symptoms Notes	Suture removal.	Suture removal.	Left hemianopsia, occasionally hears a "sqeaky" noise inside his head near the incision site. Suture removal.
**Visit 2**			
Date of Visit (months from initial diagnosis)	0	0	0
KPS	80		
Mini Mental Status Exam Score		28	26

History of seizures since last visit	Yes	No	No
Neurologic Deficit	Yes. Right arm tremor.	No	Yes. Left hemianopsia.
Symptoms Notes	Seizures, trembling of the hand and arms (right), headaches, dizziness, fainting, numbness, tingling, trouble with balance. Radiation planning.	Radiation planning.	Radiation planning.
**Visit 3**			
Date of Visit (months from initial diagnosis)	1	1	0
KPS	80	90	90
Mini Mental Status Exam Score			
History of seizures since last visit	Yes	No	No
Neurologic Deficit	Yes. Right arm tremor.	No	Yes. Left hemianopsia.
Symptoms Notes	Radiation evaluation.	Mid radiation visit.	Left hemianopsia, very slight left lower extremity weakness. Radiation planning.
**Visit 4**			
Date of Visit (months from initial diagnosis)	3	2	1
KPS	70	90	90
Mini Mental Status Exam Score			
History of seizures since last visit	No	No	No
Neurologic Deficit	Yes. Right arm tremor.	No	Yes. Left hemianopsia.
Symptoms Notes	Fatigue, right leg edema, right arm tremor. Post radiation.	Post radiation visit.	Occasional brief headaches. His left-sided visual problems have improved. Mid radiation followup.
**Visit 5**			
Date of Visit (months from initial diagnosis)	3	4	3

KPS	60	80	90
Mini Mental Status Exam Score			
History of seizures since last visit	Yes	No	No
Neurologic Deficit	Yes. Right arm tremor.	No	Yes. Left hemianopsia.
Symptoms Notes	Fatigue, edema in right leg, headache, backache, nausea, vomiting. Symptons concerning for progressive disease. TMZ evaluation.	Signs of tumor progression. MRI followup.	Patient discontinued keppra without being instructed to do so. Post radiation followup.
**Visit 6**			
Date of Visit (months from initial diagnosis)		5	3
KPS		80	90
Mini Mental Status Exam Score			
History of seizures since last visit		No	No
Neurologic Deficit		No	Yes. Left hemianopsia.
Symptoms Notes		Increasing symptoms that reflect the worsening contrast enhancement. Switching from 5 day to 21 day Temodar regimen. Starting Avastin.	DC Vax protocol, 1st injection today.
**Visit 7**			
Date of Visit (months from initial diagnosis)		7	3
KPS		90	90
Mini Mental Status Exam Score			
History of seizures since last visit		Yes	No
Neurologic Deficit		No	No
Symptoms Notes		Drastic improvement of enhancement. 2 month followup.	No residual visual field cuts. Radiation Oncology followup.

**Visit 8**			
Date of Visit (months from initial diagnosis)		8	9
KPS		70	90
Mini Mental Status Exam Score			
History of seizures since last visit		No	No
Neurologic Deficit		No	No
Symptoms Notes		Headaches, poor balance, numb feet, and drowsiness. Stopped Temodar after Cyberknife. 2 month followup.	Denies seizures, headache, cognitive changes, gait impairment.
**Visit 9**			
Date of Visit (months from initial diagnosis)			10
KPS			90
Mini Mental Status Exam Score			
History of seizures since last visit			No
Neurologic Deficit			No
Symptoms Notes			Reports some memory loss, fatigue, depression, trouble sleeping, and anxiety.
**Visit 10**			
Date of Visit (months from initial diagnosis)			12
KPS			90
Mini Mental Status Exam Score			
History of seizures since last visit			No
Neurologic Deficit			No
Symptoms Notes			Returns today for surveillance scan and reports doing well without new or progressive symptoms.

**Visit 11**			
Date of Visit (months from initial diagnosis)			14
KPS			70
Mini Mental Status Exam Score			
History of seizures since last visit			No
Neurologic Deficit			Yes. Left.
Symptoms Notes			Clinically, he continues to have a left hemiparesis which is modestly more apparent compared to a few weeks ago. He denies headaches and is able to ambulate independently with some unsteadiness. He denies any history of seizures.
**Clinical Trials**			
1	//	//	DCVax - 1st injection in 3 months from initial diagnosis.

Patient ID	W53-1-1	W54-1-1	W55-1-1
**Patient Information**			
Gender	Male	Male	Male
Age at Initial Diagnosis	55	58	53
Weight	69.673 kg	154 kg	95 kg
Height	171.5 cm	178 cm	185 cm
KPS	90 (?)	70 (?)	100 (?)
Mini Mental Status Exam Score			
History of Seizures Since Last Visit	No	Yes	No
Neurologic Deficit	No	No	No

Presentation Symptoms	Three month history of word finding difficulties.	Three week history of headaches and expressive aphasia.	Mild headaches, memory loss.
Time to Progression	312 days	NA	NA
Overall Survival	NA	62 days	NA
Time to Last Follow-up	652 days	NA	2 days
Cause Of Death	//	Tumor progression	//
**Tumor Information**			
**Tumor 1**			
Date (months from initial diagnosis)	0	0	0
Diagnosis	Astrocytic tumor	Astrocytic tumor	Astrocytic tumor
Grade	Glioblastoma WHO grade IV	Glioblastoma WHO grade IV	Glioblastoma WHO grade IV
Location	Left Temporal	Left Temporal	Right Temporal
**Medications**			
**Medication 1**			
Category	Anti-epileptic	Steroids	Steroids
Drug Name	KEPPRA	Dexamethasone	Dexamethasone
Start (months from initial diagnosis)	0	0	0
Total Daily Dosage	1000 mg twice a day oral	from 4 mg twice a day till 1 mg daily oral	4mg/1mL injection 4mg 4 times a day
End (months from initial diagnosis)	1	1	0
**Medication 2**			
Category	Steroids	Antihypertensive	Steroids
Drug Name	Dexamethasone	Lisinopril	Dexamethasone
Start (months from initial diagnosis)	0	0	0
Total Daily Dosage	2 mg twice a day oral	10 mg daily oral	4 mg 4 times a day oral
End (months from initial diagnosis)	1	1	0
**Medication 3**			
Category	Anti-epileptic	Anti-depressant	Steroids
Drug Name	KEPPRA	Quetiapine	Dexamethasone
Start (months from initial diagnosis)	1	0	0
Total Daily Dosage	500 mg twice a day oral	50 mg twice a day oral	from 3 mg 3 times a day till 2 mg daily oral
End (months from initial diagnosis)	3	1	0

**Medication 4**			
Category	Anti-epileptic	Antihypertensive	
Drug Name	KEPPRA	Lisinopril	
Start (months from initial diagnosis)	14	1	
Total Daily Dosage	1000 mg once oral	20 mg daily oral	
End (months from initial diagnosis)	//	1	
**Surgery**			
**Surgery 1**			
Date of Surgery (months from initial diagnosis)	0	0	0
Gliadel	//	No	No
Awake mapping	//	No	No
Stealth scan	//	No	Yes
**Tissue Diagnosis**			
**Tissue 1**			
Serial Number	W53-1-1, SN331	W54-1-1, SN347	W55-1-1, SN357
Diagnosis	Glioblastoma WHO grade IV	Glioblastoma WHO grade IV	Glioblastoma WHO grade IV
Location	Left Temporal	Left Temporal	Right Temporal
Extent	Complete resection	Complete resection	Complete resection
MRI confirmation	Yes	//	Yes
**Chemotherapy**			
**Medication 1**			
Drug Name	Temozolomide	//	//
Start (months from initial diagnosis)	1		
Dosage	140mg		
End (months from initial diagnosis)	2		
Description	daily oral		
**Medication 2**			
Drug Name	Temozolomide		
Start (months from initial diagnosis)	4		
Dosage	340mg		
End (months from initial diagnosis)	9		

Description	every 2 weeks for 5 days oral		
**Medication 3**			
Drug Name	Temozolomide		
Start (months from initial diagnosis)	9		
Dosage	140mg		
End (months from initial diagnosis)	10		
Description	everyday for 21 days oral		
**Medication 4**			
Drug Name	Etoposide		
Start (months from initial diagnosis)	12		
Dosage	100mg		
End (months from initial diagnosis)	19		
Description	daily oral		
**Medication 5**			
Drug Name	Etoposide		
Start (months from initial diagnosis)	19		
Dosage	100mg		
End (months from initial diagnosis)	22		
Description	daily oral		
**Medication 6**			
Drug Name	Etoposide		
Start (months from initial diagnosis)	22		
Dosage	100mg		
End (months from initial diagnosis)	22		
Description	daily oral		
**Radiation Therapy**			
**Medication 1**			
Type	IMRT	//	IMRT
Start (months from initial diagnosis)	1		0
Dosage	4600cGy		4500cGy
End (months from initial diagnosis)	2		2

Description	to the temporal lobe tumor bed		GBM
Fraction	23		25
**Medication 2**			
Type	IMRT		IMRT
Start (months from initial diagnosis)	2		2
Dosage	1400cGy		1620cGy
End (months from initial diagnosis)	2		2
Description	Boost		GBM boost
Fraction	7		9
**Medication 3**			
Type	IMRT		
Start (months from initial diagnosis)	10		
Dosage	21Gy		
End (months from initial diagnosis)	//		
Description	L medial / inferior temporal lobe recurrence		
Fraction	1 for medial, and 1 for inferior		
**Pathology**			
**Surgery 1**			
Date Of Report (months from initial diagnosis)	0	0	0
**Report**			
Location	Tissue: W53-1-1, SN331	Tissue: W54-1-1, SN347	Tissue: W55-1-1, SN357
Histopathology	Glioblastoma WHO grade IV	Glioblastoma WHO grade IV	Glioblastoma WHO grade IV
1p19q_deletion			
EGFR			
PTEN			
MGMT PCR		unmethylated	
MGMT IHC			
MGMT MS-MLPA:R1	methylated (0.81)	methylated (0.47)	methylated (0.30)
MGMT MS-MLPA:R2	unmethylated (0.29)	unmethylated (0.15)	unmethylated (0.12)
MGMT MS-MLPA:R3	methylated (0.45)	unmethylated (0.04)	methylated (0.67)

IDH1 mutation	wildtype	wildtype	wildtype
EGFR vIII	not detected	not detected	detected-ACT+
**Follow-up Clinic Visit**			
**Visit 1**			
Date of Visit (months from initial diagnosis)	0	0	0
KPS	90	90	100
Mini Mental Status Exam Score			
History of seizures since last visit	Yes	No	No
Neurologic Deficit	Yes. Right leg weakness.	No	No
Symptoms Notes	Has undergone near gross total resection of a large left temporal GBM.	He began having severe headaches about 3 weeks ago. He was also having expressive aphasia.	Clinically he is without deficit, headaches, nor history of seizures.
**Visit 2**			
Date of Visit (months from initial diagnosis)	1	1	0
KPS	90	70	100
Mini Mental Status Exam Score			
History of seizures since last visit	No	No	No
Neurologic Deficit	No	Yes. Right-sided weakness.	No
Symptoms Notes	Has never had a seizure. Neurologic and general review of systems is otherwise negative in detail.	Continues to have stable expressive aphasia and right-sided weakness.	Clinically, he is doing quite well without neurologic deficit nor history of seizures. Has established care with local providers for both radiation and chemotherapy and will plan to transfer coordination for the DCVax study out of state.
**Visit 3**			
Date of Visit (months from initial diagnosis)	3		
KPS	100		
Mini Mental Status Exam Score			

History of seizures since last visit	No		
Neurologic Deficit	No		
Symptoms Notes	He is doing well. We will move forward with adjuvant TMZ.		
**Visit 4**			
Date of Visit (months from initial diagnosis)	4		
KPS	90		
Mini Mental Status Exam Score			
History of seizures since last visit	No		
Neurologic Deficit	No		
Symptoms Notes	Is doing well. He had no other problems with chemotherapy. He denies headaches or seizures.		
**Visit 5**			
Date of Visit (months from initial diagnosis)	5		
KPS	90		
Mini Mental Status Exam Score			
History of seizures since last visit	No		
Neurologic Deficit	No		
Symptoms Notes	Had a migraine headache about two weeks ago which resolved with immitrex. One day prior to the migraine, he noted numbness in each of his finger tips which lasted about 10 minutes. He had no other symptoms.		
**Visit 6**			
Date of Visit (months from initial diagnosis)	6		

KPS	90		
Mini Mental Status Exam Score			
History of seizures since last visit	No		
Neurologic Deficit	No		
Symptoms Notes	He has had no headaches or seizures and no new neurologic deficits.		
**Visit 7**			
Date of Visit (months from initial diagnosis)	7		
KPS	100		
Mini Mental Status Exam Score			
History of seizures since last visit	No		
Neurologic Deficit	No		
Symptoms Notes	He continues to feel he is improving in terms of his cognitive function. He has no new neurologic concerns or complaints. He denies headaches and has had no seizures.		
**Visit 8**			
Date of Visit (months from initial diagnosis)	9		
KPS	90		
Mini Mental Status Exam Score			
History of seizures since last visit	No		
Neurologic Deficit	No		
Symptoms Notes	No changes.		
**Visit 9**			
Date of Visit (months from initial diagnosis)	10		
KPS	80		
Mini Mental Status Exam Score			
History of seizures since last visit	No		
Neurologic Deficit	No		

Symptoms Notes	Disease progression. Stop TMZ, move to radiosurgery of two lesions.		
**Visit 10**			
Date of Visit (months from initial diagnosis)	12		
KPS	70		
Mini Mental Status Exam Score			
History of seizures since last visit	Yes		
Neurologic Deficit	No		
Symptoms Notes	Two months ago he received gamma knife to a progressing nodular focus of enhancement along the medial aspect of the surgical cavity. He was doing well then developed worsening speech difficulties and right sided weakness. He otherwise denies headaches, seizures, or other neurologic deficits.		
**Visit 11**			
Date of Visit (months from initial diagnosis)	14		
KPS	70		
Mini Mental Status Exam Score			
History of seizures since last visit	No		
Neurologic Deficit	No		

Symptoms Notes	Has been working with PT and has had improvement in right sided strength. He is more fatigued since starting VP16 and weakness is more pronounced with fatigue. He denies nausea. He otherwise has no new concerns. He denies headaches. He has had no new or progressive symptoms.		
**Visit 12**			
Date of Visit (months from initial diagnosis)	15		
KPS	70		
Mini Mental Status Exam Score			
History of seizures since last visit	No		
Neurologic Deficit	No		
Symptoms Notes	Has now completed three cycles of VP 16 which he is tolerating with minimal side effects other than fatigue. He feels both his speech and right sided strength are improving although right hand and arm remain weak. He continues to take Plavix daily for stroke prevention.		
**Visit 13**			
Date of Visit (months from initial diagnosis)	16		
KPS	70		
Mini Mental Status Exam Score			
History of seizures since last visit	No		
Neurologic Deficit	No		

Symptoms Notes	MRI showed increased enhancement along the previously treated gamma knife region. Recommended patient start Etoposide in the event the enhancement reflects tumor progression. He has now completed 4 cycles which he is tolerating well with his only complaint being fatigue. He denies headaches and has had no seizures. Strength and balance are stable.		
**Visit 14**			
Date of Visit (months from initial diagnosis)	17		
KPS	70		
Mini Mental Status Exam Score			
History of seizures since last visit	No		
Neurologic Deficit	No		
Symptoms Notes	Doing well clinically and radiographically.		
**Visit 15**			
Date of Visit (months from initial diagnosis)	18		
KPS	70		
Mini Mental Status Exam Score			
History of seizures since last visit	No		
Neurologic Deficit	No		
Symptoms Notes	Is doing okay, no new concerns or complaints.		
**Visit 16**			
Date of Visit (months from initial diagnosis)	19		

KPS	70		
Mini Mental Status Exam Score			
History of seizures since last visit	No		
Neurologic Deficit	No		
Symptoms Notes	Alert, fluent, grip improved, gait ok. Neuroimaging from today reviewed in clinic: stable. Doing well clinically and radiographically. Two additional cycles of VP16.		
**Visit 17**			
Date of Visit (months from initial diagnosis)	21		
KPS	70		
Mini Mental Status Exam Score			
History of seizures since last visit	No		
Neurologic Deficit	No		
Symptoms Notes	//		
**Visit 18**			
Date of Visit (months from initial diagnosis)	22		
KPS	70		
Mini Mental Status Exam Score			
History of seizures since last visit	No		
Neurologic Deficit	No		
Symptoms Notes	No new problems, he thinks his arm is still slowly improving.		
**Visit 19**			
Date of Visit (months from initial diagnosis)	23		
KPS			
Mini Mental Status Exam Score			

History of seizures since last visit	No		
Neurologic Deficit	No		
Symptoms Notes	Follow-up visit.		
**Clinical Trials**			
1	//	DCVax	DCVax

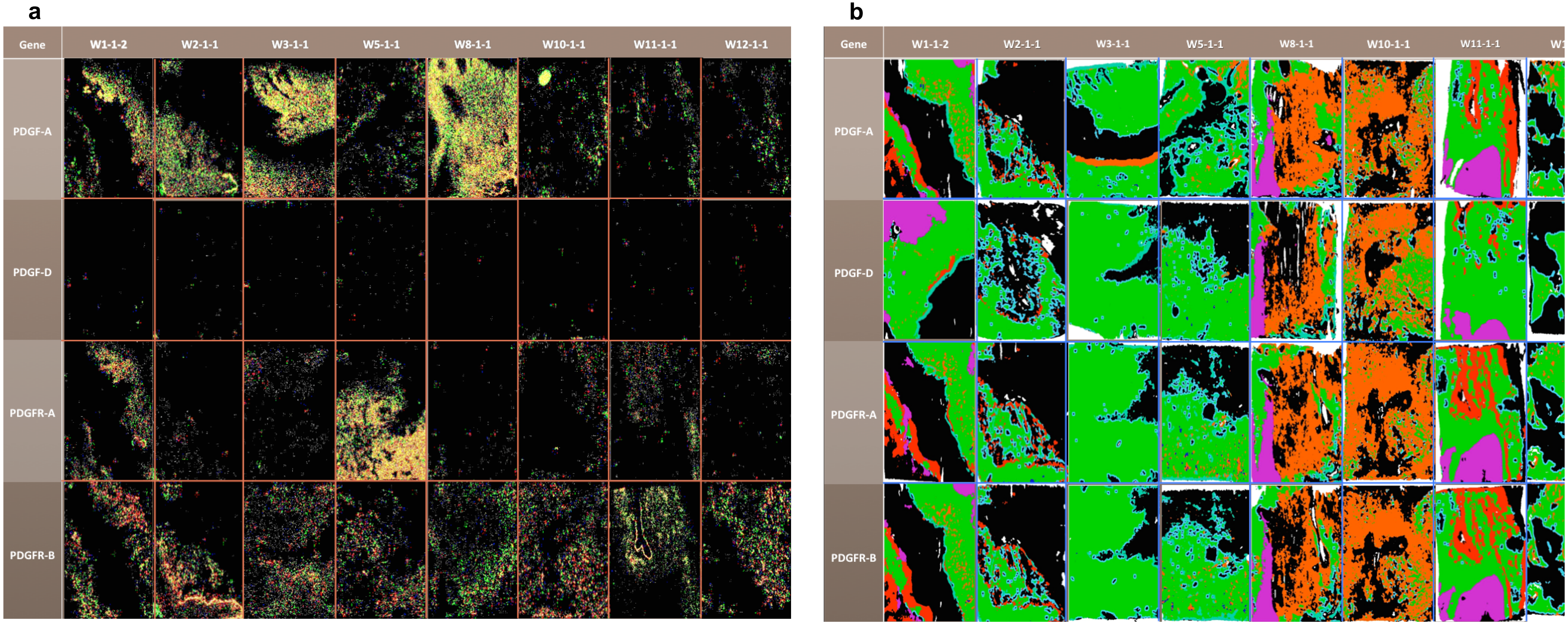

Supplementary Figure S1: Illustrative panels of probe specific hybridization for each PDGF gene based on microarray analysis and the correspondent Glioblastoma Multiforme (GBM) regions annotation. (a) Compilation of images of probe specific hybridization for PDGFA, PDGFD, PDGFRA and PDGFRB genes in GBM tissue sections of eight of the adult human donors included in this study (W1-1-2, W2-1-1, W3-1-1, W5-1-1, W8-1-1, W10-1-1, W11-1-1 and W12-1-1). *PDGFRB* images stand out in comparison to the other genes data as the expression appears to be more intense and to comprise wider areas of the tissue section in all donors samples. Images of *PDGFD* expression show a peculiar shape: expression loci appear as separate points, whereas the other genes expression patterns show large blocks of close expression loci; **(b)** Correspondent tissue sections with annotation of the GBM regions analysed in this study: Infiltrating Tumour (in purple), Cellular Tumour (in green), Perinecrotic Zone (in blue), Hyperplastic Blood Vessels in Cellular Tumour (in orange), Microvascular Proliferation (in red). It is not possible to visually observe specific patterns of the PDGF genes expression in each region. All data is available online on the Ivy Glioblastoma Atlas Project website [glioblastoma.alleninstitute.org/rnaseq/search/index.html].

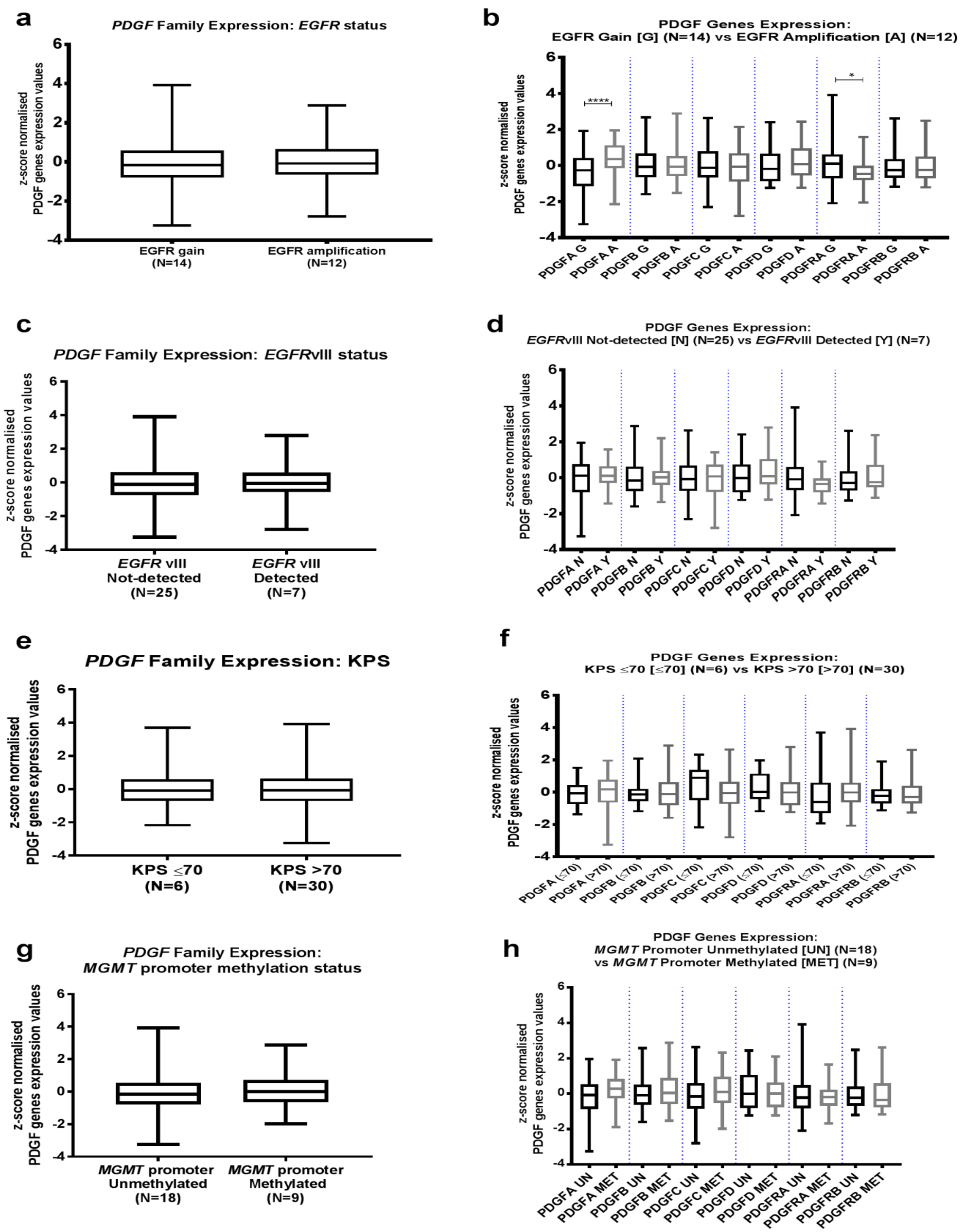

Supplementary Figure S2: Analysis of PDGF family expression in relation to genomic and clinical factors. No significant differential *PDGF* expression was observed as to Epidermal Growth Factor Receptor (EGFR) gene status **(a, b)**, presence or absence of the EGFRvIII mutation **(c, d)**, Karnofsky Performance Status score **(e, f)** or methylation status of O^6-methylguanine-DNA-methyltransferase (MGMT) gene promoter **(g, h).** Data available online on the Ivy Glioblastoma Atlas Project website [glioblastoma.alleninstitute.org/rnaseq/search/index.html] and Ivy GAP Clinical and Genomic Database [ivygap.swedish.org/home].
